# Supplementary material for: Unscheduled and out-of-hours care for people in their last year of life: a retrospective cohort analysis of national datasets
Source: BMJ Open. 2020 Nov 23;10(11):e041888. doi: 10.1136/bmjopen-2020-041888 (PMC7684800; doi:10.1136/bmjopen-2020-041888)
Supplement: Supplementary data [file bmjopen-2020-041888supp001.pdf]

Supplementary tables 1-4

Supplementary table 1. List of ICD10 codes for underlying causes of death grouped into three main categories of conditions with potential palliative care needs (referred to as "illness trajectories")

**Illness trajectories [ITs]**	Sub-ITs	ICD10 codes (v.2010)
**IT1. Cancer *("Clear terminal phase")***		
	1.1 Malignant Neoplasms	C00-C97
**IT2. Organ failure *("Intermittent")***		
	2.1 Endocrine and metabolic diseases	E00-E90<br>*Except: E46*
	2.2 Diseases of the circulatory system	I00-I99<br>*Except: I46*<br>*(I60-I67)[65+]* ****(1/3)**[1]*<br>*I69*
	2.3 Diseases of the respiratory system	J00-J99.8<br>*Except: (J06, J09-J16, J18)[65+]* ****(1/3)**[2]*<br>*(J20-J22)[65+]* ****(1/3)**[3]*<br>*J69.0, J96*
	2.4 Diseases of liver	K70-K77, B18
	2.5 Kidney disease	N17, N18, N28
	2.6 Diseases of the digestive system	K00-K93.8<br>*Except: K70-K77*
	2.7 Other diseases of the genitourinary system	N00-N99.9<br>*Except: N17, N18, N28*
**IT3a. Frailty, Progressive neurological condition [80+]**		

("Gradually dwindling")		
	3.1 Dementia and mental disorders [80+]	A81.0, F01, F03-F07, F09, G30, G31
	3.2 Progressive neurological diseases [80+]	G10, G12.2, G20, G35, G23.1, G90.3
	3.3 Cerebrovascular diseases [80+]	(I60-I67)$_{[80+]}$***(1/3)**, I69
	3.4 Some ill-defined causes [80+]	R26, R29.6, R53, R54, R55, R63, R64
		I46, J96, R96, R98, R99, W00, W03-08, W10, W18
	3.5 Malnutrition [80+]	E46
	3.6 Infectious causes / Influenza / Pneumonia [80+]	A15-A19.9, B90-B90.9, B20-B24<br>(J06, J09-J16, J18)$_{[80+]}$***(1/3)**<br>(J20-J22)$_{[80+]}$***(1/3)**, J69.0
	3.7 Skin and musculo-skeletal system [80+]	L00-L99.8, M00-M99.9
**IT3b. Frailty, Progressive neurological condition [65-79]**<br>***("Gradually dwindling")***		
	3.8 Dementia and mental disorders [65-79]	A81.0, F01, F03-F07, F09, G30, G31
	3.9 Progressive neurological diseases [65-79]	G10, G12.2, G20, G35, G23.1, G90.3
	3.10 Cerebrovascular diseases [65-79]	(I60-I67)$_{[65-79]}$***(1/3)**, I69
	3.11 Some ill-defined causes [65-79]	R26, R29.6, R53, R54, R55, R63, R64, I46, J96
	3.12 Malnutrition [65-79]	E46
	3.13 Infectious causes / Influenza / Pneumonia [65-79]	A15-A19.9, B90-B90.9, B20-B24<br>(J06, J09-J16, J18)$_{[65-79]}$***(1/3)**<br>(J20-J22)$_{[65-79]}$***(1/3)**<br>J69.0
	3.14 Skin and musculo-skeletal system [65-79]	L00-L99.8, M00-M99.9

IT3c. Frailty, Progressive neurological condition [18-64]

("Gradually dwindling")

	3.15 Dementia and mental disorders [18-64]	A81.0, F01, F03-F07, F09, G30, G31
	3.16 Progressive neurological diseases [18-64]	G10, G12.2, G20, G35, G23.1, G90.3
	3.17 Cerebrovascular diseases [18-64]	I69
	3.18 Some ill-defined causes [18-64]	R26, R29.6, R53, R54, R55, R63, R64, I46, J96
	3.19 Malnutrition [18-64]	E46
	3.20 Infectious causes / Influenza / Pneumonia [18-64]	A15-A19.9, B90-B90.9, B20-B24, J69.0
	3.21 Skin and musculo-skeletal system [18-64]	L00-L99.8, M00-M99.9

4. Various causes

	4.1 Infectious diseases	A00-B99.9<br>*Except: A81.0, A15-A19.9, B18, B20-B24, B90-B90.9*
	4.2 Neoplasms + Diseases of the blood	D01-D89.9
	4.3 Diseases of the nervous system - *except progressive neurological diseases*	G00-H95.9<br>*Except: G10, G12.2, G20, G23.1, G30, G31, G35, G90.3*
	4.4 Symptoms, signs... not elsewhere classified / Ill-defined causes - *except R26, R29.6, R53, R54, R55, R63, R64, and (R96, R98, R99)[80+]*	R00-R99<br>*Except: R26, R29.6, R53, R54, R55, R63, R64, (R96, R98, R99)[80+]*
	4.5 Substance abuse and other mental and behavioral disorders	F10-F98
	4.7 Pregnancy, childbirth, puerperium / Congenital malformation	O00-O998, P00-P96.9, Q00-Q99.9

5. External causes

	5. External causes of morbidity and mortality	V01-Y89.9<br>*<u>Except</u>:(W00, W03-08, W10, W18)[80+]*

[1] One third of the annual deaths with ICD-10 codes (I60-I67) as underlying cause of death (people over 65)

[2] One third of the annual deaths with ICD-10 codes (J06, J09-J16, J18) as underlying cause of death (people over 65)

[3] One third of the annual deaths with ICD-10 codes (J20-J22) as underlying cause of death (people over 65)

The characteristics of the main disease responsible for the death of a patient may be associated with certain health services utilisation patterns in the last months of life. Rather than using every single disease used for underlying causes of death in the International Statistical Classification of Diseases and Related Health Problems ("ICD-10"), we used categories of life-limiting conditions that have been described as having similar courses until death.

The conditions were grouped into three "illness trajectories" ("cancer", "organ failure", "frailty/ progressive neurological conditions"). These trajectories have a shape and an average duration, and they are associated with potential palliative care needs in the people they affect (whether physically, psychologically, socially, existentially or spiritually).

Our goal for this study was to allocate ICD-10 codes used for underlying causes of death in mortality datasets, to these three main categories of conditions ("illness trajectories").

The underlying cause of death is defined as the disease or injury which initiated the chain of events leading directly to death. Every death has just one underlying cause, so is counted only once in figures which are produced on this basis. Deaths are coded in accordance with the ICD-10.

The allocation of ICD-10 codes for underlying causes of death to the three main illness trajectories was completed prior to this study by a panel of international experts in primary care, palliative care, public health and demography, as part of a modified e-Delphi study carried out by two authors of this article (SAM and SM). The panel did not consider contributory causes of death. The list of ICD-10 codes used in this current article has been obtained after two Delphi rounds, and it consists in seven categories (as displayed in Table 1):

1. Cancer
2. Organ failure
3. Frailty/ Progressive neurological conditions [80+]
4. Frailty/ Progressive neurological conditions [65-79]
5. Frailty/ Progressive neurological conditions [18-64]
6. Various causes
7. External causes

The allocation of the total number of deaths to each main category (Scotland, 2016) is featured in Supplementary table 3
