# Supplementary material for: Unscheduled and out-of-hours care for people in their last year of life: a retrospective cohort analysis of national datasets
Source: BMJ Open. 2020 Nov 23;10(11):e041888. doi: 10.1136/bmjopen-2020-041888 (PMC7684800; doi:10.1136/bmjopen-2020-041888)
Supplement: Supplementary data [file bmjopen-2020-041888supp002.pdf]

Supplementary table 2. Price weight allocation for cost analysis.

We allocated a fixed value for each service used based on average values.

Unscheduled Service	Value	Unit	Notes/Sources
***N**: NHS24 CONTACTS*			
NHS 24 Contact	£30.78	per call	Private communication with NHS24.
***O**: OOH PRIMARY CARE CONSULTATIONS*			
GP OOH	£38.00	per consultation	[note 1]
Nurse OOH	£6.80	per consultation	£0.70 per min x 9.72 mins. [note 1]
OOH Average Unspecified "O"	£33.20	per consultation	Weighted average of GP/Nurse consultation applied where GP/Nurse consultations were pooled. Based on ratio observed in data.
***S**: SCOTTISH AMBULANCE SERVICE (SAS)*			
Conveyed	£238.00	per trip	Applied to a S with an E or A in the CUP [note 2]
Not Conveyed	£184.00	per trip	Applied to a S without an E or A in the CUP [note 2]
OOH Average Unspecified "S"	£228.89	per trip	Weighted average of conveyed/not conveyed based on observed ratio used. Applied where A or E presence is unspecified.
***E**: EMERGENCY DEPT*			
Accident and Emergency Visit	£138.00	per visit	[note 3]
***A**: INPATIENT ADMISSIONS*			
Emergency Inpatient Admissions	£1,590.00	Per Admission	Average non-elective inpatient admission (excluding excess bed days) [note 3].

Notes

Two factors made costing emergency inpatient admissions for this cohort difficult. Firstly, the Scottish data does not include detailed costs for patient procedures we took a general population average cost. Secondly, we did not cost according to length of stay. It is possible that people who died from different underlying causes may have required different average lengths of stay. Some of the cohort may have required longer stays than patients from the general population and possibly more expensive or multiple interventions than the general population. Although the precise figures are uncertain, it is undoubtedly true that any hospital admission is an order of magnitude more expensive than any other unscheduled care service.

1. Curtis L et al. Unit Costs of Health & Social Care 2017. Kent: Personal Social Services Research Unit 2017
2. Hobbs R, Bankhead C, Mukhtar T, Stevens S, Perera-Salazar R, Holt T, & Salisbury C. (2016) Clinical workload in UK primary care: a retrospective analysis of 100 million consultations in England, 2007-14, The Lancet, 387, 10035, 2323-2330. http://www.sciencedirect.com/science/article/pii/S0140673616006206.
3. Department of Health, The. Reference Costs 2016-17 Publication. London: The Department of Health 2017

Supplementary table 3
