# Supplementary material for: Unscheduled and out-of-hours care for people in their last year of life: a retrospective cohort analysis of national datasets
Source: BMJ Open. 2020 Nov 23;10(11):e041888. doi: 10.1136/bmjopen-2020-041888 (PMC7684800; doi:10.1136/bmjopen-2020-041888)
Supplement: Supplementary data [file bmjopen-2020-041888supp003.pdf]

Supplementary table 3. Number (%) of patients (18+) in the last year of life in Scotland 2016 by underlying cause of death (N=56,407)

Underlying cause of death	N	%
**Cancer (all types)**	**15,902**	**28.2%**
**Organ failure**	**21,244**	**37.7%**
2.1 Endocrine and metabolic	1,137	
2.2 Circulatory	11,212	
2.3 Respiratory	4,744	
2.4 Liver	1,109	
2.5 Kidney	241	
2.6 Digestive	1,949	
2.7 Other genitourinary	852	
**Frailty 80+**	**10,497**	**18.6%**
3.1 Dementia 80+	4,857	
3.2 Progressive neurological 80+	360	
3.3 Cerebrovascular 80+	2,739	
3.4 Ill-defined 80+	258	
3.4 Malnutrition 80+	3	
3.6 Infections & Pneumonia 80+	1,971	
3.7 Skin & Musculo-skeletal 80+	309	
**Frailty 65-79**	**3,076**	**5.5%**
3.8 Dementia 65-79	932	
3.9 Progressive neurological 65-79	332	
3.10 Cerebrovascular 65-79	1,042	
3.11 Ill-defined 65-79	14	
3.12 Malnutrition 65-79	3	
3.13 Infections & Pneumonia 65-79	572	
3.14 Skin & Musculo-skeletal 65-79	181	
**Frailty 19-64**	**450**	**0.8%**
3.15 Dementia 19-64	55	
3.16 Progressive neurological 19-64	180	
3.17 Cerebrovascular 19-64	50	
3.18 Ill-defined 19-64	16	
3.19 Malnutrition 19-64	2	
3.20 Infections & Pneumonia 19-64	63	
3.21 Skin & Musculo-skeletal 19-64	84	
**Various**	**2,271**	**4.0%**
4.1 Infections	648	
4.2 Neoplasms & blood	456	
4.3 Nervous system	395	
4.4 Symptoms & Signs NEC	235	
4.5 Mental & behavioural	419	
4.7 Pregnancy & congenital	118	
**External**	**2,967**	**5.3%**
