# Supplementary material for: Unscheduled and out-of-hours care for people in their last year of life: a retrospective cohort analysis of national datasets
Source: BMJ Open. 2020 Nov 23;10(11):e041888. doi: 10.1136/bmjopen-2020-041888 (PMC7684800; doi:10.1136/bmjopen-2020-041888)
Supplement: Supplementary data [file bmjopen-2020-041888supp004.pdf]

**Total**	**56,407**	**100.0%**

Supplementary Table 4. Number (%) of contacts with services in each of the 12 months before death by patients (18+) in Scotland 2016 (Month 12 represents the 12th month before death)

	Total		Contact NHS 24		Contact PCOOH		Call to SAS		ED attendance		Emergency admissions	
Months	Number	%	Number	%	Number	%	Number	%	Number	%	Number	%
12	18,385	3.9	4,109	4.3	3,584	3.3	3,602	3.8	3,632	4.5	3,458	3.8
11	19,327	4.1	4,200	4.4	3,687	3.4	3,811	4.0	3,919	4.8	3,710	4.0
10	19,779	4.2	4,374	4.6	3,889	3.6	3,866	4.1	3,942	4.9	3,708	4.0
9	21,136	4.5	4,679	4.9	3,993	3.7	4,056	4.3	4,236	5.2	4,172	4.5
8	22,374	4.7	4,818	5.1	4,306	3.9	4,375	4.6	4,353	5.4	4,522	4.9
7	24,006	5.1	5,164	5.4	4,681	4.3	4,667	4.9	4,625	5.7	4,869	5.3
6	25,542	5.4	5,391	5.7	5,022	4.6	5,102	5.4	4,832	6.0	5,195	5.7
5	28,759	6.1	5,902	6.2	5,652	5.2	5,728	6.0	5,463	6.7	6,014	6.6
4	33,334	7.1	6,579	6.9	6,624	6.1	6,725	7.1	6,250	7.7	7,156	7.8
3	41,634	8.8	7,955	8.3	8,319	7.6	8,616	9.1	7,550	9.3	9,194	10.0
2	56,473	12.0	10,428	10.9	11,567	10.6	11,888	12.5	9,781	12.1	12,809	14.0
1	161,611	34.2	31,751	33.3	47,833	43.8	32,583	34.3	22,488	27.7	26,956	29.4
Total	472,360	100	95,350	100	109,157	100	95,019	100	81,071	100	91,763	100
