# Supplementary material for: Unscheduled and out-of-hours care for people in their last year of life: a retrospective cohort analysis of national datasets
Source: BMJ Open. 2020 Nov 23;10(11):e041888. doi: 10.1136/bmjopen-2020-041888 (PMC7684800; doi:10.1136/bmjopen-2020-041888)
Supplement: Author's manuscript [file bmjopen-2020-041888.draft_revisions.pdf]

BMJ Open is committed to open peer review. As part of this commitment we make the peer review history of every article we publish publicly available.

When an article is published we post the peer reviewers' comments and the authors' responses online. We also post the versions of the paper that were used during peer review. These are the versions that the peer review comments apply to.

The versions of the paper that follow are the versions that were submitted during the peer review process. They are not the versions of record or the final published versions. They should not be cited or distributed as the published version of this manuscript.

BMJ Open is an open access journal and the full, final, typeset and author-corrected version of record of the manuscript is available on our site with no access controls, subscription charges or pay-per-view fees (http://bmjopen.bmj.com).

If you have any questions on BMJ Open's open peer review process please email info.bmjopen@bmj.com

BMJ Open

Unscheduled and out-of-hours care for people in their last year of life: a population analysis in a national health care system

Journal:	*BMJ Open*
Manuscript ID	bmjopen-2020-041888
Article Type:	Original research
Date Submitted by the Author:	19-Jun-2020

Complete List of Authors:	Mason, Bruce; University of Edinburgh<br>Kerssens, Joannes; NHS National Services Scotland, Information Services Division<br>Stoddart, Andrew; University of Edinburgh,<br>Murray, Scott; The University of Edinburgh, Primary Pallaitive Care Research Group, The Usher Institute,<br>Moine, Sébastien ; University of Edinburgh, 3. Primary Palliative Care Research Group, Usher Institute of Population Health Sciences and Informatics; University of Paris 13, Health Education and Practices Laboratory<br>Finucane, Anne; Marie Curie Hospice Edinburgh<br>Boyd, Kirsty; University of Edinburgh, Primary Palliative Care Research Group

Keywords:	PRIMARY CARE, ACCIDENT & EMERGENCY MEDICINE, Adult palliative care < PALLIATIVE CARE

SCHOLARONE™
Manuscripts

I, the Submitting Author has the right to grant and does grant on behalf of all authors of the Work (as defined in the below author licence), an exclusive licence and/or a non-exclusive licence for contributions from authors who are: i) UK Crown employees; ii) where BMJ has agreed a CC-BY licence shall apply, and/or iii) in accordance with the terms applicable for US Federal Government officers or employees acting as part of their official duties; on a worldwide, perpetual, irrevocable, royalty-free basis to BMJ Publishing Group Ltd ("BMJ") its licensees and where the relevant Journal is co-owned by BMJ to the co-owners of the Journal, to publish the Work in this journal and any other BMJ products and to exploit all rights, as set out in our licence.

The Submitting Author accepts and understands that any supply made under these terms is made by BMJ to the Submitting Author unless you are acting as an employee on behalf of your employer or a postgraduate student of an affiliated institution which is paying any applicable article publishing charge ("APC") for Open Access articles. Where the Submitting Author wishes to make the Work available on an Open Access basis (and intends to pay the relevant APC), the terms of reuse of such Open Access shall be governed by a Creative Commons licence – details of these licences and which Creative Commons licence will apply to this Work are set out in our licence referred to above.

Other than as permitted in any relevant BMJ Author's Self Archiving Policies, I confirm this Work has not been accepted for publication elsewhere, is not being considered for publication elsewhere and does not duplicate material already published. I confirm all authors consent to publication of this Work and authorise the granting of this licence.

For peer review only

Unscheduled and out-of-hours care for people in their last year of life: a population analysis in a national health care system

Bruce Mason, Joannes Kerssens, Andrew Stoddart, Scott A. Murray, Sebastien Moine, Anne M. Finucane, Kirsty Boyd

Primary Palliative Care Research Group, Usher Institute, University of Edinburgh, EH8 9AG Scotland, UK Bruce Mason, senior researcher
Electronic Data Research and Innovation Service, Information Services Division, NHS National Services, Little France Road, Edinburgh, EH16 4UX Scotland, UK Joannes Kerssens, principal information analyst and statistician

Edinburgh Clinical Trials Unit, Usher Institute, University of Edinburgh, Scotland, EH8 9AG UK Andrew Stoddart, senior health economist[3]

Primary Palliative Care Research Group, Usher Institute, University of Edinburgh, Scotland, EH8 9AG UK Scott A. Murray, emeritus professor[1]

Health Education and Practices Laboratory, University of Paris 13, Bobigny, France. Sebastien Moine, senior visiting fellow at Usher Institute, Edinburgh, UK

Marie Curie Hospice, 45 Frogston Road West, Edinburgh, EH10 7DR
Edinburgh, Scotland, UK Anne M. Finucane, research lead

Primary Palliative Care Research Group, Usher Institute, University of Edinburgh, Scotland, EH8 9AG UK Kirsty Boyd, reader

Correspondence to Scott A Murray, Primary Palliative Care Research Group, Usher Institute, University of Edinburgh, Scotland, EH8 9AG UK Scott.Murray@ed.ac.uk

Key words: Out-of-hours care, unscheduled care, routine data, primary care, care pathway

For peer review only - http://bmjopen.bmj.com/site/about/guidelines.xhtml

Summary:

Objectives To analyse patterns of use and costs of unscheduled NHS services for people in the last year of life.

Design Analysis of national datasets with application of standard UK costings

Participants and setting All people who died in Scotland in 2016 aged 18 or more (n= 56,407)

Main outcome measures Frequency of use of the five unscheduled NHS services in the last 12 months of life by underlying cause of death, deprivation status, place of residence, rurality and evidence of identification for palliative care; continuous unscheduled care pathways (CUPs) followed by patients during each care episode; total NHS and per-patient costs.

Principal findings 53,509 (94.9%) of patients had at least one contact with an unscheduled care service during their last year of life: a total of 472,360 contacts, with 34.2% in the last month of life. People with various underlying causes of death, deprivation levels, and place of residence used unscheduled care differently which impacted on per-person costs. By linking patient contacts during each episode of care, we identified 206,841 continuous unscheduled pathways (CUPs); with 133,980 (64.8%) starting out-of-hours. Evidence of identification for palliative care was only recorded in the primary care out-of-hours dataset. People with cancer were more likely than others to contact the telephone advice line (NHS24) or primary care out-of-hours (PCOOH) and have emergency admissions. People with organ failure most frequently contacted the ambulance service. The cost of providing services in the community was estimated at 3.9 % of total unscheduled costs despite handling most out-of-hours calls.

Conclusions Use of unscheduled care by people in their last year of life is more frequent and involves more services than reported previously. Different underlying causes of death and social factors impacted on initial access and subsequent pathways of care. Managing more unscheduled care episodes in the community has the potential to reduce admissions and overall costs.

For peer review only - http://bmjopen.bmj.com/site/about/guidelines.xhtml

Article summary

Strengths and limitations of this study

- Most studies of unscheduled care have focussed on individual services and specific diseases
- We linked all five unscheduled NHS services into Continuous Unscheduled Pathways (CUPs) and identified common patterns of unscheduled care for people in their last year of life.
- This innovative population based method provided a broad understanding of how different illnesses, and demographic factors affected use of unscheduled care throughout Scotland
- Only one of the five datasets contained information that could indicate whether a person was identified for palliative care.
- Costs were calculated using weighted averages and were therefore approximate.

Introduction

The rising demand for unscheduled care is a major burden and causes pressure on healthcare systems internationally, both in and out-of-hours. Unscheduled care is unplanned and demand-led and includes in the UK national health service five services: telephone advice, primary care services, ambulance services, emergency department and acute hospital care. Emergency department targets were unmet throughout the UK prior to the impact of Covid-19. Unplanned hospital admissions in the UK increased by 28% from 2010-2019, while elective admissions rose by 25% during the same time period. [1] Contributory factors include an aging population with multiple health conditions, public expectations, instructions to seek urgent care for suspected strokes or heart attacks, and less support in the community at weekends and overnight. [2] Much unscheduled care is used by people in their last year of life who are known to have significant health-related suffering and unmet palliative care needs. [3]

For peer review only

For peer review only - http://bmjopen.bmj.com/site/about/guidelines.xhtml

Early integration of a palliative approach as a component of chronic disease management is strongly recommended for people with advanced illnesses in all care settings. [4,5] Palliative care is a core component of universal health coverage and its people-centered ethos and focus on quality of life and death should be considered at every opportunity. [6] Services should be designed to respond to the typical trajectories of declining health of patients with all progressive illnesses, including social and psychological factors. [7, 8] Palliative care includes proactive care planning which reduces burdensome interventions of low benefit, and helps avoid some unwarranted hospital admissions. However, palliative care integration into unscheduled care services is particularly challenging. [9]

National datasets can be used to monitor and improve care. They have underutilised potential to improve end-of-life care. [10] In Scotland, death registry data, and activity data from hospitals, the ambulance service and out-of-hours primary care services are collected routinely. These national datasets contain a unique identifier—the Community Health Index (CHI). We set out to link these data to analyse unscheduled care services for people with different underlying causes of death and demographic factors, and to estimate costings for each service.

Methods

We linked three datasets. The *National Records of Scotland (NRS) deaths dataset* was used to identify all adults (aged 18+) who died in Scotland in 2016. From this dataset we extracted underlying cause of death and usual place of residence. From the *Hospital Admissions dataset*, we extracted all unscheduled hospital activity for the last 12 months life by using the Scottish Morbidity Record (SMR01) for the same cohort. Thirdly, we extracted data from the *Scottish Unscheduled Care Datamart* (UCD) covering four unscheduled care services: the telephone advice line (NHS24), primary care out-of-hours (PCOOH), the Scottish ambulance service, and emergency department attendances. [11] The UCD does not include in-hours unscheduled primary care.

For peer review only - http://bmjopen.bmj.com/site/about/guidelines.xhtml

The ICD codes (v 2010) for underlying cause of death extracted from the NRS dataset were classified into five groups: cancer, organ failure, frailty/progressive neurological conditions, various other causes, and external causes.[12] People in the first three groups were considered potentially to have had palliative care needs during their last year of life. Details of this coding allocation had been agreed previously by an expert international panel. [13] (See online supplementary table 1)

Postcodes of usual place of residence were extracted from the NRS dataset. We used the Scottish Index of Multiple Deprivation (SIMD 2016) to infer quintiles of deprivation. [14] Rurality was based on the Scottish Government Urban-Rural Classification as applied to the postcodes. [15]

Whether the person had been identified for palliative or anticipatory care could only be derived from data included in the primary care out-of-hours dataset. That dataset contained palliative care Read codes which clinicians can apply to such consultations and also recorded whether the patient had a Key Information Summary (KIS). [16] A KIS is created by primary care teams for patients receiving anticipatory/advance care planning. If one or both of these indicators was present, we categorised the person as 'identified for palliative care'.

In order to understand how multiple services were accessed during a single healthcare episode, we used Continuous Unscheduled Pathways (CUPs) as defined in the Scottish unscheduled care datamart. A CUP is a linked set of contacts with one or more unscheduled care services where each new contact starts within 24 hours of the previous one. [11] Each CUP represents a single patient journey through one or more unscheduled NHS services. The frequencies of the different types of CUPs were tabulated to identify key patterns. We categorised a CUP as 'out-of-hours' if it started during the out-of-hours period: defined as weekends, public holidays and weeknights from 6pm to 8am. There is no limit to the duration of each CUP so a CUP could start out-of-hours but end during the in-hours period and vice-versa. Therefore, only the start date and time of the CUP was used to categorise it as "in-hours" or "out-of-hours".

For peer review only - http://bmjopen.bmj.com/site/about/guidelines.xhtml

In the Unscheduled Care Dataset, each service component of a CUP is assigned a code letter:

- N=NHS 24
- O= Primary Care Out of Hours
- S=Scottish Ambulance Service
- E=Emergency Department
- A= Acute hospital admission

Linking these codes in chronological order gives the pathway its name: 'NSE' represents a call to NHS 24 (N), followed by an ambulance service contact (S), then an emergency department attendance (E).

Statistical analysis: Most analyses were descriptive with means and frequency tables, and compared people with or without contacts with unscheduled care services using chi-squared tests for categorical variables. Multiple logistic regression models analysed multivariate associations between predictor variables (gender, age, marital status, deprivation quintile, cause of death, urban/rural classification and place of residence) and the odds of using a service. All analyses were conducted within the Scottish National Safe Haven by a senior analyst (JK) under Public Benefit following a Privacy Panel (PBPP) review and approval.

Cost estimations: Standard UK price weighting methodology was applied to estimate the costs of each unscheduled service: see online supplementary table 2. We did not attribute exact pricing to different forms of inpatient admission or account for differences due to patient demographics hence mean population values were applied. These costs are included as broad indicators of differences in scale and should not be interpreted as exact data.

Patient and Public Involvement: Representatives from Marie Curie Expert Voices Scotland and a Royal College of General Practitioners Scottish patient group joined the steering group and contributed public-patient perspectives from their own groups throughout the project. Key

For peer review only - http://bmjopen.bmj.com/site/about/guidelines.xhtml

For peer review only

stakeholders from the unscheduled services and patient group members advised the research team on parameters for analysis, choice of analyses, and data interpretation. To understand decision-making and experiences of service users, we conducted focus groups and interviews with patients with advanced illnesses and carers who had used unscheduled services and with bereaved carers. These data will be reported elsewhere. A lay summary will be sent to study participants and the patient and public involvement groups involved in this study.

The study was funded by Marie Curie grant MCRGS-07-16-37 and the Chief Scientist Office Scotland.

Permission to access and link data from the Scottish Public Benefit and Privacy Panel for health and social care, ref 1516-0483 and favourable ethical review from SE Scotland Research Ethics committee 2 reference 17/SS/0127 were gained. Data sharing: More information may be available from the corresponding author.

Results

1 Underlying causes of death in the cohort

We extracted records for 56,407 people who died in Scotland in 2016 and linked records covering the last 12 months of life for each individual in this cohort. The number of people in each diagnostic group was as follows: cancer 28.2%, organ failure 37.7%, frailty/progressive neurological conditions 24.9%, other diseases / various causes 4.0%, and external causes 5.3%. (see online supplementary table 3)

2 Use of NHS unscheduled care services

The cohort had 472,360 unscheduled care service contacts; 56,407 people (94.9% of the cohort) had at least one contact. Table 1 shows the distribution of unscheduled service use: 50.4% had 6 or more contacts, and the 5.4% who had 20 or more contacts accounted for 21.5% of all contacts. All

For peer review only - http://bmjopen.bmj.com/site/about/guidelines.xhtml

unscheduled care service were increasingly used as death approached, with 34.2% occurring in the last month of life (see online supplementary table 3). During that final month, there was a disproportionate rise in primary care out-of-hours workload.

Table 2 displays the number and percentages of people who contacted the five separate unscheduled care services during the last 12 months of life by cause of death and social factors. Place of residence at death had two categories: those living in a private residence versus people living in any institution. The latter were primarily care homes, but also included prisons and hostels. Due to the large sample size, all differences in Table 4 were statistically significant (except contacts with NHS 24 by deprivation). We now highlight the clinically important findings for each service.

24-hour Telephone Advice Service (NHS24)

More people dying with frailty (66.0%) or cancer (63.4%) contacted this service than those dying with organ failure (56.0%). People living in institutions (or commonly the staff caring for them) were more likely to contact NHS24 than those living at home: 68.8% v 58.0%. People from all deprivation quintiles were about as likely to access NHS24. People who lived in urban areas contacted NHS24 more than those from remote areas.

Primary Care OOH (PCOOH)

Service use was similar to NHS24, as more people dying with cancer (61.5%) or frailty (61.7%) contacted this service compared to those with organ failure (45.6%). People living in an institution were substantially more likely to access this service than those in a private residence: 69.3% v 50.7%. People living in the most deprived quintile were least likely to access PCOOH (46.6%).

Scottish Ambulance Service

By contrast, people who died from organ failure used this service (78.9%) more than those with cancer (72.8%) or frailty (67.8%). People living at home were much more likely to phone the ambulance

For peer review only - http://bmjopen.bmj.com/site/about/guidelines.xhtml

service much more than those in an institution (77.2% v 55.7%). People in the most deprived quintile accessed help from the ambulance service the most (77.2%).

Emergency Department

People who died from organ failure used this service (65.2%) more than those with cancer or frailty as they did with the ambulance service. Likewise, people living in institutions were less likely to visit and emergency department than those living in the community (46.7% v 65.6%). People in the most deprived quintile were more likely to attend an emergency department (67.3%).

Acute Hospital Admission

More people who died from cancer had at least one acute hospital admission (88.3%) than those with organ failure (73.2%) or frailty (66.4%). People living at home were much more likely to be admitted to hospital (79.3%) than those living in an institution (50.6%). In general, people in more deprived quintiles were admitted more often.

Patterns of use across unscheduled care services

Logistic regression modelling included the clinical, socioeconomic and location variables from Table 4 plus age, sex and marital status. (Table 3) The odds ratio (OR) for contacts with NHS24 was higher for women than men (adjusted OR 1.17, 95% CI = 1.12-1.21). People aged 65-84 had higher odds ratios for acute hospital admission (adjusted OR 1.68, 95% CI = 1.58-1.78). Older people aged 85 or over had slightly lower ratios, likely due to care home residence (adjusted OR 1.67, 95% CI = 1.56-1.79). Comparing the three main groups of causes of death, the odds of people with cancer having a PCOOH contact in their last year of life was much greater than for people dying with organ failure (adjusted OR 2.08, 95% CI = 1.99-2.17). After controlling for demographic and location variables, people with cancer had a higher risk of an acute admission compared to people with frailty (adjusted OR 2.77, 95% CI = 2.60-2.95). Overall, people living at home used more unscheduled care services than those in

For peer review only - http://bmjopen.bmj.com/site/about/guidelines.xhtml

For peer review only

institutions. This was particularly the case for acute hospital admissions (adjusted OR 3.39, 95% CI = 3.21-3.57).

People in the most deprived quintile tended to use the three unscheduled care services that are not community based more than those in the least deprived quintile. This was especially so for ambulance services (adjusted OR 1.42, 95% CI = 1.33-1.51). Those living in urban areas used more of all the services except PCOOH than people from rural areas probably due to service organisation in remote, rural areas in Scotland.

3. Use of Continuous Unscheduled Pathways (CUPs)

Linking the serial use of the unscheduled service contacts into CUPs enabled us to understand common orders and patterns of service use. Figure 1 displays the first and last points of contact for all unscheduled care pathways both day and night. NHS24 and the ambulance service were the most common initial access points. The most common end points were PCOOH and hospital admission. Over half of ambulance calls led to an emergency admission (60.0%). Conversely, 50.6% of initial calls to NHS24 were dealt with in the community by PCOOH.

Differences in frequencies and types of CUP by when they started

We differentiated between CUPs that started out-of-hours and in-hours as we wished to look at implications for improving out-of-hours care as well as unscheduled care in general. We identified 206,841 continuous unscheduled pathways, of which 133,980 (64.8%) started out-of-hours, 28.1% in-hours, and 7.1% unknown (mostly due to lack of a time-stamp on emergency admissions). Contacts with NHS24 and PCOOH were much more frequent in in out-of-hours CUPS. The percentages of contacts with each service that occurred during out-of-hours CUPs compared with in-hours CUPs were: NHS24 93.1%, PCOOH 94.7%, ambulance service 37.7%, and emergency department 44.4%.

For peer review only - http://bmjopen.bmj.com/site/about/guidelines.xhtml

Table 4 shows the 20 most common CUPs that started out-of-hours and the 20 most common that started in-hours to allow an understanding of how patients typically move through the services night and day. Only 16.7% of out-of-hours CUPs started with an ambulance call while 73.2% started with an NHS24 or PCOOH contact. Similarly, most out-of-hours CUPs ended in primary care: 9.6% with telephone advice from NHS24 and 46.7% with PCOOH. Much fewer out-of-hours CUPs resulted in a hospital admission (27.5%) or emergency department attendance (8.2%). In contrast, the six most common CUPs which started in-hours comprised episodes consisting of ambulance calls, emergency department visits and acute hospital admissions, and these accounted for 74.2% of all CUPs which started in-hours. GP in-hours care was not included in the UCD.

4. Costs

The mean number of contacts, per patient costs and total NHS costs for the five unscheduled care services in the last year of life are listed by underlying causes of death and deprivation status in detailed in table 5. The group of people dying with organ failure had the highest total NHS costs because they accounted for the greatest number of deaths overall and due to their high use of secondary care services. Those with frailty incurred the least unscheduled NHS care costs, being managed more in the community. The total cost of unscheduled care to the NHS in Scotland for people in their last year of life was nearly £190 million, of which only 3.9% was for provision of primary care services.

The total mean per patient costs of unscheduled care in the last year of life for those with cancer, organ failure or frailty were £4,083, £3,429, and £2,654 respectively. Unscheduled per patient costs for people in the most deprived quintile were 18.8% higher than those from the least deprived but their PCOOH costs per capita were 30.6% lower.

Discussion

Principal findings

For peer review only - http://bmjopen.bmj.com/site/about/guidelines.xhtml

We found that 94.9% of people had unscheduled care contacts during their last year of life. They had a median of five contacts, with 5.1% making 20 or more contacts, and 34.2% of all contacts occurring during the final month. We identified three groups of patients by underlying cause of death with clinically and statistically significant different patterns of unscheduled care service use. People with cancer had more unscheduled admissions than people with non-cancer diagnoses and the highest per patient costs. People who died with frailty, especially those who lived in institutions, were most likely to have unscheduled care that was managed fully in the community People with organ failure made the most use of ambulance services and accounted for the greatest number of acute hospital admissions overall due to there being more deaths in this group. People from the most deprived quintile used significantly less PCOOH than those from the least deprived but they accessed the other four unscheduled services more. The total cost of service use by people in the most deprived quintile was almost double that of those in the least deprived, due to the greater numbers dying in this group and their higher use of secondary care services. NHS24 and PCOOH services together accounted for less than 4% of total NHS unscheduled care costs.

Strengths and limitations of the study

Strengths This innovative approach to studying population use of interconnected unscheduled care services provided a broad understanding of how different illnesses, and demographic factors affected use of unscheduled care. Linking all five unscheduled NHS services together into patient pathways (CUPs) allowed common patterns of unscheduled care to be identified and quantified. Studying the unscheduled care service pathways of people in their last year of life throughout Scotland enabled us to analyse the unscheduled care provided for a population which consists of rising numbers of people with unidentified palliative care needs.[3] Understanding the perspectives and choices made by people and their families seeking unscheduled care is equally important and was part of our overall study (to be reported elsewhere).

For peer review only

For peer review only - http://bmjopen.bmj.com/site/about/guidelines.xhtml

Limitations Lack of national data for in-hours, unscheduled primary care was limiting. Some CUPs recorded as out-of-hours may have started with an undocumented, urgent primary care contact in-hours. Costs were calculated using weighted averages and were therefore approximate. Only one dataset (PCOOH) contained information that could be used to indicate whether a person had been identified for anticipatory care planning or palliative care. The lack of a routinely collected palliative care code or record of the Key Information Summary care plan being accessed in the other four nationally recorded datasets meant it was impossible to estimate the full extent of proactive care planning and palliative care provision in the other unscheduled services.

Comparison with other studies

Most studies of unscheduled care have focussed on individual services and specific diseases, notably emergency departments and patients with cancer, and have suggested that only 30-35% of people with cancer use unscheduled care in the last year of life. [17, 18] However, a recent study in one Scottish region reported that 78% of people dying from cancer had unscheduled care provided by emergency departments and/or PCOOH in their last year of life [19] Our findings, integrating all five NHS unscheduled care services, found that 94.9% of people who died in Scotland received unscheduled care in their last year of life. By using population data along with specific service use data, we highlighted the extent and diversity of unscheduled care pathways. Our logistic regression models identified differences in unscheduled care by deprivation quintile for each of the three main illness groups, not just for the population with cancer [19].

Meaning of the study and implications for clinicians and policymakers

Many more people seek unscheduled and out-of-hours care in their last year of life than recognised previously. There were common patterns associated with different underlying illnesses, deprivation status and place of residence. Knowledge of how these groups of patients respond to urgent care needs may help community and hospital services find ways to respond more effectively and

For peer review only - http://bmjopen.bmj.com/site/about/guidelines.xhtml

potentially reduce demand for costly services. Primary care teams, social care managers and hospital teams can identify frequent or unusual contacts with unscheduled care and use these to trigger new or updated care planning. Such care planning communicated to unscheduled care services routinely via primary care managed electronic care co-ordination systems has been linked with fewer hospital admissions and deaths in Scotland. [16, 20] In London, the "Coordinate my Care" system uploads and shares urgent care plans entered by primary care, ambulance and hospital services thereby reducing hospital admissions, and electronic care planning systems are evolving in other parts of England. [17, 21, 22] The UK ReSPECT process partners with patients and families to make emergency treatment and care plans that can help guide unscheduled care and reduce unwarranted admissions. [23] Key aspects are early identification of people at risk of deteriorating health, proactive care planning, and a readily accessible electronic care coordination system that can be read and updated by any professional responsible for a person's care.

Improving the ability of unscheduled primary care services to manage people in the community is likely to be highly cost-effective as well as supporting people's choice to remain at home towards the end of life. Emergency departments are already employing more primary care clinicians to enhance pre-hospital triage and ambulance crews are providing more care at home, where appropriate, instead of transferring patients to hospital. [24] Interventions in the community by NHS 24, PCOOH and ambulance services have potential to provide high- value low-cost care.

Further research

Unscheduled care of the whole population merits ongoing research using population-level data, encompassing all community and hospital settings both in-hours and out-of-hours. Improving the scope and quality of data collected routinely can facilitate research into the needs of people who are high service users and stand to benefit from better coordinated care. [5, 6, 29] Specifically, research to understand specific differences in care pathways and service use is important, such as why people with cancer have more urgent hospital admissions than others and why people with organ failure

For peer review only - http://bmjopen.bmj.com/site/about/guidelines.xhtml

call the ambulance service relatively frequently. Our data also provides a baseline that can be used in studies to evaluate changes in the use of unscheduled services during the coronavirus pandemic, when the demand for hospital-based unscheduled care dipped sharply.

Interventions to integrate palliative care into each of the five out-of-hours services, as well as care coordination throughout the unscheduled pathways are recommended. [25] An evaluation of telephone advice services including emergency social care, community nurse telephone support such as the Gold Line, specialist palliative care support lines, and from charities offering help for people with specific illnesses is indicated to scope provision of unscheduled care in the community further. [26] Review of specialist palliative care out-of-hours, which has not been included in this study, and interventions to co-ordinate care between settings at the end of life are also needed. [21]

Conclusions

The extent of unscheduled care delivered to people in their last year of life is significantly greater and more varied than reported previously. People with diverse urgent care needs are accessing these services at high levels, particularly in their final month of life. More should be done to take account of underlying illness trajectories and social determinants of health, including better public understanding of how to access the right care in timely and effective ways. Systematic approaches to care planning combined with effective recording and sharing of key information is vital and should be recorded in routine datasets.

Acknowledgments:

The authors acknowledge the support of the eDRIS team (National Services Scotland) for their involvement of obtaining approvals, provisioning and data linkage. We also acknowledge advice from the wider project steering group: Marilyn Kendall, senior researcher; Sheonad Laidlaw, general practitioner; Emma Carduff, honorary research fellow research lead; Erna Haraldsdottir, senior lecturer; Sir Lewis Ritchie, Scottish Government advisor; Sian Tucker, NHS24 national lead; Marie Fallon, professor of palliative medicine; Jeremy Keen, consultant in palliative medicine; Stella Macpherson, patient and public involvement representative; Nicole Brun, project administrator. We also thank Bruce Guthrie and Sarah Mills for commenting on a draft of this paper.

For peer review only - http://bmjopen.bmj.com/site/about/guidelines.xhtml

Contributor and guarantor information: BM, SAM, SM, AF, AS and KB designed the study (along with members of the steering group acknowledged above), JK and AS linked and analysed the data, SM allocated illness trajectories to all decedents, and AF conducted literature reviews. All contributors discussed the findings and contributed to the writing of the drafts and the final paper. The contributors met with the steering regularly to design and guide the overall study and to integrate patient and carer perspective. SAM is the guarantor. The corresponding author attests that all listed authors meet authorship criteria and that no others meeting the criteria have been omitted.

Copyright /license for publication: The Corresponding Author has the right to grant on behalf of all authors and does grant on behalf of all authors, a worldwide licence to the Publishers and its licensees in perpetuity, in all forms, formats and media (whether known now or created in the future), to i) publish, reproduce, distribute, display and store the Contribution, ii) translate the Contribution into other languages, create adaptations, reprints, include within collections and create summaries, extracts and/or, abstracts of the Contribution, iii) create any other derivative work(s) based on the Contribution, iv) to exploit all subsidiary rights in the Contribution, v) the inclusion of electronic links from the Contribution to third party material where-ever it may be located; and, vi) licence any third party to do any or all of the above."

Funding: Marie Curie grant MCRGS-07-16-37 and the Chief Scientist Office Scotland

Competing interests: All authors have completed the ICMJE uniform disclosure form at www.icmje.org/coi_disclosure.pdf and declare: no support from any additional organisations for the submitted work; no financial relationships with any organisations that might have an interest in the submitted work in the previous three years; no other relationships or activities that could appear to have influenced the submitted work.

Approvals: Permission to access and link data from the Scottish Public Benefit and Privacy Panel for health and social care, ref 1516-0483 and favourable ethical review from SE Scotland Research Ethics committee 2 reference 17/SS/0127 were gained.

Data sharing: More information may be available from the corresponding author.

Dissemination declaration: We plan to disseminate the results to patient organisations

References

1. NHS Digital. Hospital admitted patient care and adult critical care activity 2018-19. 19 Sep 2019. https://files.digital.nhs.uk/F2/E70669/hosp-epis-stat-admi-summ-rep-2018-19-rep.pdf.
2. Bone AE, Gomes B, Etkind SN, et al. What is the impact of population ageing on the future provision of end-of-life care? Population-based projections of place of death. *Palliat Med.* 2018; 32: 329-336.
3. Murtagh, F.E., et al., How many people need palliative care? A study developing and comparing methods for population-based estimates. *Palliat Med*, 2014. **28**(1): p. 49-58.
4. Institute of Medicine of the National Academies. *Dying in America: Improving Quality and Honoring Individual Preferences Near the End of Life*. The National Academies Press; Washington, DC: 2015.

For peer review only - http://bmjopen.bmj.com/site/about/guidelines.xhtml

5. World Health Assembly. Strengthening of palliative care as a component of comprehensive care throughout the life course. Resolution WHA67.19 adopted by the sixty-seventh World Health Assembly. Geneva: WHO, 2014.
6. World Health Organization and United Nations International Children's Fund. Declaration of Astana. Global Conference on Primary Health Care. Astana, Kazakhstan, October 25–26, 2018. Geneva and New York: WHO and UNICEF, 2018.
7. Murray SA, Kendall M, Boyd K, Sheikh A. Illness trajectories and palliative care. *BMJ*. 2005;330(7498):1007-11.
8. Murray SA, Kendall M, Mitchell G, Moine S, Amblàs-Novellas J, Boyd K. Palliative care from diagnosis to death. *BMJ*. 2017;356.
9. Henson, L. A., Higginson, I. J. and Gao, W. 'What factors influence emergency department visits by patients with cancer at the end of life? Analysis of a 124,030 patient cohort', *Palliat Med*, 2018. 32(2), pp. 426–438.
10. Davies, J.M., et al., Using routine data to improve palliative and end of life care. BMJ Support *Palliat Care*, 2016. 6(3): p. 257-62.
11. Public Health Scotland, Patient Pathways: Emergency Care. Accessed: 24/4/2020 https://www.isdscotland.org/Health-Topics/Emergency-Care/Patient-Pathways/
12. World Health Organisation. International Statistical Classification of diseases and related health problems version 10 (ICD 10). https://www.who.int/classifications/icd/ICD10Volume2_en_2010.pdf
13. Moine, S. How to Better Visualise the Distribution of Expected Annual Deaths in a "Standard" French GP Surgery? Rebuilding the Three Main Illness Trajectories through a Modified Delphi Study. Abstract FC51. *Palliat Med* 2016. 30: NP1–NP401.
14. Scottish Government. The Scottish Index of Multiple Deprivation. Accessed: 24/4/2020 https://www2.gov.scot/Topics/Statistics/SIMD
15. Scottish Government. Scottish Government Urban Rural Classification 2016. (29 Mar 2018). Accessed 24/4/2020. https://www.gov.scot/publications/scottish-government-urban-rural-classification-2016/pages/2/
16. Tapsfield J, Hall C, Lunan C, et al. Many people in Scotland now benefit from anticipatory care before they die: an after death analysis and interviews with general practitioners. *BMJ Support Palliat Care*. 2019;9(4):e28. doi:10.1136/bmjspcare-2015-001014.
17. Henson L, Higginson I, Gao W, BuildCARE. What factors influence emergency department visits by patients with cancer at the end of life? Analysis of a 124,030 patient cohort. *J Palliat Med* 2018. 32(2):426–438.
18. Alsirafy SA, Raheem AA, Al-Zahrani AS, et al. Emergency department visits at the end of life of patients with terminal cancer: pattern, causes, and avoidability. *Am J Hosp Palliat Care* 2016. 33(7):658–662.
19. Mills S, Buchanan D, Guthrie B, et al. Factors affecting use of unscheduled care for people with advanced cancer: a retrospective cohort study in Scotland *BJGP* 2019; 69 (689): e860-e868. DOI: https://doi.org/10.3399/bjgp19X706637
20. Finucane AM, Davydaitis D, Horseman Z, Carduff E, et al. Electronic care coordination systems for people with advanced progressive illness: a mixed-methods evaluation in Scottish primary care. *Br J Gen Pract*. 2020. doi: 10.3399/bjgp19X707117
21. Smith, C., et al., Coordinate My Care: a clinical service that coordinates care, giving patients choice and improving quality of life. BMJ Support & Palliat Care, 2012. 2(4): p. 301-307.

For peer review only - http://bmjopen.bmj.com/site/about/guidelines.xhtml

22. Leniz, J., Weil, A., Higginson, I. J., & Sleeman, K. E. Electronic palliative care coordination systems (EPaCCS): a systematic review. *BMJ Support & Palliat Care*, 2020. 10(1), 68 LP – 78. https://doi.org/10.1136/bmjspcare-2018-001689
23. Hawkes CA, Fritz Z, Deas G, et al. Development of the Recommended Summary Plan for Emergency Care and Treatment (ReSPECT), (2020). *Resuscitation*, 148: 98-107, https://doi.org/10.1016/j.resuscitation.2020.01.003.
24. Cooper, A., Carson-Stevens, A., Hughes, T., & Edwards, A. Is streaming patients in emergency departments to primary care services effective and safe? *BMJ*, 2020;368, m462. https://doi.org/10.1136/bmj.m462
25. Knaul, Felicia M et al. The Lancet Commission on Palliative Care and Pain Relief—findings, recommendations, and future directions, Volume 6, Special Issue, S5-S6, 1st March, 2018. https://doi.org/10.1016/S2214-109X(18)30082-2
26. Best S, Tate T, Noble B, et al. Research Priority Setting in Palliative and End of Life Care: The James Lind Approach Consulting Patients. *BMJ Support Palliat Care*. 2015;5(1):102 LP - 102. doi:10.1136/bmjspcare-2014-000838.

For peer review only

For peer review only - http://bmjopen.bmj.com/site/about/guidelines.xhtml

Table 1. Number (percent and cumulative percent) of patients (18+) in the last year of life in Scotland 2016 by number of contacts with unscheduled care services (N=56,407)

N of contacts with services	N patients	% patients	Cumulative%
0	2,898	5.1	5.1
1	3,983	7.1	12.2
2	5,459	9.7	21.9
3	5,795	10.3	32.2
4	5,205	9.2	41.4
5	4,650	8.2	49.6
6	4,185	7.4	57.0
7	3,589	6.4	63.4
8	3,090	5.5	68.9
9	2,715	4.8	73.7
10	2,300	4.1	77.8
11	1,901	3.4	81.1
12	1,646	2.9	84.1
13	1,318	2.3	86.4
14	1,110	2.0	88.4
15	967	1.7	90.1
16	809	1.4	91.5
17	679	1.2	92.7
18	570	1.0	93.7
19	520	0.9	94.6
20+	3,018	5.4	100
Total	56,407	100	

For peer review only

For peer review only - http://bmjopen.bmj.com/site/about/guidelines.xhtml

For peer review only

For peer review only - http://bmjopen.bmj.com/site/about/guidelines.xhtml

Table 2. Number (and percentage) of people with contacts with NHS24, PCOOH, SAS calls, ED attendances and Emergency admissions for patients (18+) in the last year of life in Scotland 2016 by underlying cause of death, deprivation, place of residence, urban/rural classification (N=56,407)

		Contact NHS 24		Contact PCOOH		Call to SAS		ED attendance		Emergency admissions	
	All persons	% with contact	# with contact	% with contact	# with contact	% with calls	# with calls	% with attendance	# with attendance	% with admissions	# with admissions
**Total***	56,407	59.7	33,656	53.5	30,161	73.9	41,678	62.7	35,383	74.9	42,253
**Cause of death**											
Cancer	15,902	63.4	10,074	61.5	9,783	72.8	11,569	62.0	9,857	88.3	14,039
Organ failure	21,244	56.0	11,888	45.6	9,678	78.9	16,770	65.2	13,851	73.2	15,559
Frailty	14,023	66.0	9,258	61.7	8,654	67.8	9,509	58.9	8,262	66.4	9,314
Various	2,271	56.9	1,292	48.9	1,111	76.4	1,735	64.9	1,474	74.1	1,683
External	2,967	38.5	1,142	31.5	935	70.6	2,095	65.3	1,937	55.9	1,659
**Deprivation**											
Most	13,537	59.8	8,093	46.6	6,307	77.2	10,449	67.3	9,111	76.4	10,347
2	12,812	59.8	7,665	52.5	6,720	76.9	9,850	66.5	8,517	77.2	9,892
3	11,747	59.2	6,959	55.8	6,558	72.3	8,498	60.0	7,050	73.9	8,680
4	9,840	59.8	5,883	58.8	5,790	71.5	7,032	58.7	5,775	72.9	7,177
Least	8,315	60.2	5,002	56.9	4,731	69.4	5,774	58.4	4,853	72.7	6,044
**Place of residence**											
Home/Private	47,813	58.0	27,751	50.7	24,221	77.2	36,891	65.6	31,366	79.3	37,907
Institution	8,558	68.8	5,891	69.3	5,928	55.7	4,770	46.7	4,000	50.6	4,328
**Urban/rural**											
Large Urban Areas	18,750	60.6	11,359	49.0	9,183	74.9	14,053	63.3	11,868	75.4	14,141
Other Urban Areas	20,372	61.4	12,505	53.9	10,986	75.0	15,270	66.8	13,612	75.6	15,407
Accessible Small Towns	5,244	57.7	3,026	55.9	2,934	74.1	3,884	62.9	3,297	74.9	3,926
Remote Small Towns	2,367	56.3	1,332	59.3	1,404	71.6	1,694	54.8	1,296	72.7	1,721
Accessible Rural	5,989	59.6	3,570	58.9	3,526	71.9	4,304	59.1	3,542	74.3	4,447
Remote Rural	3,529	51.3	1,810	58.7	2,073	68.0	2,398	47.9	1,691	70.8	2,498

* Categories don't add up to "Total" because the categories "Other/Unknown" are not displayed.

For peer review only - http://bmjopen.bmj.com/site/about/guidelines.xhtml

Table 3. Adjusted Odds ratios (OR) and 95% Confidence intervals for the probability of contacting NHS24, PCOOH, calling SAS, attending ED and Emergency hospital admissions (HA) for patients (18+) in their last year of their life (N=56,112)*

	NHS24	PCOOH	SAS	ED	HA
	OR (95%CI)	OR (95%CI)	OR (95%CI)	OR (95%CI)	OR (95%CI)
**Sex**					
Male	1	1	1.08 (1.04-1.13)	1.07 (1.03-1.11)	1
Female	1.17 (1.12-1.21)	1.14 (1.10-1.19)	1	1	1.06 (1.02-1.11)
**Age categories**					
Age18_64					
Age65_84	1.22 (1.16-1.28)	1.18 (1.12-1.24)	1.42 (1.34-1.50)	1.17 (1.11-1.23)	1.68 (1.58-1.78)
Age85plus	1.61 (1.51-1.71)	1.61 (1.52-1.71)	1.43 (1.34-1.53)	1.12 (1.05-1.19)	1.67 (1.56-1.79)
**Marital status**					
Single	1	1	1	1	1
Married	1.20 (1.14-1.27)	1.24 (1.17-1.31)	1.13 (1.06-1.20)	1.22 (1.15-1.30)	1.38 (1.29-1.47)
Widowed	1.27 (1.20-1.35)	1.28 (1.21-1.36)	1.13 (1.06-1.21)	1.24 (1.16-1.31)	1.36 (1.27-1.46)
Divorced	1.11 (1.04-1.19)	1.12 (1.04-1.20)	1.11 (1.03-1.20)	1.14 (1.06-1.22)	1.28 (1.19-1.39)
**Deprivation**					
Most Deprived	1.16 (1.09-1.23)	1	1.42 (1.33-1.51)	1.39 (1.31-1.48)	1.35 (1.26-1.44)
Q2	1.13 (1.06-1.19)	1.15 (1.09-1.21)	1.39 (1.31-1.49)	1.35 (1.27-1.43)	1.32 (1.23-1.41)
Q3	1.10 (1.04-1.17)	1.17 (1.11-1.24)	1.20 (1.13-1.28)	1.16 (1.09-1.23)	1.17 (1.09-1.25)
Q4	1.08 (1.01-1.15)	1.28 (1.21-1.36)	1.17 (1.10-1.25)	1.11 (1.04-1.18)	1.11 (1.04-1.19)
Least Deprived	1	1.22 (1.15-1.29)	1	1	1
**Cause of death**					
Cancer	1.45 (1.39-1.52)	2.08 (1.99-2.17)	1	1	2.77 (2.60-2.95)
Organ failure	1	1	1.49 (1.42-1.57)	1.23 (1.17-1.28)	1.08 (1.03-1.14)
Frailty	1.27 (1.21-1.33)	1.44 (1.38-1.51)	1.06 (1.00-1.12)	1.15 (1.09-1.21)	1
**Urban/rural**					
Large Urban Areas	1.53 (1.42-1.65)	1	1.47 (1.35-1.60)	1.92 (1.77-2.07)	1.34 (1.23-1.46)

For peer review only - http://bmjopen.bmj.com/site/about/guidelines.xhtml

Other Urban Areas	1.55 (1.44-1.67)	1.21 (1.16-1.26)	1.43 (1.32-1.55)	2.24 (2.08-2.42)	1.35 (1.24-1.47)
Accessible Small Towns	1.33 (1.22-1.45)	1.28 (1.20-1.36)	1.38 (1.25-1.52)	1.90 (1.74-2.08)	1.26 (1.14-1.40)
Remote Small Towns	1.22 (1.10-1.36)	1.42 (1.30-1.56)	1.23 (1.09-1.38)	1.36 (1.22-1.51)	1.16 (1.03-1.31)
Accessible Rural	1.44 (1.32-1.57)	1.39 (1.31-1.48)	1.22 (1.11-1.34)	1.60 (1.47-1.75)	1.15 (1.04-1.26)
Remote Rural	1	1.38 (1.28-1.50)	1	1	1
**Place of residence**					
Home	1.31 (1.24-1.39)	1.89 (1.79-1.99)	2.72 (2.58-2.87)	2.33 (2.21-2.45)	3.39 (3.21-3.57)
Institution	1	1	1	1	1

*295 patients in "Other/Unknown" categories excluded. "Various" and "External" causes of death not shown in table. Reference categories (OR=1) were chosen so that all ORs >= 1.

For peer review only - http://bmjopen.bmj.com/site/about/guidelines.xhtml

Table 4. Twenty most prevalent Continuous Unscheduled care Pathways (CUPs) by in-hours/out-of-hours start of the CUP for patients (18+) in the last year of their life in Scotland 2016 (N=56,407)

	In-hours (N CUPS =58,157)					Out-of-hours (N CUPS =133,980)			
Rank	Name	Number	%	CUM %		Name	Number	%	CUM %
1	SEA	13,702	23.6	23.6		NO	30,434	22.7	22.7
2	SA	8,639	14.9	38.5		O	18,609	13.9	36.6
3	EA	6,408	11.0	49.5		SEA	9,457	7.1	43.7
4	S	6,014	10.3	59.8		N	8,868	6.6	50.3
5	E	5,085	8.7	68.5		EA	4,923	3.7	54.0
6	SE	3,291	5.7	74.2		NSEA	4,866	3.6	57.6
7	NO	1,450	2.5	76.7		S	4,028	3.0	60.6
8	N	1,230	2.1	78.8		E	3,890	2.9	63.5
9	O	1,005	1.7	80.5		SE	2,816	2.1	65.6
10	NSEA	561	1.0	81.5		NOSEA	2,743	2.0	67.6
11	SEAS	484	.80	82.3		OO	2,697	2.0	69.6
12	SAO	366	.60	82.9		NOSA	2,306	1.7	71.3
13	SAS	305	.50	83.4		NONO	1,677	1.3	72.6
14	SEAO	305	.50	83.9		NON	1,517	1.1	73.7
15	SSEA	285	.50	84.4		NSE	1,305	1.0	74.7
16	SSA	239	.40	84.8		NOO	1,162	.90	75.6
17	SS	229	.40	85.2		NOA	1,112	.80	76.4
18	EAS	221	.40	85.6		SA	1,042	.80	77.2
19	SES	220	.40	86.0		NS	948	.70	77.9
20	NSE	164	.30	86.3		NOEA	924	.70	78.6

* 14,704 missing time stamps

- N=NHS 24 telephone helpline
- O= Primary Care Out of Hours
- S=Scottish Ambulance Service
- E=Emergency Department
- A= Acute hospital admission

For peer review only - http://bmjopen.bmj.com/site/about/guidelines.xhtml

Table 5. Mean number of contacts and costs of NHS24, PCOOH, SAS, A&E attendances and Emergency admissions for patients (18+) in last year of life in Scotland 2016 by cause of death and deprivation status (N=56,407)

	Contact NHS24			Contact PCOOH			Call to SAS			A&E attendance			Emergency Admissions			**All contacts**	
	Mean number of contacts	Total costs (x1000)	Costs per patient	Mean number of contacts	Total costs (x1000)	Costs per patient	Mean number of calls	Total costs (x1000)	Costs per patient	Mean number of atten-dances	Total costs (x1000)	Costs per patient	Mean number of admissions	Total costs (x1000)	Costs per patient	**Total costs to NHS (x1000)**	**Total cost to NHS per patient**
**Total**	1.4	£2,944	£52	1.3	£4,471	£79	1.7	£22,337	£396	1.3	£10,205	£181	1.7	£149,967	£2,659	**£189,923**	**£3,367**
**Cause of death**																	
Cancer	1.4	£836	£53	1.6	£1,502	£94	1.6	£5,807	£365	1.3	£2,765	£174	2.1	£54,022	£3,397	**£64,933**	**£4,083**
Organ failure	1.3	£1,047	£49	1.1	£1,370	£64	2.0	£9,525	£448	1.4	£4,173	£196	1.7	£56,730	£2,670	**£72,845**	**£3,429**
Frailty	1.7	£842	£60	1.6	£1,315	£94	1.5	£4,892	£349	1.1	£2,165	£154	1.3	£28,003	£1,997	**£37,218**	**£2,654**
**Deprivation**																	
Most Deprived	1.5	£748	£55	1.1	£794	£59	1.9	£5,960	£440	1.5	£2,867	£212	1.8	£38,809	£2,867	**£49,177**	**£3,633**
2	1.5	£678	£53	1.3	£984	£77	1.9	£5,543	£433	1.4	£2,559	£200	1.8	£35,805	£2,795	**£45,569**	**£3,557**
3	1.4	£607	£52	1.5	£1,048	£89	1.6	£4,390	£374	1.2	£1,936	£165	1.6	£29,625	£2,522	**£37,606**	**£3,201**
4	1.4	£501	£51	1.6	£929	£94	1.6	£3,644	£370	1.2	£1,569	£159	1.6	£25,050	£2,546	**£31,693**	**£3,221**
Least deprived	1.4	£405	£49	1.4	£710	£85	1.5	£2,766	£333	1.1	£1,256	£151	1.5	£20,349	£2,447	**£25,487**	**£3,065**

For peer review only

Figure 1 – Start and end points of all unscheduled care pathways

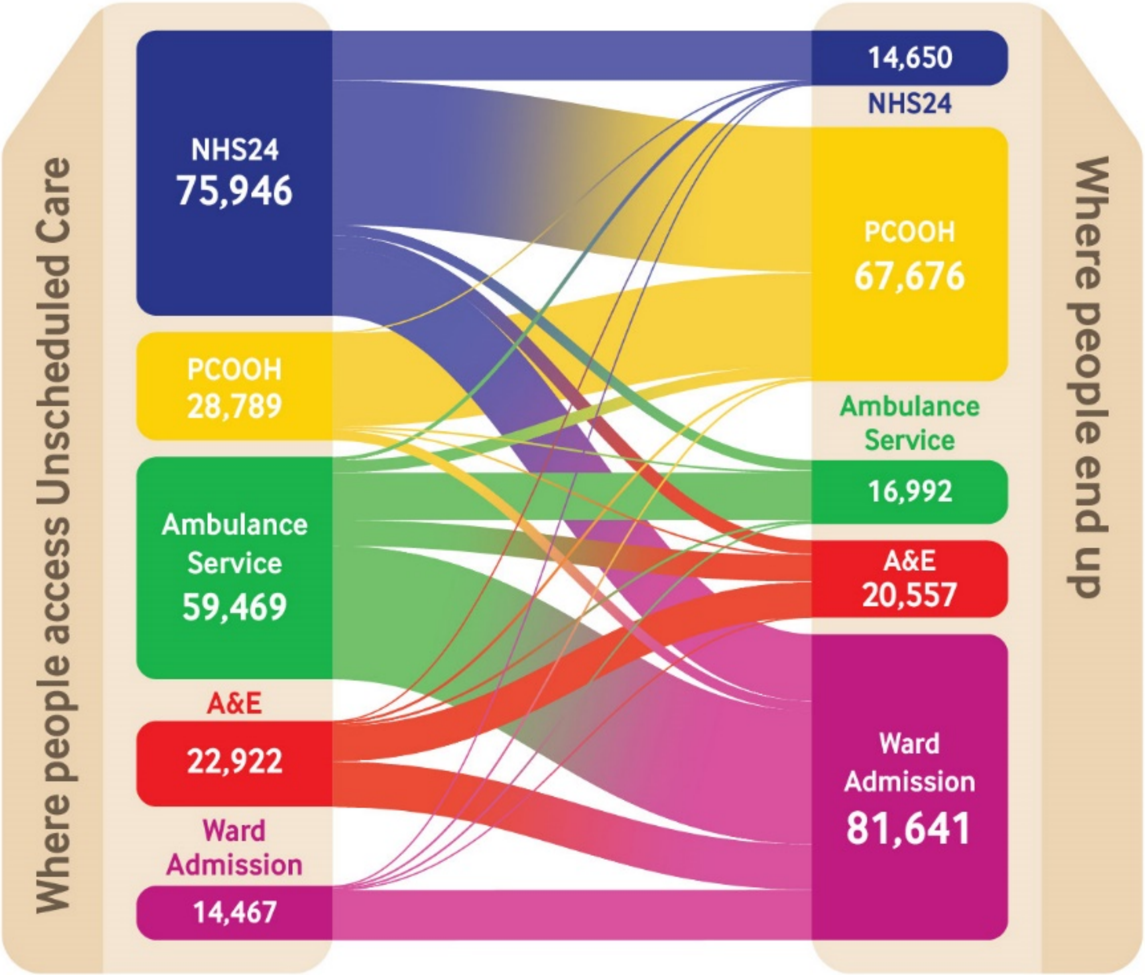

This figure shows the first and final service contacted in each Continuous Unscheduled Care Pathway (CUP). A first contact of telephone advice (NHS24) or Primary Care out-of-hours (PCOOH) was likely to be resolved in primary care. Calling an ambulance or attendance at emergency department (A&E) usually resulted in admission.

- NHS24 = telephone advice
- PCOOH= Primary Care Out of Hours
- A&E=Emergency Department

Note The figure shows 14,467 direct admissions to hospital which were likely to have been via primary care in-hours or outpatient services. Some may also be due to other direct admission routes such as the Scottish Cancer helpline for people receiving cancer treatments.

Supplementary tables

Supplementary table 1. List of ICD10 codes for underlying causes of death grouped into three main categories of conditions with potential palliative care needs (referred to as "illness trajectories")

**Illness trajectories [ITs]**	Sub-ITs	ICD10 codes (v.2010)
**IT1. Cancer *("Clear terminal phase")***		
	1.1 Malignant Neoplasms	C00-C97
**IT2. Organ failure *("Intermittent")***		
	2.1 Endocrine and metabolic diseases	E00-E90<br>*Except: E46*
	2.2 Diseases of the circulatory system	I00-I99<br>*Except: I46*<br>*(I60-I67)*$_{[65+]}$ ****(1/3)***[1]<br>*I69*
	2.3 Diseases of the respiratory system	J00-J99.8<br>*Except: (J06, J09-J16, J18)*$_{[65+]}$ ****(1/3)***[2]<br>*(J20-J22)*$_{[65+]}$ ****(1/3)***[3]<br>*J69.0, J96*
	2.4 Diseases of liver	K70-K77, B18
	2.5 Kidney disease	N17, N18, N28
	2.6 Diseases of the digestive system	K00-K93.8<br>*Except: K70-K77*
	2.7 Other diseases of the genitourinary system	N00-N99.9<br>*Except: N17, N18, N28*

For peer review only

**IT3a. Frailty, Progressive neurological condition [80+]**<br>***("Gradually dwindling")***		
	3.1 Dementia and mental disorders [80+]	A81.0, F01, F03-F07, F09, G30, G31
	3.2 Progressive neurological diseases [80+]	G10, G12.2, G20, G35, G23.1, G90.3
	3.3 Cerebrovascular diseases [80+]	(I60-I67)$_{[80+]}$*(1/3), I69
	3.4 Some ill-defined causes [80+]	R26, R29.6, R53, R54, R55, R63, R64
		I46, J96, R96, R98, R99, W00, W03-08, W10, W18
	3.5 Malnutrition [80+]	E46
	3.6 Infectious causes / Influenza / Pneumonia [80+]	A15-A19.9, B90-B90.9, B20-B24<br>(J06, J09-J16, J18)$_{[80+]}$*(1/3)<br>(J20-J22)$_{[80+]}$*(1/3), J69.0
	3.7 Skin and musculo-skeletal system [80+]	L00-L99.8, M00-M99.9
**IT3b. Frailty, Progressive neurological condition [65-79]**<br>***("Gradually dwindling")***		
	3.8 Dementia and mental disorders [65-79]	A81.0, F01, F03-F07, F09, G30, G31
	3.9 Progressive neurological diseases [65-79]	G10, G12.2, G20, G35, G23.1, G90.3
	3.10 Cerebrovascular diseases [65-79]	(I60-I67)$_{[65-79]}$*(1/3), I69
	3.11 Some ill-defined causes [65-79]	R26, R29.6, R53, R54, R55, R63, R64, I46, J96
	3.12 Malnutrition [65-79]	E46
	3.13 Infectious causes / Influenza / Pneumonia [65-79]	A15-A19.9, B90-B90.9, B20-B24<br>(J06, J09-J16, J18)$_{[65-79]}$*(1/3)

For peer review only

		$(J20\text{-}J22)_{[65\text{-}79]}$ ***(1/3)**
		J69.0
	3.14 Skin and musculo-skeletal system [65-79]	L00-L99.8, M00-M99.9
**IT3c. Frailty, Progressive neurological condition [18-64]** ***("Gradually dwindling")***		
	3.15 Dementia and mental disorders [18-64]	A81.0, F01, F03-F07, F09, G30, G31
	3.16 Progressive neurological diseases [18-64]	G10, G12.2, G20, G35, G23.1, G90.3
	3.17 Cerebrovascular diseases [18-64]	I69
	3.18 Some ill-defined causes [18-64]	R26, R29.6, R53, R54, R55, R63, R64, I46, J96
	3.19 Malnutrition [18-64]	E46
	3.20 Infectious causes / Influenza / Pneumonia [18-64]	A15-A19.9, B90-B90.9, B20-B24, J69.0
	3.21 Skin and musculo-skeletal system [18-64]	L00-L99.8, M00-M99.9
**4. Various causes**		
	4.1 Infectious diseases	A00-B99.9 *Except: A81.0, A15-A19.9, B18, B20-B24, B90-B90.9*
	4.2 Neoplasms + Diseases of the blood	D01-D89.9
	4.3 Diseases of the nervous system - *except progressive neurological diseases*	G00-H95.9 *Except: G10, G12.2, G20, G23.1, G30, G31, G35, G90.3*
	4.4 Symptoms, signs... not elsewhere classified / Ill-defined causes - *except R26, R29.6, R53, R54, R55, R63, R64, and $(R96, R98, R99)_{[80+]}$*	R00-R99 *Except: R26, R29.6, R53, R54, R55, R63, R64, $(R96, R98, R99)_{[80+]}$*

For peer review only

	4.5 Substance abuse and other mental and behavioral disorders	F10-F98
	4.7 Pregnancy, childbirth, puerperium / Congenital malformation	O00-O998, P00-P96.9, Q00-Q99.9
**5. External causes**		
	5. External causes of morbidity and mortality	V01-Y89.9 *Except:(W00, W03-08, W10, W18)[80+]*

[1] One third of the annual deaths with ICD-10 codes (I60-I67) as underlying cause of death (people over 65)

[2] One third of the annual deaths with ICD-10 codes (J06, J09-J16, J18) as underlying cause of death (people over 65)

[3] One third of the annual deaths with ICD-10 codes (J20-J22) as underlying cause of death (people over 65)

The characteristics of the main disease responsible for the death of a patient may be associated with certain health services utilisation patterns in the last months of life. Rather than using every single disease used for underlying causes of death in the International Statistical Classification of Diseases and Related Health Problems ("ICD-10"), we used categories of life-limiting conditions that have been described as having similar courses until death.

The conditions were grouped into three "illness trajectories" ("cancer", "organ failure", "frailty/ progressive neurological conditions"). These trajectories have a shape and an average duration, and they are associated with potential palliative care needs in the people they affect (whether physically, psychologically, socially, existentially or spiritually).

Our goal for this study was to allocate ICD-10 codes used for underlying causes of death in mortality datasets, to these three main categories of conditions ("illness trajectories").

The underlying cause of death is defined as the disease or injury which initiated the chain of events leading directly to death. Every death has just one underlying cause, so is counted only once in figures which are produced on this basis. Deaths are coded in accordance with the ICD-10.

The allocation of ICD-10 codes for underlying causes of death to the three main illness trajectories was completed prior to this study by a panel of international experts in primary care, palliative care, public health and demography, as part of a modified e-Delphi study carried out by two authors of this article (SAM and SM). The panel did not consider contributory causes of death. The list of ICD-10 codes used in this current article has been obtained after two Delphi rounds, and it consists in seven categories (as displayed in Table 1):

1. Cancer
2. Organ failure
3. Frailty/ Progressive neurological conditions [80+]
4. Frailty/ Progressive neurological conditions [65-79]
5. Frailty/ Progressive neurological conditions [18-64]
6. Various causes
7. External causes

For peer review only

The allocation of the total number of deaths to each main category (Scotland, 2016) is featured in Supplementary table 3

For peer review only

Supplementary table 2. Price weight allocation for cost analysis.

We allocated a fixed value for each service used based on average values.

Unscheduled Service	Value	Unit	Notes/Sources
***N**: NHS24 CONTACTS*			
NHS 24 Contact	£30.78	per call	Private communication with NHS24.
***O**: OOH PRIMARY CARE CONSULTATIONS*			
GP OOH	£38.00	per consultation	[note 1]
Nurse OOH	£6.80	per consultation	£0.70 per min x 9.72 mins. [note 1]
OOH Average Unspecified "O"	£33.20	per consultation	Weighted average of GP/Nurse consultation applied where GP/Nurse consultations were pooled. Based on ratio observed in data.
***S**: SCOTTISH AMBULANCE SERVICE (SAS)*			
Conveyed	£238.00	per trip	Applied to a S with an E or A in the CUP [note 2]
Not Conveyed	£184.00	per trip	Applied to a S without an E or A in the CUP [note 2]
OOH Average Unspecified "S"	£228.89	per trip	Weighted average of conveyed/not conveyed based on observed ratio used. Applied where A or E presence is unspecified.
***E**: EMERGENCY DEPT*			
Accident and Emergency Visit	£138.00	per visit	[note 3]
***A**: INPATIENT ADMISSIONS*			
Emergency Inpatient Admissions	£1,590.00	Per Admission	Average non-elective inpatient admission (excluding excess bed days) [note 3].

Notes

Two factors made costing emergency inpatient admissions for this cohort difficult. Firstly, the Scottish data does not include detailed costs for patient procedures we took a general population average cost. Secondly, we did not cost according to length of stay. It is possible that people who died from different underlying causes may have required different average lengths of stay. Some of the cohort may have required longer stays than patients from the general population and possibly more expensive or multiple interventions than the general population. Although the precise figures are uncertain, it is undoubtedly true that any hospital admission is an order of magnitude more expensive than any other unscheduled care service.

1. Curtis L et al. Unit Costs of Health & Social Care 2017. Kent: Personal Social Services Research Unit 2017
2. Hobbs R, Bankhead C, Mukhtar T, Stevens S, Perera-Salazar R, Holt T, & Salisbury C. (2016) Clinical workload in UK primary care: a retrospective analysis of 100 million consultations in England, 2007-14, The Lancet, 387, 10035, 2323-2330. http://www.sciencedirect.com/science/article/pii/S0140673616006206.
3. Department of Health, The. Reference Costs 2016-17 Publication. London: The Department of Health 2017

Supplementary table 3

For peer review only

Supplementary table 3. Number (%) of patients (18+) in the last year of life in Scotland 2016 by underlying cause of death (N=56,407)

Underlying cause of death	N	%
**Cancer (all types)**	**15,902**	**28.2%**
**Organ failure**	**21,244**	**37.7%**
2.1 Endocrine and metabolic	1,137	
2.2 Circulatory	11,212	
2.3 Respiratory	4,744	
2.4 Liver	1,109	
2.5 Kidney	241	
2.6 Digestive	1,949	
2.7 Other genitourinary	852	
**Frailty 80+**	**10,497**	**18.6%**
3.1 Dementia 80+	4,857	
3.2 Progressive neurological 80+	360	
3.3 Cerebrovascular 80+	2,739	
3.4 Ill-defined 80+	258	
3.4 Malnutrition 80+	3	
3.6 Infections & Pneumonia 80+	1,971	
3.7 Skin & Musculo-skeletal 80+	309	
**Frailty 65-79**	**3,076**	**5.5%**
3.8 Dementia 65-79	932	
3.9 Progressive neurological 65-79	332	
3.10 Cerebrovascular 65-79	1,042	
3.11 Ill-defined 65-79	14	
3.12 Malnutrition 65-79	3	
3.13 Infections & Pneumonia 65-79	572	
3.14 Skin & Musculo-skeletal 65-79	181	
**Frailty 19-64**	**450**	**0.8%**
3.15 Dementia 19-64	55	
3.16 Progressive neurological 19-64	180	
3.17 Cerebrovascular 19-64	50	
3.18 Ill-defined 19-64	16	
3.19 Malnutrition 19-64	2	
3.20 Infections & Pneumonia 19-64	63	
3.21 Skin & Musculo-skeletal 19-64	84	
**Various**	**2,271**	**4.0%**
4.1 Infections	648	
4.2 Neoplasms & blood	456	
4.3 Nervous system	395	
4.4 Symptoms & Signs NEC	235	
4.5 Mental & behavioural	419	
4.7 Pregnancy & congenital	118	
**External**	**2,967**	**5.3%**

For peer review only

**Total**	**56,407**	**100.0%**

Supplementary Table 4. Number (%) of contacts with services by patients (18+) in the last year of life in Scotland 2016 according to time between

	Total		Contact NHS 24		Contact PCOOH		Call to SAS		ED attendance		Emergency admissions	
Months	Number	%	Number	%	Number	%	Number	%	Number	%	Number	%
12	18,385	3.9	4,109	4.3	3,584	3.3	3,602	3.8	3,632	4.5	3,458	3.8
11	19,327	4.1	4,200	4.4	3,687	3.4	3,811	4.0	3,919	4.8	3,710	4.0
10	19,779	4.2	4,374	4.6	3,889	3.6	3,866	4.1	3,942	4.9	3,708	4.0
9	21,136	4.5	4,679	4.9	3,993	3.7	4,056	4.3	4,236	5.2	4,172	4.5
8	22,374	4.7	4,818	5.1	4,306	3.9	4,375	4.6	4,353	5.4	4,522	4.9
7	24,006	5.1	5,164	5.4	4,681	4.3	4,667	4.9	4,625	5.7	4,869	5.3
6	25,542	5.4	5,391	5.7	5,022	4.6	5,102	5.4	4,832	6.0	5,195	5.7
5	28,759	6.1	5,902	6.2	5,652	5.2	5,728	6.0	5,463	6.7	6,014	6.6
4	33,334	7.1	6,579	6.9	6,624	6.1	6,725	7.1	6,250	7.7	7,156	7.8
3	41,634	8.8	7,955	8.3	8,319	7.6	8,616	9.1	7,550	9.3	9,194	10.0
2	56,473	12.0	10,428	10.9	11,567	10.6	11,888	12.5	9,781	12.1	12,809	14.0
1	161,611	34.2	31,751	33.3	47,833	43.8	32,583	34.3	22,488	27.7	26,956	29.4
Total	472,360	100	95,350	100	109,157	100	95,019	100	81,071	100	91,763	100

For peer review only

STROBE (Strengthening The Reporting of OBservational Studies in Epidemiology) Checklist

A checklist of items that should be included in reports of observational studies. You must report the page number in your manuscript where you consider each of the items listed in this checklist. If you have not included this information, either revise your manuscript accordingly before submitting or note N/A.

Note: An Explanation and Elaboration article discusses each checklist item and gives methodological background and published examples of transparent reporting. The STROBE checklist is best used in conjunction with this article (freely available on the Web sites of PLoS Medicine at http://www.plosmedicine.org/, Annals of Internal Medicine at http://www.annals.org/, and Epidemiology at http://www.epidem.com/). Information on the STROBE Initiative is available at www.strobe-statement.org.

Section and Item	Item No.	Recommendation	Reported on Page No.
**Title and Abstract**	1	(*a*) Indicate the study's design with a commonly used term in the title or the abstract	
		(*b*) Provide in the abstract an informative and balanced summary of what was done and what was found	
**Introduction**			
Background/Rationale	2	Explain the scientific background and rationale for the investigation being reported	
Objectives	3	State specific objectives, including any prespecified hypotheses	
**Methods**			
Study Design	4	Present key elements of study design early in the paper	
Setting	5	Describe the setting, locations, and relevant dates, including periods of recruitment, exposure, follow-up, and data collection	
Participants	6	(*a*) *Cohort study*—Give the eligibility criteria, and the sources and methods of selection of participants. Describe methods of follow-up<br>*Case-control study*—Give the eligibility criteria, and the sources and methods of case ascertainment and control selection. Give the rationale for the choice of cases and controls<br>*Cross-sectional study*—Give the eligibility criteria, and the sources and methods of selection of participants	
		(*b*) *Cohort study*—For matched studies, give matching criteria and number of exposed and unexposed<br>*Case-control study*—For matched studies, give matching criteria and the number of controls per case	
Variables	7	Clearly define all outcomes, exposures, predictors, potential confounders, and effect modifiers. Give diagnostic criteria, if applicable	

For peer review only

Section and Item	Item No.	Recommendation	Reported on Page No.
Data Sources/ Measurement	8*	For each variable of interest, give sources of data and details of methods of assessment (measurement). Describe comparability of assessment methods if there is more than one group	
Bias	9	Describe any efforts to address potential sources of bias	
Study Size	10	Explain how the study size was arrived at	
Quantitative Variables	11	Explain how quantitative variables were handled in the analyses. If applicable, describe which groupings were chosen and why	
Statistical Methods	12	(*a*) Describe all statistical methods, including those used to control for confounding	
		(*b*) Describe any methods used to examine subgroups and interactions	
		(*c*) Explain how missing data were addressed	
		(*d*) *Cohort study*—If applicable, explain how loss to follow-up was addressed<br>*Case-control study*—If applicable, explain how matching of cases and controls was addressed<br>*Cross-sectional study*—If applicable, describe analytical methods taking account of sampling strategy	
		(*e*) Describe any sensitivity analyses	
**Results**			
Participants	13*	(a) Report numbers of individuals at each stage of study—eg numbers potentially eligible, examined for eligibility, confirmed eligible, included in the study, completing follow-up, and analysed	
		(b) Give reasons for non-participation at each stage	
		(c) Consider use of a flow diagram	
Descriptive Data	14*	(a) Give characteristics of study participants (eg demographic, clinical, social) and information on exposures and potential confounders	
		(b) Indicate number of participants with missing data for each variable of interest	
		(c) *Cohort study*—Summarise follow-up time (eg, average and total amount)	
Outcome Data	15*	*Cohort study*—Report numbers of outcome events or summary measures over time	
		Case-control study—Report numbers in each exposure category, or summary measures of exposure	
		Cross-sectional study—Report numbers of outcome events or summary measures	

For peer review only

Section and Item	Item No.	Recommendation	Reported on Page No.
Main Results	16	(*a*) Give unadjusted estimates and, if applicable, confounder-adjusted estimates and their precision (eg, 95% confidence interval). Make clear which confounders were adjusted for and why they were included	
		(*b*) Report category boundaries when continuous variables were categorized	
		(*c*) If relevant, consider translating estimates of relative risk into absolute risk for a meaningful time period	
Other Analyses	17	Report other analyses done—eg analyses of subgroups and interactions, and sensitivity analyses	
**Discussion**			
Key Results	18	Summarise key results with reference to study objectives	
Limitations	19	Discuss limitations of the study, taking into account sources of potential bias or imprecision. Discuss both direction and magnitude of any potential bias	
Interpretation	20	Give a cautious overall interpretation of results considering objectives, limitations, multiplicity of analyses, results from similar studies, and other relevant evidence	
Generalisability	21	Discuss the generalisability (external validity) of the study results	
**Other Information**			
Funding	22	Give the source of funding and the role of the funders for the present study and, if applicable, for the original study on which the present article is based	

*Give information separately for cases and controls in case-control studies and, if applicable, for exposed and unexposed groups in cohort and cross-sectional studies.

Once you have completed this checklist, please save a copy and upload it as part of your submission. DO NOT include this checklist as part of the main manuscript document. It must be uploaded as a separate file.

For peer review only

BMJ Open

Unscheduled and out-of-hours care for people in their last year of life: a retrospective cohort analysis of national datasets

Journal:	*BMJ Open*
Manuscript ID	bmjopen-2020-041888.R1
Article Type:	Original research
Date Submitted by the Author:	09-Sep-2020
Complete List of Authors:	Mason, Bruce; University of Edinburgh<br>Kerssens, Joannes; NHS National Services Scotland, Information Services Division<br>Stoddart, Andrew; University of Edinburgh,<br>Murray, Scott; The University of Edinburgh Edinburgh Medical School, Primary Pallaitive Care Research Group, The Usher Institute,<br>Moine, Sébastien ; University of Edinburgh, 3. Primary Palliative Care Research Group, Usher Institute of Population Health Sciences and Informatics; University of Paris 13, Health Education and Practices Laboratory<br>Finucane, Anne; Marie Curie Hospice Edinburgh<br>Boyd, Kirsty; University of Edinburgh, Primary Palliative Care Research Group
**Primary Subject Heading**:	Health services research
Secondary Subject Heading:	Emergency medicine, General practice / Family practice, Palliative care, Public health
Keywords:	PRIMARY CARE, ACCIDENT & EMERGENCY MEDICINE, Adult palliative care < PALLIATIVE CARE, HEALTH SERVICES ADMINISTRATION & MANAGEMENT, PUBLIC HEALTH, STATISTICS & RESEARCH METHODS

SCHOLARONE™
Manuscripts

I, the Submitting Author has the right to grant and does grant on behalf of all authors of the Work (as defined in the below author licence), an exclusive licence and/or a non-exclusive licence for contributions from authors who are: i) UK Crown employees; ii) where BMJ has agreed a CC-BY licence shall apply, and/or iii) in accordance with the terms applicable for US Federal Government officers or employees acting as part of their official duties; on a worldwide, perpetual, irrevocable, royalty-free basis to BMJ Publishing Group Ltd ("BMJ") its licensees and where the relevant Journal is co-owned by BMJ to the co-owners of the Journal, to publish the Work in this journal and any other BMJ products and to exploit all rights, as set out in our licence.

The Submitting Author accepts and understands that any supply made under these terms is made by BMJ to the Submitting Author unless you are acting as an employee on behalf of your employer or a postgraduate student of an affiliated institution which is paying any applicable article publishing charge ("APC") for Open Access articles. Where the Submitting Author wishes to make the Work available on an Open Access basis (and intends to pay the relevant APC), the terms of reuse of such Open Access shall be governed by a Creative Commons licence – details of these licences and which Creative Commons licence will apply to this Work are set out in our licence referred to above.

Other than as permitted in any relevant BMJ Author's Self Archiving Policies, I confirm this Work has not been accepted for publication elsewhere, is not being considered for publication elsewhere and does not duplicate material already published. I confirm all authors consent to publication of this Work and authorise the granting of this licence.

For peer review only

Unscheduled and out-of-hours care for people in their last year of life: a retrospective cohort analysis of national datasets

Bruce Mason, Joannes Kerssens, Andrew Stoddart, Scott A. Murray, Sebastien Moine, Anne M. Finucane, Kirsty Boyd

Primary Palliative Care Research Group, Usher Institute, University of Edinburgh, EH8 9AG Scotland, UK Bruce Mason, senior researcher

Electronic Data Research and Innovation Service, Public Health Scotland, Little France Road, Edinburgh, EH16 4UX Scotland, UK Joannes Kerssens, principal information analyst and statistician

Edinburgh Clinical Trials Unit, Usher Institute, University of Edinburgh, Scotland, EH8 9AG UK Andrew Stoddart, senior health economist[3]

Primary Palliative Care Research Group, Usher Institute, University of Edinburgh, Scotland, EH8 9AG UK Scott A. Murray, emeritus professor[1]

Health Education and Practices Laboratory, University of Paris 13, Bobigny, France. Sebastien Moine, senior visiting fellow at Usher Institute, Edinburgh, UK

Marie Curie Hospice, 45 Frogston Road West, Edinburgh, EH10 7DR, Scotland, UK. Anne M. Finucane, research lead

Primary Palliative Care Research Group, Usher Institute, University of Edinburgh, Scotland, EH8 9AG UK Kirsty Boyd, reader

Correspondence to Scott A Murray, Primary Palliative Care Research Group, Usher Institute, University of Edinburgh, Scotland, EH8 9AG UK Scott.Murray@ed.ac.uk

Key words: Out-of-hours care, unscheduled care, routine data, primary care, care pathway

For peer review only - http://bmjopen.bmj.com/site/about/guidelines.xhtml

Summary:

Objectives To analyse patterns of use and costs of unscheduled NHS services for people in the last year of life.

Design Retrospective cohort analysis of national datasets with application of standard UK costings

Participants and setting All people who died in Scotland in 2016 aged 18 or over (n= 56,407)

Main outcome measures Frequency of use of the five unscheduled NHS services in the last 12 months of life by underlying cause of death; patient demographics; Continuous Unscheduled Care pathways (CUPs) followed by patients during each care episode; total NHS and per-patient costs.

Results 53,509 patients (94.9%) had at least one contact with an unscheduled care service during their last year of life: a total of 472,360 contacts, with 34.2% in the last month of life. By linking patient contacts during each episode of care, we identified 206,841 Continuous Unscheduled Care pathways (CUPs); with 133,980 (64.8%) starting out-of-hours. People with cancer were more likely to contact the NHS telephone advice line (p<.001) or primary care out-of-hours (p<.001) and have emergency admissions (p<.001). People with organ failure contacted the ambulance service most frequently (p<.001). Demographic factors associated with more unscheduled care were older age, social deprivation, living in own home, and dying of cancer. People dying with organ failure formed the largest group in the cohort which had the highest NHS costs overall. The cost of providing services in the community was estimated at 3.9 % of total unscheduled care costs despite handling most out-of-hours calls.

Conclusions Over 90% of people used NHS unscheduled care in their last year of life; more frequently than has been reported previously. Different underlying causes of death and demographic factors impacted on initial access and subsequent pathways of care. Managing more unscheduled care episodes in the community has the potential to reduce hospital admissions and overall costs.

For peer review only - http://bmjopen.bmj.com/site/about/guidelines.xhtml

Article summary

Strengths and limitations of this study

- Linking death certification codes with service use in the 12 months before death allowed us to analyse the impact of different illnesses on unscheduled care service use.
- We linked all five unscheduled NHS services (telephone advice, primary care, ambulances, emergency department and hospital admission) into Continuous Unscheduled Pathways (CUPs) and identified common patterns of unscheduled care for people.
- This innovative, population based method provided a broad understanding of how different demographic factors affected use of unscheduled care throughout Scotland.
- Only one of the datasets, primary care out-of-hours, contained information that could indicate whether a person was identified for palliative care.
- Costs were calculated using weighted averages and were therefore approximate.

Introduction

Rising demand for unscheduled care is a major burden and causes pressure on healthcare systems internationally, both in and out-of-hours. Unscheduled care is unplanned and demand-led and free at point of access. In the UK, it includes five NHS services: telephone advice, primary care services, ambulance services, emergency department and acute hospital admission. Emergency department targets were unmet throughout the UK prior to the impact of Covid-19. Unplanned hospital admissions in the UK increased by 28% from 2010-2019, while elective admissions rose by 25% during the same time period. [1] Contributory factors include an aging population with multiple health conditions, public expectations, instructions to seek urgent care for suspected strokes or heart attacks, and less support in the community at weekends and overnight. [2] Much unscheduled care is used by people in their last year of life who are known to have significant health-related suffering and unmet palliative care needs. [3]

For peer review only - http://bmjopen.bmj.com/site/about/guidelines.xhtml

Early integration of a palliative approach as a component of chronic disease management is strongly recommended for people with advanced illnesses in all care settings. [4,5] Palliative care is a core component of universal health coverage and its people-centered ethos and focus on quality of life and death should be considered at every opportunity. [6] Services should be designed to respond to the typical trajectories of declining health of patients with all progressive illnesses, including social and psychological factors. [7, 8] Palliative care includes proactive care planning which reduces burdensome interventions of low benefit, and helps avoid some unwarranted hospital admissions. However, palliative care integration into unscheduled care services has proven particularly challenging. [9, 10]

National datasets can be used to monitor and improve care. They have underutilised potential to improve end-of-life care. [11-15] In Scotland, death registry data, and activity data from hospitals, the ambulance service, and out-of-hours primary care services are collected routinely. These national datasets contain a unique identifier—the Community Health Index (CHI). We set out to link these data to analyse patterns of unscheduled care services use and costs by underlying cause of death and patient demographics.

Methods

We linked three datasets. The *National Records of Scotland (NRS) deaths dataset* was used to identify all adults (aged 18+) who died in Scotland in 2016. From this dataset we extracted underlying cause of death and usual place of residence. From the General Acute Inpatient and Day Case - Scottish Morbidity Record (SMR01) we extracted all unscheduled hospital activity for the last 12 months life for the cohort. Thirdly, we extracted data from the *Scottish Unscheduled Care Datamart* (UCD) covering four unscheduled care services: the telephone advice line (NHS24), primary care out-of-hours (PCOOH), the Scottish ambulance service (SAS), and emergency department attendances (ED). [16] The UCD does not include in-hours, unscheduled primary care.

For peer review only - http://bmjopen.bmj.com/site/about/guidelines.xhtml

The ICD codes (v 2010) for underlying cause of death extracted from the NRS dataset were classified into five groups: cancer, organ failure, frailty/progressive neurological conditions, various other causes, and external causes.[17] People in the first three groups were considered potentially to have had palliative care needs during their last year of life. Details of this coding allocation had been agreed previously by an expert international panel. [18] (See online supplementary Table 1)

Postcodes of usual place of residence were extracted from the NRS dataset. We used the Scottish Index of Multiple Deprivation (SIMD 2016) to infer quintiles of deprivation. [19] Rurality was based on the Scottish Government Urban-Rural Classification as applied to the postcodes. [20]

In order to understand how multiple services were accessed during a single healthcare episode, we used Continuous Unscheduled Pathways (CUPs) as defined in the Scottish unscheduled care datamart. A CUP is a linked set of contacts with one or more unscheduled care services. [16] Each CUP represents a single patient journey. The frequencies of the different types of CUP were tabulated to identify key patterns. We categorised a CUP as 'out-of-hours' if it started at weekends, on public holidays or on weeknights from 6pm to 8am. There is no limit to the duration of each CUP so a CUP could start out-of-hours but end during the in-hours period and vice-versa. Therefore, only the start date and time of the CUP was used to categorise it.

In the Unscheduled Care Dataset, each service component of a CUP is assigned a code letter:

- N=NHS 24
- O= Primary Care Out of Hours
- S=Scottish Ambulance Service
- E=Emergency Department
- A= Acute hospital admission

For peer review only - http://bmjopen.bmj.com/site/about/guidelines.xhtml

Linking these codes in chronological order gives the pathway its name. For example, 'NSE' represents a call to NHS 24 (N), followed by an ambulance service contact (S), then an emergency department attendance (E).

Statistical analysis: Descriptive analyses were undertaken using means and frequency tables. Service use of people with or without unscheduled care contacts were compared using chi-squared tests for categorical variables. Multiple logistic regression models analysed multivariate associations between predictor variables (gender, age, marital status, deprivation quintile, cause of death, urban/rural classification and place of residence) and the odds of using a service. All analyses were conducted within the Scottish National Safe Haven by a senior analyst (JK) after approval by a Scottish Public Benefit and Privacy Panel (PBPP).

Cost estimations: Standard UK price weighting methodology was applied to estimate the costs of each unscheduled service: see online supplementary Table 2 to explain how this was calculated. We did not attribute exact pricing to different forms of inpatient admission or account for differences due to patient demographics hence mean population values were applied. These costs are included as broad indicators of differences in scale and should not be interpreted as exact data.

Patient and Public Involvement: Representatives from Marie Curie Expert Voices Scotland and a Royal College of General Practitioners Scottish patient group joined the steering group and contributed public-patient perspectives from their own groups throughout the project. Key stakeholders from the unscheduled services and patient group members advised the research team on parameters for analysis, choice of analyses, and data interpretation. To understand decision-making and experiences of service users, we conducted focus groups and interviews with patients with advanced illnesses and carers who had used unscheduled services, and with bereaved carers. A final key stakeholder meeting of professionals, policy makers and lay representatives discussed the findings and implications for service development. These data will be reported elsewhere.

For peer review only

For peer review only - http://bmjopen.bmj.com/site/about/guidelines.xhtml

Results

1 Underlying causes of death in the cohort

We extracted records for 56,407 adults who died in Scotland in 2016 and linked records covering the last 12 months of life for each individual in this cohort. The number of people in each disease group was as follows: cancer 28.2%, organ failure 37.7%, frailty/progressive neurological conditions 24.9%, other diseases / various causes 4.0%, and external causes 5.3% (see: online supplementary Table 3).

2 Use of NHS unscheduled care services

The cohort had 472,360 unscheduled care service contacts; 56,407 people (94.9% of the cohort) had at least one contact. Table 1 shows the distribution of unscheduled service use: 50.4% had 6 or more contacts, and the 5.4% who had 20 or more contacts accounted for 21.5% of all contacts. All unscheduled care services were used increasingly as death approached, with 34.2% occurring in the last month of life (see online supplementary Table 4). During that final month, there was a disproportionate rise in primary care out-of-hours workload.

Table 2 displays the number and percentages of people who contacted the five unscheduled care services during the last 12 months of life by cause of death and demographic factors. Place of residence at death had two categories: those living in a private residence or people living in any institution. The latter were primarily care homes, but also included prisons and hostels. Due to the large sample size, all differences in Table 2 were statistically significant (except contacts with NHS 24 by deprivation).

24-hour Telephone Advice Service (NHS24)

More people dying with frailty (66.0%) or cancer (63.4%) contacted this service than those dying with organ failure (56.0%) (p<.001). People living in institutions (or commonly the staff caring for them) were more likely to contact NHS24 than those living at home: 68.8% v 58.0% (p<.001). People

For peer review only - http://bmjopen.bmj.com/site/about/guidelines.xhtml

from all deprivation quintiles were about as likely to access NHS24 (p<.758). People who lived in urban areas contacted NHS24 more than those from remote areas: 58.7 v 49.0% (p<.001).

Primary Care Out-of-Hours (PCOOH)

Service use was similar to NHS24, as more people dying with cancer (61.5%) or frailty (61.7%) had contact with this service compared to those with organ failure (45.6%) (p<.001). People living in an institution were substantially more likely to have used this service than those in a private residence: 69.3% v 50.7% (p<.001). People living in the most deprived quintile were less likely to access PCOOH (46.6%) compared to those from the least deprived quintile (56.9%)(p<.001).

Scottish Ambulance Service

People who died from organ failure used this service (78.9%) more than those with cancer (72.8%) or frailty (67.8%) (p<.001). People living at home were much more likely to phone the ambulance service than those in an institution (77.2% v 55.7%) (p<.001). People in the most deprived quintile accessed help from the ambulance service more often (77.2%) than people in the least deprived. (69.5%) (p<.001).

Emergency Department

People who died from organ failure used this service (65.2%) more than those with cancer or frailty (p<.001), just as they did with the ambulance service. People living in institutions were less likely to visit an emergency department than those living at home (46.7% v 65.6%) (p<.001). Those from the most deprived quintile were more likely to attend an emergency department (67.3%) compared to people in the least deprived quintile (61.3%) (p<.001).

Acute Hospital Admission

More people who died from cancer had at least one acute hospital admission (88.3%) compared to those with organ failure (73.2%) or frailty (66.4%) (p<.001). People living at home were much more

For peer review only - http://bmjopen.bmj.com/site/about/guidelines.xhtml

For peer review only

likely to be admitted to hospital (79.3%) than those living in an institution (50.6%) (p<.001). People in the most deprived quintile were admitted more often (75.5%) than people in the least deprived. (70.8%) (p<.001).

Patterns of use across unscheduled care services

Logistic regression modelling included the clinical, socioeconomic and location variables from Table 2 plus age, gender, and marital status. (Table 3) The odds ratio (OR) for contacts with NHS24 was higher for women than men (adjusted OR 1.17, 95% CI = 1.12-1.21). People aged 65-84 (adjusted OR 1.68, 95% CI = 1.58-1.78) and those aged 85 or over (adjusted OR 1.67, 95% CI = 1.56-1.79) had higher odds ratios for acute hospital admission compared to people aged 18-64. Comparing the three main groups of causes of death, the odds of people with cancer having a PCOOH contact in their last year of life was much greater than for people dying with organ failure (adjusted OR 2.08, 95% CI = 1.99-2.17). After controlling for demographic and location variables, individuals with cancer had a higher risk of an acute admission than people with organ failure (adjusted OR 2.56, 95% CI = 2.28-2.86). Overall, people living at home used more unscheduled care services than those in institutions. This was particularly the case for acute hospital admissions (adjusted OR 3.39, 95% CI = 3.21-3.57).

People in the most deprived quintile tended to use the three unscheduled care services that are not community based more than those in the least deprived quintile. This was especially so for ambulance services (adjusted OR 1.42, 95% CI = 1.33-1.51). Those living in urban areas used more of all the services except PCOOH than people from rural areas overall.

3. Use of Continuous Unscheduled Pathways (CUPs)

Linking the serial use of the unscheduled service contacts into CUPs enabled us to delineate common sequences and patterns of service use. Table 4 shows the 20 most common CUPs according to when they started; in-hours or out-of-hours. NHS24 and the ambulance service were the most

For peer review only - http://bmjopen.bmj.com/site/about/guidelines.xhtml

common initial access points. The most common end points were PCOOH and hospital admission. (Figure 1) Over half the ambulance calls led to an acute hospital admission (60.0%). Conversely, 50.6% of initial calls to NHS24 were dealt with in the community by PCOOH.

Differences in frequencies and types of CUP by start time

We differentiated between CUPs that started out-of-hours or in-hours to look at implications for improving out-of-hours care as well as unscheduled care in general. We identified 206,841 continuous unscheduled pathways, of which 133,980 (64.8%) started out-of-hours, 28.1% in-hours, and 7.1% unknown (mostly due to lack of a time-stamp on acute hospital admissions). Contacts with NHS24 and PCOOH were much more frequent for out-of-hours CUPs. Data on the proportion of contacts with each service that occurred during out-of-hours CUPs were: NHS24 93.1%, PCOOH 94.7%, ambulance service 37.7%, and emergency department 44.4%.

Table 4 data allows a detailed understanding of how patients typically move through the services night and day. Only 16.7% of out-of-hours CUPs started with an ambulance call while 73.2% started with an NHS24 or PCOOH contact. Similarly, most out-of-hours CUPs ended in primary care: 9.6% with telephone advice from NHS24 and 46.7% with PCOOH. Much fewer out-of-hours CUPs resulted in an acute hospital admission (27.5%) or emergency department attendance (8.2%). In contrast, the six most common CUPs which started in-hours comprised episodes consisting of ambulance calls, emergency department visits and acute hospital admissions, and these accounted for 74.2% of all CUPs which started in-hours. GP in-hours care is not included in the UCD so was not available.

4. Costs

The mean number of contacts, per patient costs and total NHS costs for the five unscheduled care services in the last year of life are listed by underlying causes of death and deprivation status in Table 5. People with organ failure formed the largest group which had the highest total NHS costs due to use of hospital services. Those with frailty incurred the least unscheduled NHS care costs,

For peer review only - http://bmjopen.bmj.com/site/about/guidelines.xhtml

For peer review only

being managed more in the community. The total cost of unscheduled NHS care in Scotland for people in their last year of life was nearly £190 million, of which only 3.9% was for provision of primary care services.

The total mean per patient costs of unscheduled care in the last year of life were greatest for those with cancer (£4,083), followed by organ failure (£3,429), and frailty (£2,654). Unscheduled per patient costs for people in the most deprived quintile were 18.8% higher than those from the least deprived but their PCOOH costs per capita were 30.6% lower.

Discussion

Principal findings

We found that 94.9% of people had unscheduled care contacts during their last year of life. They had a median of five contacts, with 5.1% making 20 or more, and 34.2% of all contacts occurring during the final month of life. We identified three groups of patients by underlying cause of death with different patterns of unscheduled care service use that were clinically and statistically significant. People with cancer had more unscheduled admissions than people with non-cancer diagnoses and the highest per patient costs. People who died with frailty were most likely to have unscheduled care that was managed fully in the community. People with organ failure used most ambulance services, and as a group accounted for the greatest number of acute hospital admissions overall. People from the most deprived quintile used significantly less PCOOH than those from the least deprived but they accessed the other four unscheduled services more. The total cost of service use by people in the most deprived quintile was almost double that of those in the least deprived, due to the greater numbers of people dying in this group and their higher use of secondary care services. NHS24 and PCOOH services together accounted for less than 4% of total NHS unscheduled care costs.

Strengths and limitations of the study

For peer review only - http://bmjopen.bmj.com/site/about/guidelines.xhtml

Strengths This innovative approach to studying population use of interconnected, unscheduled care services provided a broad understanding of how different illnesses, and demographic factors affected use of unscheduled care. Linking all five unscheduled NHS services together into patient pathways (CUPs) allowed common patterns of unscheduled care to be identified and quantified. Studying the unscheduled care service pathways of people in their last year of life throughout Scotland enabled us to analyse the unscheduled care provided for a whole population which has rising numbers of people with unidentified palliative care needs.[3] Understanding the perspectives and choices made by people and their families seeking unscheduled care is equally important and was the qualitative data component of our overall study (to be reported elsewhere).

Limitations The structure and scope of unscheduled care services in Scotland influenced how those services were used so comparisons with different countries and healthcare systems will need care. Lack of national data for in-hours, unscheduled primary care was limiting. Some CUPs recorded as out-of-hours may have started with an undocumented, urgent primary care contact in-hours. Timing of acute hospital admissions is not recorded so could not be separated by starting point. Costs were calculated using weighted averages and were therefore approximate. We acknowledge limitations in relying on ICD-10 recorded diagnoses. We had intended to look for evidence that patients had been identified for palliative care. Unfortunately, only the primary care out-of-hours dataset had a palliative care code or recorded access to the Scottish electronic care plan (Key Information Summary) used by primary care teams to coordinate palliative and anticipatory care planning. [21] This meant it was impossible to estimate the full extent of proactive care planning or palliative care provision by NHS unscheduled care services.

Comparison with other studies

Most studies of unscheduled care have focussed on individual services and specific diseases, notably emergency departments and patients with cancer, and have suggested that only 30-35% of people with cancer use unscheduled care in the last year of life. [9, 22] However, a recent study in one

For peer review only - http://bmjopen.bmj.com/site/about/guidelines.xhtml

Scottish region reported that 78% of people dying from cancer had unscheduled care provided by emergency departments and/or PCOOH in their last year of life. [23] Our findings, integrating all five NHS unscheduled care services, found that 94.9% of people who died in Scotland received unscheduled care in their last year of life. By using population data along with specific service use data, we have highlighted the extent and diversity of unscheduled care pathways. Our logistic regression models identified differences in unscheduled care by deprivation quintile for each of the three main illness groups, not just for the population with cancer, and correlates with other evidence around the relationship between socioeconomic status and use of emergency services. [9, 11, 13]

Meaning of the study and implications for clinicians and policymakers

Many more people seek unscheduled and out-of-hours care in their last year of life than was recognised previously. There were common patterns associated with different underlying illnesses, deprivation status and place of residence. Knowledge of how these groups of patients respond to urgent care needs may help community and hospital services find ways to respond more effectively and potentially could reduce demand for costly services. Primary care teams, social care managers and hospital teams can identify frequent or unusual patterns of contacts with unscheduled care and use these to trigger new or updated care planning. Such care planning communicated to unscheduled care services routinely via primary care managed electronic care co-ordination systems has been linked with fewer hospital admissions and deaths in Scotland. [21, 24] In London, the "Coordinate my Care" system uploads and shares urgent care plans entered by primary care, ambulance and hospital services thereby reducing hospital admissions, and electronic care planning systems are evolving in other parts of England. [10, 25] The UK ReSPECT process partners with patients and families to make emergency treatment and care plans that can help guide unscheduled care and reduce unwarranted admissions. [26] Key aspects are early identification of people at risk

For peer review only - http://bmjopen.bmj.com/site/about/guidelines.xhtml

of deteriorating health, proactive care planning, and a readily accessible electronic care coordination system that can be read and updated by any professional responsible for a person's care.

Improving the ability of unscheduled primary care services to manage people in the community is likely to be highly cost-effective as well as supporting people's choice to remain at home towards the end of life. Emergency departments are already employing more primary care clinicians to enhance pre-hospital triage and ambulance crews are providing more care at home, where appropriate, instead of transferring patients to hospital. [27] Interventions in the community by NHS 24, PCOOH and ambulance services have potential to provide high- value low-cost care.

Further research

Unscheduled care of the whole population merits ongoing research using population-level data, encompassing all community and hospital settings both in-hours and out-of-hours. Improving the scope and quality of data collected routinely can facilitate research into the needs of people who are high service users and stand to benefit from better coordinated care. [5, 6, 28] Specifically, research to understand specific differences in care pathways and service use is important, such as why people with cancer have more urgent hospital admissions than others and why people with organ failure call the ambulance service relatively frequently. Our data also provides a baseline that can be used in studies to evaluate changes in the use of unscheduled services during the coronavirus pandemic, when the demand for hospital-based unscheduled care dipped sharply.

Interventions to encourage a palliative care approach in each of the five out-of-hours services, as well as care coordination throughout the unscheduled pathways are recommended. [29] An evaluation of telephone advice services including emergency social care, community nurse telephone support such as the Gold Line, specialist palliative care support lines, and from charities offering help for people with specific illnesses is indicated to scope provision of unscheduled care in the community further. [29] Research into the contribution of specialist palliative care out-of-hours,

For peer review only - http://bmjopen.bmj.com/site/about/guidelines.xhtml

which has not been included in this study, and interventions to co-ordinate care between settings at the end of life are also needed. [29]

Conclusions

The extent of unscheduled care delivered to people in their last year of life is significantly greater and more varied than reported previously. People with diverse urgent care needs are accessing these services at high levels, particularly in their final month of life. More should be done to take account of underlying illness trajectories and social determinants of health, including better public understanding of how to access the right care in timely and effective ways. Systematic approaches to care planning combined with effective recording and sharing of key information, including a palliative care code where appropriate, is vital and should be recorded in routine healthcare datasets.

Figure legend

Figure 1 – Start and end points of all unscheduled care pathways

Acknowledgments:

The authors acknowledge the support of National Services Scotland for their involvement of obtaining approvals, provisioning and data linkage. We also acknowledge advice from the wider project steering group: Marilyn Kendall, senior researcher; Sheonad Laidlaw, general practitioner; Emma Carduff, honorary research fellow and Marie Curie research lead; Erna Haraldsdottir, senior lecturer; Sir Lewis Ritchie, Scottish Government advisor; Sian Tucker, NHS24 national lead; Marie Fallon, professor of palliative medicine; Jeremy Keen, consultant in palliative medicine; Stella Macpherson, patient and public involvement representative; Nicole Brun, project administrator. We also thank Bruce Guthrie and Sarah Mills for commenting on a draft of this paper.

Contributor and guarantor information: BM, SAM, SM, AF, AS and KB designed the study (along with members of the steering group acknowledged above), JK and AS linked and analysed the data, SM allocated illness trajectories to all decedents, and AF conducted literature reviews. All contributors discussed the findings and contributed to writing the drafts and the final paper. The contributors met with the steering group regularly to design and guide the overall study and to integrate patient and carer perspectives. SAM and KB are the guarantors. The corresponding author attests that all listed authors meet authorship criteria and that no others meeting the criteria have been omitted.

For peer review only - http://bmjopen.bmj.com/site/about/guidelines.xhtml

Copyright /license for publication: The Corresponding Author has the right to grant on behalf of all authors and does grant on behalf of all authors, a worldwide licence to the Publishers and its licensees in perpetuity, in all forms, formats and media (whether known now or created in the future), to i) publish, reproduce, distribute, display and store the Contribution, ii) translate the Contribution into other languages, create adaptations, reprints, include within collections and create summaries, extracts and/or, abstracts of the Contribution, iii) create any other derivative work(s) based on the Contribution, iv) to exploit all subsidiary rights in the Contribution, v) the inclusion of electronic links from the Contribution to third party material where-ever it may be located; and, vi) licence any third party to do any or all of the above."

Funding: Combined grant MCRGS-07-16-37 from Marie Curie and the Chief Scientist Office Scotland

Competing interests: All authors have completed the ICMJE uniform disclosure form at www.icmje.org/coi_disclosure.pdf and declare: no support from any additional organisations for the submitted work; no financial relationships with any organisations that might have an interest in the submitted work in the previous three years; no other relationships or activities that could appear to have influenced the submitted work.

Approvals: Permission to access and link data from the Scottish Public Benefit and Privacy Panel for health and social care, ref 1516-0483 and approval from SE Scotland Research Ethics committee 2 reference 17/SS/0127 were gained.

Data sharing: More information may be available from the corresponding author.

Dissemination declaration: We plan to disseminate the results to patient organisations

References

1. NHS Digital. Hospital admitted patient care and adult critical care activity 2018-19. 19 Sep 2019. https://files.digital.nhs.uk/F2/E70669/hosp-epis-stat-admi-summ-rep-2018-19-rep.pdf.
2. Bone AE, Gomes B, Etkind SN, et al. What is the impact of population ageing on the future provision of end-of-life care? Population-based projections of place of death. *Palliat Med.* 2018; 32: 329-336.
3. Murtagh, F.E., et al., How many people need palliative care? A study developing and comparing methods for population-based estimates. *Palliat Med*, 2014. **28**(1): p. 49-58.
4. Institute of Medicine of the National Academies. *Dying in America: Improving Quality and Honoring Individual Preferences Near the End of Life*. The National Academies Press; Washington, DC: 2015.
5. World Health Assembly. Strengthening of palliative care as a component of comprehensive care throughout the life course. Resolution WHA67.19 adopted by the sixty-seventh World Health Assembly. Geneva: WHO, 2014.
6. World Health Organization and United Nations International Children's Fund. Declaration of Astana. Global Conference on Primary Health Care. Astana, Kazakhstan, October 25–26, 2018. Geneva and New York: WHO and UNICEF, 2018.
7. Murray SA, Kendall M, Boyd K, Sheikh A. Illness trajectories and palliative care. *BMJ*. 2005;330(7498):1007-11.

For peer review only - http://bmjopen.bmj.com/site/about/guidelines.xhtml

8. Murray SA, Kendall M, Mitchell G, Moine S, Amblàs-Novellas J, Boyd K. Palliative care from diagnosis to death. *BMJ*. 2017;356.
9. Henson, L. A., Higginson, I. J. and Gao, W. 'What factors influence emergency department visits by patients with cancer at the end of life? Analysis of a 124,030 patient cohort', *Palliat Med*, 2018. 32(2), pp. 426–438.
10. Leniz, J., Weil, A., Higginson, I. J., & Sleeman, K. E. Electronic palliative care coordination systems (EPaCCS): a systematic review. *BMJ Support & Palliat Care*, 2020. 10(1), 68 LP – 78. https://doi.org/10.1136/bmjspcare-2018-001689
11. Asaria M, Doran T, Cookson R. The costs of inequality: whole-population modelling study of lifetime inpatient hospital costs in the English National Health Service by level of neighbourhood deprivation *J Epidemiol Community Health* 2016;**70:**990-996.
12. Davies, J.M., et al., Using routine data to improve palliative and end of life care. BMJ Support *Palliat Care*, 2016. 6(3): p. 257-62.
13. Davies JM, Sleeman KE, Leniz J, Wilson R, Higginson IJ, et al. (2019) Socioeconomic position and use of healthcare in the last year of life: A systematic review and meta-analysis. PLOS Medicine 16(4): e1002782. https://doi.org/10.1371/journal.pmed.1002782
14. Ní Chróinín, D., Goldsbury, D.E., Beveridge, A. *et al.* Health-services utilisation amongst older persons during the last year of life: a population-based study. *BMC Geriatr* **18,** 317 (2018). https://doi.org/10.1186/s12877-018-1006-x
15. Walsh B, Laudicella M. Disparities in Cancer Care and Costs At The End Of Life: Evidence From England's National Health Service. *Health Aff (Millwood)*. 2017;36(7):1218-1226. doi:10.1377/hlthaff.2017.0167
16. Public Health Scotland, Patient Pathways: Emergency Care. Accessed: 24/4/2020 https://www.isdscotland.org/Health-Topics/Emergency-Care/Patient-Pathways/
17. World Health Organisation. International Statistical Classification of diseases and related health problems version 10 (ICD 10). https://www.who.int/classifications/icd/ICD10Volume2_en_2010.pdf
18. Moine, S. How to Better Visualise the Distribution of Expected Annual Deaths in a "Standard" French GP Surgery? Rebuilding the Three Main Illness Trajectories through a Modified Delphi Study. Abstract FC51. *Palliat Med* 2016. 30: NP1–NP401.
19. Scottish Government. The Scottish Index of Multiple Deprivation. Accessed: 24/4/2020 https://www2.gov.scot/Topics/Statistics/SIMD
20. Scottish Government. Scottish Government Urban Rural Classification 2016. (29 Mar 2018). Accessed 24/4/2020. https://www.gov.scot/publications/scottish-government-urban-rural-classification-2016/pages/2/
21. Tapsfield J, Hall C, Lunan C, et al. Many people in Scotland now benefit from anticipatory care before they die: an after death analysis and interviews with general practitioners. *BMJ Support Palliat Care*. 2019; 9(4):e28. doi:10.1136/bmjspcare-2015-001014.
22. Alsirafy SA, Raheem AA, Al-Zahrani AS, et al. Emergency department visits at the end of life of patients with terminal cancer: pattern, causes, and avoidability. *Am J Hosp Palliat Care* 2016. 33(7):658–662.
23. Mills S, Buchanan D, Guthrie B, et al. Factors affecting use of unscheduled care for people with advanced cancer: a retrospective cohort study in Scotland *BJGP* 2019; 69 (689): e860-e868. DOI: https://doi.org/10.3399/bjgp19X706637

For peer review only - http://bmjopen.bmj.com/site/about/guidelines.xhtml

24. Finucane AM, Davydaitis D, Horseman Z, Carduff E, et al. Electronic care coordination systems for people with advanced progressive illness: a mixed-methods evaluation in Scottish primary care. Br J Gen Pract. 2020. doi: 10.3399/bjgp19X707117
25. Smith, C., et al., Coordinate My Care: a clinical service that coordinates care, giving patients choice and improving quality of life. BMJ Support & Palliat Care, 2012. 2(4): p. 301-307.
26. Hawkes CA, Fritz Z, Deas G, et al. Development of the Recommended Summary Plan for Emergency Care and Treatment (ReSPECT), (2020). *Resuscitation*, 148: 98-107, https://doi.org/10.1016/j.resuscitation.2020.01.003.
27. Cooper, A., Carson-Stevens, A., Hughes, T., & Edwards, A. Is streaming patients in emergency departments to primary care services effective and safe? *BMJ*, 2020;368, m462. https://doi.org/10.1136/bmj.m462
28. Knaul, Felicia M et al. The Lancet Commission on Palliative Care and Pain Relief—findings, recommendations, and future directions, Volume 6, Special Issue, S5-S6, 1st March, 2018. https://doi.org/10.1016/S2214-109X(18)30082-2
29. Best S, Tate T, Noble B, et al. Research Priority Setting in Palliative and End of Life Care: The James Lind Approach Consulting Patients. *BMJ Support Palliat Care*. 2015;5(1):102 LP - 102. doi:10.1136/bmjspcare-2014-000838.

For peer review only

For peer review only - http://bmjopen.bmj.com/site/about/guidelines.xhtml

Table 1. Number (% and cumulative %) of patients (18+) in the last year of life in Scotland (2016) by number of contacts with unscheduled care services (N=56,407)

Number of service contacts	Number of patients	% Patients	Cumulative%
0	2,898	5.1	5.1
1	3,983	7.1	12.2
2	5,459	9.7	21.9
3	5,795	10.3	32.2
4	5,205	9.2	41.4
5	4,650	8.2	49.6
6	4,185	7.4	57.0
7	3,589	6.4	63.4
8	3,090	5.5	68.9
9	2,715	4.8	73.7
10	2,300	4.1	77.8
11	1,901	3.4	81.1
12	1,646	2.9	84.1
13	1,318	2.3	86.4
14	1,110	2.0	88.4
15	967	1.7	90.1
16	809	1.4	91.5
17	679	1.2	92.7
18	570	1.0	93.7
19	520	0.9	94.6
20+	3,018	5.4	100
Total	56,407	100	

For peer review only

For peer review only - http://bmjopen.bmj.com/site/about/guidelines.xhtml

Table 2. Number (and percentage) of people who had contacts with NHS telephone advice (NHS24), Primary Care Out-of-Hours (PCOOH), Scottish Ambulance Service (SAS), Emergency Department (ED), and Hospital admission for patients (18+) in the last year of life in Scotland (2016) by underlying cause of death, deprivation, place of residence, urban/rural classification (N=56,407)

		NHS24		PCOOH		SAS		ED attendance		Hospital admission	
	All deceased persons	% with contact	Number with contact	% with contact	Number with contact	% with contact	Number with contact	% with attendance	Number with attendance	% with admission	Number with admission
**Total***	56,407	59.7	33,656	53.5	30,161	73.9	41,678	62.7	35,383	74.9	42,253
**Cause of death**											
Cancer	15,902	63.4	10,074	61.5	9,783	72.8	11,569	62.0	9,857	88.3	14,039
Organ failure	21,244	56.0	11,888	45.6	9,678	78.9	16,770	65.2	13,851	73.2	15,559
Frailty	14,023	66.0	9,258	61.7	8,654	67.8	9,509	58.9	8,262	66.4	9,314
Various	2,271	56.9	1,292	48.9	1,111	76.4	1,735	64.9	1,474	74.1	1,683
External	2,967	38.5	1,142	31.5	935	70.6	2,095	65.3	1,937	55.9	1,659
p-value			<.001		<.001		<.001		<.001		<.001
**Deprivation**											
Most	13,537	59.8	8,093	46.6	6,307	77.2	10,449	67.3	9,111	76.4	10,347
2	12,812	59.8	7,665	52.5	6,720	76.9	9,850	66.5	8,517	77.2	9,892
3	11,747	59.2	6,959	55.8	6,558	72.3	8,498	60.0	7,050	73.9	8,680
4	9,840	59.8	5,883	58.8	5,790	71.5	7,032	58.7	5,775	72.9	7,177
Least	8,315	60.2	5,002	56.9	4,731	69.4	5,774	58.4	4,853	72.7	6,044
p-value			<.758		<.001		<.001		<.001		<.001
**Place of residence**											
Home/Private	47,813	58.0	27,751	50.7	24,221	77.2	36,891	65.6	31,366	79.3	37,907
Institution	8,558	68.8	5,891	69.3	5,928	55.7	4,770	46.7	4,000	50.6	4,328
p-value			<.001		<.001		<.001		<.001		<.001
**Urban/rural**											
Large urban areas	18,750	60.6	11,359	49.0	9,183	74.9	14,053	63.3	11,868	75.4	14,141
Other urban areas	20,372	61.4	12,505	53.9	10,986	75.0	15,270	66.8	13,612	75.6	15,407
Accessible small towns	5,244	57.7	3,026	55.9	2,934	74.1	3,884	62.9	3,297	74.9	3,926
Remote small towns	2,367	56.3	1,332	59.3	1,404	71.6	1,694	54.8	1,296	72.7	1,721
Accessible Rural	5,989	59.6	3,570	58.9	3,526	71.9	4,304	59.1	3,542	74.3	4,447

For peer review only - http://bmjopen.bmj.com/site/about/guidelines.xhtml

Remote Rural	3,529	51.3	1,810	58.7	2,073	68.0	2,398	47.9	1,691	70.8	2,498
p-value			<.001		<.001		<.001		<.001		<.001

* The deprivation, place of residence and urban/rural categories do not add up to 56,407 due to incomplete data. Each of these categories has less than 0.3% missing data.

For peer review only

For peer review only - http://bmjopen.bmj.com/site/about/guidelines.xhtml

Table 3. Adjusted odds ratios (OR) and 95% confidence intervals for the probability of contacts with : NHS telephone advice (NHS24), Primary Care Out-of-Hours (PCOOH), Scottish Ambulance Service (SAS), Emergency Department (ED) and Hospital Admission (HA) for patients (18+) in their last year of their life (N=56,112)*

	NHS24	PCOOH	SAS	ED	HA
	OR (95%CI)	OR (95%CI)	OR (95%CI)	OR (95%CI)	OR (95%CI)
**Sex**					
Male	1	1	1	1	1
Female	1.17 (1.12-1.21)	1.14 (1.10-1.19)	0.93 (0.88-0.96)	0.93 (0.90-0.97)	1.06 (1.02-1.11)
**Age**					
Age 18-64	1	1	1	1	1
Age 65-84	1.22 (1.16-1.28)	1.18 (1.12-1.24)	1.42 (1.34-1.50)	1.17 (1.11-1.23)	1.68 (1.58-1.78)
Age 85+	1.61 (1.51-1.71)	1.61 (1.52-1.71)	1.43 (1.34-1.53)	1.12 (1.05-1.19)	1.67 (1.56-1.79)
**Marital status**					
Single	1	1	1	1	1
Married	1.20 (1.14-1.27)	1.24 (1.17-1.31)	1.13 (1.06-1.20)	1.22 (1.15-1.30)	1.38 (1.29-1.47)
Widowed	1.27 (1.20-1.35)	1.28 (1.21-1.36)	1.13 (1.06-1.21)	1.24 (1.16-1.31)	1.36 (1.27-1.46)
Divorced	1.11 (1.04-1.19)	1.12 (1.04-1.20)	1.11 (1.03-1.20)	1.14 (1.06-1.22)	1.28 (1.19-1.39)
**Deprivation**					
Most Deprived	1.16 (1.09-1.23)	0.82 (0.78-0.87)	1.42 (1.33-1.51)	1.39 (1.31-1.48)	1.35 (1.26-1.44)
Q2	1.13 (1.06-1.19)	0.94 (0.94-0.95)	1.39 (1.31-1.49)	1.35 (1.27-1.43)	1.32 (1.23-1.41)
Q3	1.10 (1.04-1.17)	0.96 (0.96-0.97)	1.20 (1.13-1.28)	1.16 (1.09-1.23)	1.17 (1.09-1.25)
Q4	1.08 (1.01-1.15)	1.05 (1.05-1.05)	1.17 (1.10-1.25)	1.11 (1.04-1.18)	1.11 (1.04-1.19)
Least deprived	1	1	1	1	1
**Cause of death**					
Organ failure	1	1	1	1	1
Cancer	1.45 (1.39-1.52)	2.08 (1.99-2.17)	0.67 (0.64-0.70)	0.81 (0.78-0.85)	2.56 (2.28-2.86)
Frailty	1.27 (1.21-1.33)	1.44 (1.38-1.51)	0.71 (0.64-0.79)	0.77 (0.69-0.85)	0.93 (0.88-0.97)
**Urban/rural**					
Large urban areas	1.53 (1.42-1.65)	0.72 (0.67-0.78)	1.47 (1.35-1.60)	1.92 (1.77-2.07)	1.34 (1.23-1.46)
Other urban areas	1.55 (1.44-1.67)	0.88 (0.84-0.91)	1.43 (1.32-1.55)	2.24 (2.08-2.42)	1.35 (1.24-1.47)
Accessible small towns	1.33 (1.22-1.45)	0.93 (0.91-0.94)	1.38 (1.25-1.52)	1.90 (1.74-2.08)	1.26 (1.14-1.40)
Remote small towns	1.22 (1.10-1.36)	1.03 (1.04-1.02)	1.23 (1.09-1.38)	1.36 (1.22-1.51)	1.16 (1.03-1.31)
Accessible rural	1.44 (1.32-1.57)	1.01 (0.99-1.02)	1.22 (1.11-1.34)	1.60 (1.47-1.75)	1.15 (1.04-1.26)
Remote rural	1	1	1	1	1
**Place of residence**					
Institution	1	1	1	1	1
Home	1.31 (1.24-1.39)	1.89 (1.79-1.99)	2.72 (2.58-2.87)	2.33 (2.21-2.45)	3.39 (3.21-3.57)

Reference categories are male, age18-64, single, least deprived, organ failure, remote rural and institution (place of residence).

*295 patients in "Other/Unknown" categories excluded. "Various" and "External" causes of death not shown in table.

For peer review only - http://bmjopen.bmj.com/site/about/guidelines.xhtml

Table 4. Twenty most frequent Continuous Unscheduled Pathways (CUPs) by in-hours or out-of-hours start time for patients (18+) in the last year of life in Scotland in 2016 (N=56,407)

	In-hours CUPs (n=58,157)				Out-of-hours CUPs (n=133,980)			
Rank	Name	Number	%	CUM %	Name	Number	%	CUM %
1	SEA	13,702	23.6	23.6	NO	30,434	22.7	22.7
2	SA	8,639	14.9	38.5	O	18,609	13.9	36.6
3	EA	6,408	11.0	49.5	SEA	9,457	7.1	43.7
4	S	6,014	10.3	59.8	N	8,868	6.6	50.3
5	E	5,085	8.7	68.5	EA	4,923	3.7	54.0
6	SE	3,291	5.7	74.2	NSEA	4,866	3.6	57.6
7	NO	1,450	2.5	76.7	S	4,028	3.0	60.6
8	N	1,230	2.1	78.8	E	3,890	2.9	63.5
9	O	1,005	1.7	80.5	SE	2,816	2.1	65.6
10	NSEA	561	1.0	81.5	NOSEA	2,743	2.0	67.6
11	SEAS	484	.80	82.3	OO	2,697	2.0	69.6
12	SAO	366	.60	82.9	NOSA	2,306	1.7	71.3
13	SAS	305	.50	83.4	NONO	1,677	1.3	72.6
14	SEAO	305	.50	83.9	NON	1,517	1.1	73.7
15	SSEA	285	.50	84.4	NSE	1,305	1.0	74.7
16	SSA	239	.40	84.8	NOO	1,162	.90	75.6
17	SS	229	.40	85.2	NOA	1,112	.80	76.4
18	EAS	221	.40	85.6	SA	1,042	.80	77.2
19	SES	220	.40	86.0	NS	948	.70	77.9
20	NSE	164	.30	86.3	NOEA	924	.70	78.6

* 14,704 missing time stamps

Abbreviations: N=NHS 24, O= Primary Care Out-of-Hours, S=Scottish Ambulance Service, E=Emergency Department, A= Acute hospital admission

For example, the CUP “SEA” represents a contact with the Scottish Ambulance Service (S), followed by an attendance at an emergency department (E), then an unscheduled hospital admission (A).

For peer review only

For peer review only - http://bmjopen.bmj.com/site/about/guidelines.xhtml

Table 5. Mean number of contacts and costs of NHS telephone advice (NHS24), Primary Care Out-of-Hours (PCOOH), Scottish Ambulance Service (SAS), Emergency Department (ED), and Hospital admissions for patients (18+) in the last year of life in Scotland (2016) by cause of death and deprivation status (N=56,407)

	NHS24			PCOOH			SAS			Emergency department			Hospital Admissions			**All contacts**	
	Mean number of contacts	Total costs (x1000)	Costs per patient	Mean number of contacts	Total costs (x1000)	Costs per patient	Mean number of calls	Total costs (x1000)	Costs per patient	Mean number of attendances	Total costs (x1000)	Costs per patient	Mean number of admissions	Total costs (x1000)	Costs per patient	**Total costs to NHS (x1000)**	**Total cost to NHS per patient**
**Total**	1.4	£2,944	£52	1.3	£4,471	£79	1.7	£22,337	£396	1.3	£10,205	£181	1.7	£149,967	£2,659	**£189,923**	**£3,367**
**Cause of death**																	
Cancer	1.4	£836	£53	1.6	£1,502	£94	1.6	£5,807	£365	1.3	£2,765	£174	2.1	£54,022	£3,397	**£64,933**	**£4,083**
Organ failure	1.3	£1,047	£49	1.1	£1,370	£64	2.0	£9,525	£448	1.4	£4,173	£196	1.7	£56,730	£2,670	**£72,845**	**£3,429**
Frailty	1.7	£842	£60	1.6	£1,315	£94	1.5	£4,892	£349	1.1	£2,165	£154	1.3	£28,003	£1,997	**£37,218**	**£2,654**
**Deprivation**																	
Most Deprived	1.5	£748	£55	1.1	£794	£59	1.9	£5,960	£440	1.5	£2,867	£212	1.8	£38,809	£2,867	**£49,177**	**£3,633**
2	1.5	£678	£53	1.3	£984	£77	1.9	£5,543	£433	1.4	£2,559	£200	1.8	£35,805	£2,795	**£45,569**	**£3,557**
3	1.4	£607	£52	1.5	£1,048	£89	1.6	£4,390	£374	1.2	£1,936	£165	1.6	£29,625	£2,522	**£37,606**	**£3,201**
4	1.4	£501	£51	1.6	£929	£94	1.6	£3,644	£370	1.2	£1,569	£159	1.6	£25,050	£2,546	**£31,693**	**£3,221**
Least deprived	1.4	£405	£49	1.4	£710	£85	1.5	£2,766	£333	1.1	£1,256	£151	1.5	£20,349	£2,447	**£25,487**	**£3,065**

For peer review only

For peer review only - http://bmjopen.bmj.com/site/about/guidelines.xhtml

Figure 1 – Start and end points of all unscheduled care pathways

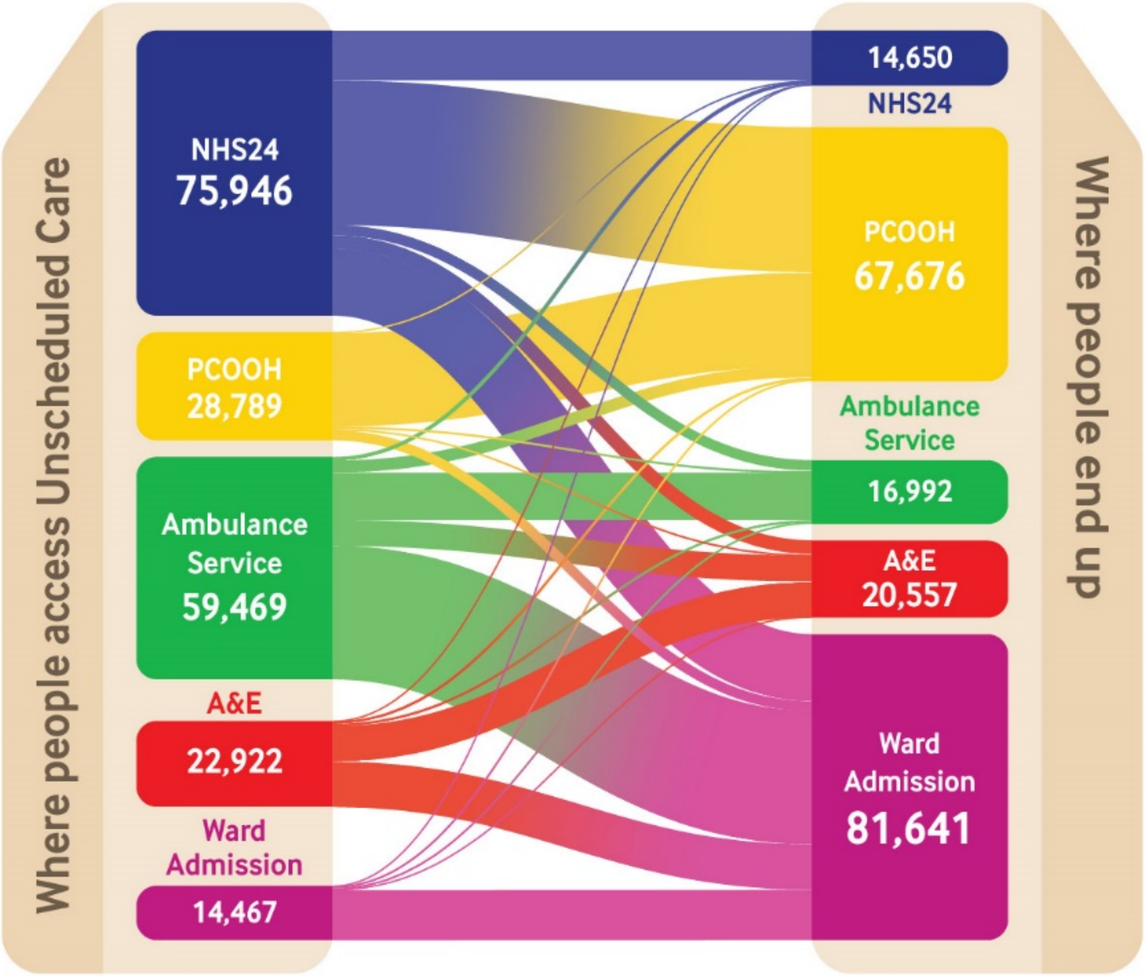

This figure shows the first and final service contacted in each Continuous Unscheduled Care Pathway (CUP). A first contact of telephone advice (NHS24) or Primary Care out-of-hours (PCOOH) was likely to be resolved in primary care. Calling an ambulance or attendance at emergency department (A&E) usually resulted in admission.

- NHS24 = telephone advice
- PCOOH= Primary Care Out of Hours
- A&E=Emergency Department

Note The figure shows 14,467 direct admissions to hospital which were likely to have been via primary care in-hours or outpatient services. Some may also be due to other direct admission routes such as the Scottish Cancer helpline for people receiving cancer treatments.

Supplementary tables 1-4

Supplementary table 1. List of ICD10 codes for underlying causes of death grouped into three main categories of conditions with potential palliative care needs (referred to as "illness trajectories")

**Illness trajectories [ITs]**	Sub-ITs	ICD10 codes (v.2010)
**IT1. Cancer *("Clear terminal phase")***		
	1.1 Malignant Neoplasms	C00-C97
**IT2. Organ failure *("Intermittent")***		
	2.1 Endocrine and metabolic diseases	E00-E90<br>*<u>Except</u>: E46*
	2.2 Diseases of the circulatory system	I00-I99<br>*<u>Except</u>: I46*<br>*(I60-I67)$_{[65+]}$****(1/3)**[1]<br>*I69*
	2.3 Diseases of the respiratory system	J00-J99.8<br>*<u>Except</u>: (J06, J09-J16, J18)$_{[65+]}$****(1/3)**[2]<br>*(J20-J22)$_{[65+]}$****(1/3)**[3]<br>*J69.0, J96*
	2.4 Diseases of liver	K70-K77, B18
	2.5 Kidney disease	N17, N18, N28
	2.6 Diseases of the digestive system	K00-K93.8<br>*<u>Except</u>: K70-K77*
	2.7 Other diseases of the genitourinary system	N00-N99.9<br>*<u>Except</u>: N17, N18, N28*
**IT3a. Frailty, Progressive neurological condition [80+]**		

For peer review only

("Gradually dwindling")

	3.1 Dementia and mental disorders [80+]	A81.0, F01, F03-F07, F09, G30, G31
	3.2 Progressive neurological diseases [80+]	G10, G12.2, G20, G35, G23.1, G90.3
	3.3 Cerebrovascular diseases [80+]	$(I60\text{-}I67)_{[80+]}$ ***(1/3)**, I69
	3.4 Some ill-defined causes [80+]	R26, R29.6, R53, R54, R55, R63, R64
		I46, J96, R96, R98, R99, W00, W03-08, W10, W18
	3.5 Malnutrition [80+]	E46
	3.6 Infectious causes / Influenza / Pneumonia [80+]	A15-A19.9, B90-B90.9, B20-B24<br>$(J06, J09\text{-}J16, J18)_{[80+]}$ ***(1/3)**<br>$(J20\text{-}J22)_{[80+]}$ ***(1/3)**, J69.0
	3.7 Skin and musculo-skeletal system [80+]	L00-L99.8, M00-M99.9
**IT3b. Frailty, Progressive neurological condition [65-79]**<br>***("Gradually dwindling")***		
	3.8 Dementia and mental disorders [65-79]	A81.0, F01, F03-F07, F09, G30, G31
	3.9 Progressive neurological diseases [65-79]	G10, G12.2, G20, G35, G23.1, G90.3
	3.10 Cerebrovascular diseases [65-79]	$(I60\text{-}I67)_{[65\text{-}79]}$ ***(1/3)**, I69
	3.11 Some ill-defined causes [65-79]	R26, R29.6, R53, R54, R55, R63, R64, I46, J96
	3.12 Malnutrition [65-79]	E46
	3.13 Infectious causes / Influenza / Pneumonia [65-79]	A15-A19.9, B90-B90.9, B20-B24<br>$(J06, J09\text{-}J16, J18)_{[65\text{-}79]}$ ***(1/3)**<br>$(J20\text{-}J22)_{[65\text{-}79]}$ ***(1/3)**<br>J69.0
	3.14 Skin and musculo-skeletal system [65-79]	L00-L99.8, M00-M99.9

For peer review only

IT3c. Frailty, Progressive neurological condition [18-64]

("Gradually dwindling")

	3.15 Dementia and mental disorders [18-64]	A81.0, F01, F03-F07, F09, G30, G31
	3.16 Progressive neurological diseases [18-64]	G10, G12.2, G20, G35, G23.1, G90.3
	3.17 Cerebrovascular diseases [18-64]	I69
	3.18 Some ill-defined causes [18-64]	R26, R29.6, R53, R54, R55, R63, R64, I46, J96
	3.19 Malnutrition [18-64]	E46
	3.20 Infectious causes / Influenza / Pneumonia [18-64]	A15-A19.9, B90-B90.9, B20-B24, J69.0
	3.21 Skin and musculo-skeletal system [18-64]	L00-L99.8, M00-M99.9

4. Various causes

	4.1 Infectious diseases	A00-B99.9<br>*Except: A81.0, A15-A19.9, B18, B20-B24, B90-B90.9*
	4.2 Neoplasms + Diseases of the blood	D01-D89.9
	4.3 Diseases of the nervous system - *except progressive neurological diseases*	G00-H95.9<br>*Except: G10, G12.2, G20, G23.1, G30, G31, G35, G90.3*
	4.4 Symptoms, signs... not elsewhere classified / Ill-defined causes - *except R26, R29.6, R53, R54, R55, R63, R64, and (R96, R98, R99)[80+]*	R00-R99<br>*Except: R26, R29.6, R53, R54, R55, R63, R64, (R96, R98, R99)[80+]*
	4.5 Substance abuse and other mental and behavioral disorders	F10-F98
	4.7 Pregnancy, childbirth, puerperium / Congenital malformation	O00-O998, P00-P96.9, Q00-Q99.9

5. External causes

5. External causes of morbidity and mortality	V01-Y89.9 *Except:(W00, W03-08, W10, W18)[80+]*	

---

[1] One third of the annual deaths with ICD-10 codes (I60-I67) as underlying cause of death (people over 65)

[2] One third of the annual deaths with ICD-10 codes (J06, J09-J16, J18) as underlying cause of death (people over 65)

[3] One third of the annual deaths with ICD-10 codes (J20-J22) as underlying cause of death (people over 65)

The characteristics of the main disease responsible for the death of a patient may be associated with certain health services utilisation patterns in the last months of life. Rather than using every single disease used for underlying causes of death in the International Statistical Classification of Diseases and Related Health Problems ("ICD-10"), we used categories of life-limiting conditions that have been described as having similar courses until death.

The conditions were grouped into three "illness trajectories" ("cancer", "organ failure", "frailty/ progressive neurological conditions"). These trajectories have a shape and an average duration, and they are associated with potential palliative care needs in the people they affect (whether physically, psychologically, socially, existentially or spiritually).

Our goal for this study was to allocate ICD-10 codes used for underlying causes of death in mortality datasets, to these three main categories of conditions ("illness trajectories").

The underlying cause of death is defined as the disease or injury which initiated the chain of events leading directly to death. Every death has just one underlying cause, so is counted only once in figures which are produced on this basis. Deaths are coded in accordance with the ICD-10.

The allocation of ICD-10 codes for underlying causes of death to the three main illness trajectories was completed prior to this study by a panel of international experts in primary care, palliative care, public health and demography, as part of a modified e-Delphi study carried out by two authors of this article (SAM and SM). The panel did not consider contributory causes of death. The list of ICD-10 codes used in this current article has been obtained after two Delphi rounds, and it consists in seven categories (as displayed in Table 1):

1. Cancer
2. Organ failure
3. Frailty/ Progressive neurological conditions [80+]
4. Frailty/ Progressive neurological conditions [65-79]
5. Frailty/ Progressive neurological conditions [18-64]
6. Various causes
7. External causes

The allocation of the total number of deaths to each main category (Scotland, 2016) is featured in Supplementary table 3

For peer review only

Supplementary table 2. Price weight allocation for cost analysis.

We allocated a fixed value for each service used based on average values.

Unscheduled Service	Value	Unit	Notes/Sources
***N**: NHS24 CONTACTS*			
NHS 24 Contact	£30.78	per call	Private communication with NHS24.
***O**: OOH PRIMARY CARE CONSULTATIONS*			
GP OOH	£38.00	per consultation	[note 1]
Nurse OOH	£6.80	per consultation	£0.70 per min x 9.72 mins. [note 1]
OOH Average Unspecified "O"	£33.20	per consultation	Weighted average of GP/Nurse consultation applied where GP/Nurse consultations were pooled. Based on ratio observed in data.
***S**: SCOTTISH AMBULANCE SERVICE (SAS)*			
Conveyed	£238.00	per trip	Applied to a S with an E or A in the CUP [note 2]
Not Conveyed	£184.00	per trip	Applied to a S without an E or A in the CUP [note 2]
OOH Average Unspecified "S"	£228.89	per trip	Weighted average of conveyed/not conveyed based on observed ratio used. Applied where A or E presence is unspecified.
***E**: EMERGENCY DEPT*			
Accident and Emergency Visit	£138.00	per visit	[note 3]
***A**: INPATIENT ADMISSIONS*			
Emergency Inpatient Admissions	£1,590.00	Per Admission	Average non-elective inpatient admission (excluding excess bed days) [note 3].

Notes

Two factors made costing emergency inpatient admissions for this cohort difficult. Firstly, the Scottish data does not include detailed costs for patient procedures we took a general population average cost. Secondly, we did not cost according to length of stay. It is possible that people who died from different underlying causes may have required different average lengths of stay. Some of the cohort may have required longer stays than patients from the general population and possibly more expensive or multiple interventions than the general population. Although the precise figures are uncertain, it is undoubtedly true that any hospital admission is an order of magnitude more expensive than any other unscheduled care service.

1. Curtis L et al. Unit Costs of Health & Social Care 2017. Kent: Personal Social Services Research Unit 2017
2. Hobbs R, Bankhead C, Mukhtar T, Stevens S, Perera-Salazar R, Holt T, & Salisbury C. (2016) Clinical workload in UK primary care: a retrospective analysis of 100 million consultations in England, 2007-14, The Lancet, 387, 10035, 2323-2330. http://www.sciencedirect.com/science/article/pii/S0140673616006206.
3. Department of Health, The. Reference Costs 2016-17 Publication. London: The Department of Health 2017

Supplementary table 3

For peer review only

Supplementary table 3. Number (%) of patients (18+) in the last year of life in Scotland 2016 by underlying cause of death (N=56,407)

Underlying cause of death	N	%
**Cancer (all types)**	**15,902**	**28.2%**
**Organ failure**	**21,244**	**37.7%**
2.1 Endocrine and metabolic	1,137	
2.2 Circulatory	11,212	
2.3 Respiratory	4,744	
2.4 Liver	1,109	
2.5 Kidney	241	
2.6 Digestive	1,949	
2.7 Other genitourinary	852	
**Frailty 80+**	**10,497**	**18.6%**
3.1 Dementia 80+	4,857	
3.2 Progressive neurological 80+	360	
3.3 Cerebrovascular 80+	2,739	
3.4 Ill-defined 80+	258	
3.4 Malnutrition 80+	3	
3.6 Infections & Pneumonia 80+	1,971	
3.7 Skin & Musculo-skeletal 80+	309	
**Frailty 65-79**	**3,076**	**5.5%**
3.8 Dementia 65-79	932	
3.9 Progressive neurological 65-79	332	
3.10 Cerebrovascular 65-79	1,042	
3.11 Ill-defined 65-79	14	
3.12 Malnutrition 65-79	3	
3.13 Infections & Pneumonia 65-79	572	
3.14 Skin & Musculo-skeletal 65-79	181	
**Frailty 19-64**	**450**	**0.8%**
3.15 Dementia 19-64	55	
3.16 Progressive neurological 19-64	180	
3.17 Cerebrovascular 19-64	50	
3.18 Ill-defined 19-64	16	
3.19 Malnutrition 19-64	2	
3.20 Infections & Pneumonia 19-64	63	
3.21 Skin & Musculo-skeletal 19-64	84	
**Various**	**2,271**	**4.0%**
4.1 Infections	648	
4.2 Neoplasms & blood	456	
4.3 Nervous system	395	
4.4 Symptoms & Signs NEC	235	
4.5 Mental & behavioural	419	
4.7 Pregnancy & congenital	118	
**External**	**2,967**	**5.3%**

For peer review only

**Total**	**56,407**	**100.0%**

Supplementary Table 4. Number (%) of contacts with services in each of the 12 months before death by patients (18+) in Scotland 2016 (Month 12 represents the 12th month before death)

	Total		Contact NHS 24		Contact PCOOH		Call to SAS		ED attendance		Emergency admissions	
Months	Number	%	Number	%	Number	%	Number	%	Number	%	Number	%
12	18,385	3.9	4,109	4.3	3,584	3.3	3,602	3.8	3,632	4.5	3,458	3.8
11	19,327	4.1	4,200	4.4	3,687	3.4	3,811	4.0	3,919	4.8	3,710	4.0
10	19,779	4.2	4,374	4.6	3,889	3.6	3,866	4.1	3,942	4.9	3,708	4.0
9	21,136	4.5	4,679	4.9	3,993	3.7	4,056	4.3	4,236	5.2	4,172	4.5
8	22,374	4.7	4,818	5.1	4,306	3.9	4,375	4.6	4,353	5.4	4,522	4.9
7	24,006	5.1	5,164	5.4	4,681	4.3	4,667	4.9	4,625	5.7	4,869	5.3
6	25,542	5.4	5,391	5.7	5,022	4.6	5,102	5.4	4,832	6.0	5,195	5.7
5	28,759	6.1	5,902	6.2	5,652	5.2	5,728	6.0	5,463	6.7	6,014	6.6
4	33,334	7.1	6,579	6.9	6,624	6.1	6,725	7.1	6,250	7.7	7,156	7.8
3	41,634	8.8	7,955	8.3	8,319	7.6	8,616	9.1	7,550	9.3	9,194	10.0
2	56,473	12.0	10,428	10.9	11,567	10.6	11,888	12.5	9,781	12.1	12,809	14.0
1	161,611	34.2	31,751	33.3	47,833	43.8	32,583	34.3	22,488	27.7	26,956	29.4
Total	472,360	100	95,350	100	109,157	100	95,019	100	81,071	100	91,763	100

For peer review only

STROBE (Strengthening The Reporting of OBservational Studies in Epidemiology) Checklist

A checklist of items that should be included in reports of observational studies. You must report the page number in your manuscript where you consider each of the items listed in this checklist. If you have not included this information, either revise your manuscript accordingly before submitting or note N/A.

Note: An Explanation and Elaboration article discusses each checklist item and gives methodological background and published examples of transparent reporting. The STROBE checklist is best used in conjunction with this article (freely available on the Web sites of PLoS Medicine at http://www.plosmedicine.org/, Annals of Internal Medicine at http://www.annals.org/, and Epidemiology at http://www.epidem.com/). Information on the STROBE Initiative is available at www.strobe-statement.org.

Section and Item	Item No.	Recommendation	Reported on Page No.
**Title and Abstract**	1	(*a*) Indicate the study's design with a commonly used term in the title or the abstract	
		(*b*) Provide in the abstract an informative and balanced summary of what was done and what was found	
**Introduction**			
Background/Rationale	2	Explain the scientific background and rationale for the investigation being reported	
Objectives	3	State specific objectives, including any prespecified hypotheses	
**Methods**			
Study Design	4	Present key elements of study design early in the paper	
Setting	5	Describe the setting, locations, and relevant dates, including periods of recruitment, exposure, follow-up, and data collection	
Participants	6	(*a*) *Cohort study*—Give the eligibility criteria, and the sources and methods of selection of participants. Describe methods of follow-up<br>*Case-control study*—Give the eligibility criteria, and the sources and methods of case ascertainment and control selection. Give the rationale for the choice of cases and controls<br>*Cross-sectional study*—Give the eligibility criteria, and the sources and methods of selection of participants	
		(*b*) *Cohort study*—For matched studies, give matching criteria and number of exposed and unexposed<br>*Case-control study*—For matched studies, give matching criteria and the number of controls per case	
Variables	7	Clearly define all outcomes, exposures, predictors, potential confounders, and effect modifiers. Give diagnostic criteria, if applicable	

For peer review only

Section and Item	Item No.	Recommendation	Reported on Page No.
Data Sources/ Measurement	8*	For each variable of interest, give sources of data and details of methods of assessment (measurement). Describe comparability of assessment methods if there is more than one group	
Bias	9	Describe any efforts to address potential sources of bias	
Study Size	10	Explain how the study size was arrived at	
Quantitative Variables	11	Explain how quantitative variables were handled in the analyses. If applicable, describe which groupings were chosen and why	
Statistical Methods	12	(*a*) Describe all statistical methods, including those used to control for confounding	
		(*b*) Describe any methods used to examine subgroups and interactions	
		(*c*) Explain how missing data were addressed	
		(*d*) *Cohort study*—If applicable, explain how loss to follow-up was addressed<br>*Case-control study*—If applicable, explain how matching of cases and controls was addressed<br>*Cross-sectional study*—If applicable, describe analytical methods taking account of sampling strategy	
		(*e*) Describe any sensitivity analyses	
**Results**			
Participants	13*	(a) Report numbers of individuals at each stage of study—eg numbers potentially eligible, examined for eligibility, confirmed eligible, included in the study, completing follow-up, and analysed	
		(b) Give reasons for non-participation at each stage	
		(c) Consider use of a flow diagram	
Descriptive Data	14*	(a) Give characteristics of study participants (eg demographic, clinical, social) and information on exposures and potential confounders	
		(b) Indicate number of participants with missing data for each variable of interest	
		(c) *Cohort study*—Summarise follow-up time (eg, average and total amount)	
Outcome Data	15*	*Cohort study*—Report numbers of outcome events or summary measures over time	
		Case-control study—Report numbers in each exposure category, or summary measures of exposure	
		Cross-sectional study—Report numbers of outcome events or summary measures	

For peer review only

Section and Item	Item No.	Recommendation	Reported on Page No.
Main Results	16	(*a*) Give unadjusted estimates and, if applicable, confounder-adjusted estimates and their precision (eg, 95% confidence interval). Make clear which confounders were adjusted for and why they were included	
		(*b*) Report category boundaries when continuous variables were categorized	
		(*c*) If relevant, consider translating estimates of relative risk into absolute risk for a meaningful time period	
Other Analyses	17	Report other analyses done—eg analyses of subgroups and interactions, and sensitivity analyses	
**Discussion**			
Key Results	18	Summarise key results with reference to study objectives	
Limitations	19	Discuss limitations of the study, taking into account sources of potential bias or imprecision. Discuss both direction and magnitude of any potential bias	
Interpretation	20	Give a cautious overall interpretation of results considering objectives, limitations, multiplicity of analyses, results from similar studies, and other relevant evidence	
Generalisability	21	Discuss the generalisability (external validity) of the study results	
**Other Information**			
Funding	22	Give the source of funding and the role of the funders for the present study and, if applicable, for the original study on which the present article is based	

*Give information separately for cases and controls in case-control studies and, if applicable, for exposed and unexposed groups in cohort and cross-sectional studies.

Once you have completed this checklist, please save a copy and upload it as part of your submission. DO NOT include this checklist as part of the main manuscript document. It must be uploaded as a separate file.

For peer review only

BMJ Open

Unscheduled and out-of-hours care for people in their last year of life: a retrospective cohort analysis of national datasets

Journal:	*BMJ Open*
Manuscript ID	bmjopen-2020-041888.R2
Article Type:	Original research
Date Submitted by the Author:	17-Oct-2020
Complete List of Authors:	Mason, Bruce; University of Edinburgh<br>Kerssens, Joannes; Public Health Scotland, Data Driven Innovation<br>Stoddart, Andrew; University of Edinburgh,<br>Murray, Scott; The University of Edinburgh Edinburgh Medical School, Primary Pallaitive Care Research Group, The Usher Institute,<br>Moine, Sébastien ; University of Edinburgh, 3. Primary Palliative Care Research Group, Usher Institute of Population Health Sciences and Informatics; University of Paris 13, Health Education and Practices Laboratory<br>Finucane, Anne; Marie Curie Hospice Edinburgh<br>Boyd, Kirsty; University of Edinburgh, Primary Palliative Care Research Group
**Primary Subject Heading**:	Health services research
Secondary Subject Heading:	Emergency medicine, General practice / Family practice, Palliative care, Public health
Keywords:	PRIMARY CARE, ACCIDENT & EMERGENCY MEDICINE, Adult palliative care < PALLIATIVE CARE, HEALTH SERVICES ADMINISTRATION & MANAGEMENT, PUBLIC HEALTH, STATISTICS & RESEARCH METHODS

SCHOLARONE™
Manuscripts

I, the Submitting Author has the right to grant and does grant on behalf of all authors of the Work (as defined in the below author licence), an exclusive licence and/or a non-exclusive licence for contributions from authors who are: i) UK Crown employees; ii) where BMJ has agreed a CC-BY licence shall apply, and/or iii) in accordance with the terms applicable for US Federal Government officers or employees acting as part of their official duties; on a worldwide, perpetual, irrevocable, royalty-free basis to BMJ Publishing Group Ltd ("BMJ") its licensees and where the relevant Journal is co-owned by BMJ to the co-owners of the Journal, to publish the Work in this journal and any other BMJ products and to exploit all rights, as set out in our licence.

The Submitting Author accepts and understands that any supply made under these terms is made by BMJ to the Submitting Author unless you are acting as an employee on behalf of your employer or a postgraduate student of an affiliated institution which is paying any applicable article publishing charge ("APC") for Open Access articles. Where the Submitting Author wishes to make the Work available on an Open Access basis (and intends to pay the relevant APC), the terms of reuse of such Open Access shall be governed by a Creative Commons licence – details of these licences and which Creative Commons licence will apply to this Work are set out in our licence referred to above.

Other than as permitted in any relevant BMJ Author's Self Archiving Policies, I confirm this Work has not been accepted for publication elsewhere, is not being considered for publication elsewhere and does not duplicate material already published. I confirm all authors consent to publication of this Work and authorise the granting of this licence.

For peer review only

Unscheduled and out-of-hours care for people in their last year of life: a retrospective cohort analysis of national datasets

Bruce Mason, Joannes Kerssens, Andrew Stoddart, Scott A. Murray, Sebastien Moine, Anne M. Finucane, Kirsty Boyd

Primary Palliative Care Research Group, Usher Institute, University of Edinburgh, EH8 9AG Scotland, UK Bruce Mason, senior researcher

Electronic Data Research and Innovation Service, Public Health Scotland, Little France Road, Edinburgh, EH16 4UX Scotland, UK Joannes Kerssens, principal information analyst and statistician

Edinburgh Clinical Trials Unit, Usher Institute, University of Edinburgh, Scotland, EH8 9AG UK Andrew Stoddart, senior health economist[3]

Primary Palliative Care Research Group, Usher Institute, University of Edinburgh, Scotland, EH8 9AG UK Scott A. Murray, emeritus professor[1]

Health Education and Practices Laboratory, University of Paris 13, Bobigny, France. Sebastien Moine, senior visiting fellow at Usher Institute, Edinburgh, UK

Marie Curie Hospice, 45 Frogston Road West, Edinburgh, EH10 7DR, Scotland, UK. Anne M. Finucane, research lead

Primary Palliative Care Research Group, Usher Institute, University of Edinburgh, Scotland, EH8 9AG UK Kirsty Boyd, reader

Correspondence to Scott A Murray, Primary Palliative Care Research Group, Usher Institute, University of Edinburgh, Scotland, EH8 9AG UK Scott.Murray@ed.ac.uk

Key words: Out-of-hours care, unscheduled care, routine data, primary care, care pathway

For peer review only - http://bmjopen.bmj.com/site/about/guidelines.xhtml

Summary:

Objectives To analyse patterns of use and costs of unscheduled NHS services for people in the last year of life.

Design Retrospective cohort analysis of national datasets with application of standard UK costings

Participants and setting All people who died in Scotland in 2016 aged 18 or over (n= 56,407)

Main outcome measures Frequency of use of the five unscheduled NHS services in the last 12 months of life by underlying cause of death; patient demographics; Continuous Unscheduled Care pathways (CUPs) followed by patients during each care episode; total NHS and per-patient costs.

Results 53,509 patients (94.9%) had at least one contact with an unscheduled care service during their last year of life (472,360 contacts) with 34.2% in the last month of life. By linking patient contacts during each episode of care, we identified 206,841 Continuous Unscheduled Care pathways (CUPs); with 133,980 (64.8%) starting out-of-hours. People with cancer were more likely to contact the NHS telephone advice line (63%) ($X^2(4)=1004, p<.001$) or primary care out-of-hours (62%) ($X^2(4)=1924, p<.001$) and have hospital admissions(88%) ($X^2(4)=2644, p<.001$). People with organ failure (79%) contacted the ambulance service most frequently ($X^2(4)=584, p<.001$). Demographic factors associated with more unscheduled care were older age, social deprivation, living in own home, and dying of cancer. People dying with organ failure formed the largest group in the cohort and had the highest NHS costs as a group. The cost of providing services in the community was estimated at 3.9 % of total unscheduled care costs despite handling most out-of-hours calls.

Conclusions Over 90% of people used NHS unscheduled care in their last year of life. Different underlying causes of death and demographic factors impacted on initial access and subsequent pathways of care. Managing more unscheduled care episodes in the community has the potential to reduce hospital admissions and overall costs.

For peer review only - http://bmjopen.bmj.com/site/about/guidelines.xhtml

Article summary

Strengths and limitations of this study

- Linking death certification codes with service use in the 12 months before death allowed us to analyse the impact of different illnesses on unscheduled care service use.
- We linked all five unscheduled NHS services (telephone advice, primary care, ambulances, emergency department and hospital admission) into Continuous Unscheduled Pathways (CUPs) and identified common patterns of unscheduled care for people.
- This innovative, population based method provided a broad understanding of how different demographic factors affected use of unscheduled care throughout Scotland.
- Only one of the datasets, primary care out-of-hours, contained information that could indicate whether a person was identified for palliative care.
- Costs were calculated using weighted averages and were therefore approximate.

Introduction

Rising demand for unscheduled care is a major burden and causes pressure on healthcare systems internationally, both in and out-of-hours. Unscheduled care is unplanned and demand-led and free at point of access. In the UK, it includes five NHS services: telephone advice, primary care services, ambulance services, emergency department and acute hospital admission. Emergency department targets were unmet throughout the UK prior to the impact of Covid-19. Unplanned hospital admissions in the UK increased by 28% from 2010-2019, while elective admissions rose by 25% during the same time period. [1] Contributory factors include an aging population with multiple health conditions, public expectations, instructions to seek urgent care for suspected strokes or heart attacks, and less support in the community at weekends and overnight. [2] Much unscheduled care is used by people in their last year of life who are known to have significant health-related suffering and unmet palliative care needs. [3]

For peer review only - http://bmjopen.bmj.com/site/about/guidelines.xhtml

Early integration of a palliative approach as a component of chronic disease management is strongly recommended for people with advanced illnesses in all care settings. [4,5] Palliative care is a core component of universal health coverage and its people-centered ethos and focus on quality of life and death should be considered at every opportunity. [6] Services should be designed to respond to the typical trajectories of declining health of patients with all progressive illnesses, including social and psychological factors. [7, 8] Palliative care includes proactive care planning which reduces burdensome interventions of low benefit, and helps avoid some unwarranted hospital admissions. However, palliative care integration into unscheduled care services has proven particularly challenging. [9, 10]

National datasets can be used to monitor and improve care. They have underutilised potential to improve end-of-life care. [11-15] In Scotland, death registry data, and activity data from hospitals, the ambulance service, and out-of-hours primary care services are collected routinely. These national datasets contain a unique identifier—the Community Health Index (CHI). We set out to link these data to analyse patterns of unscheduled care services use and costs by underlying cause of death and patient demographics.

Methods

We linked three datasets. The *National Records of Scotland (NRS) deaths dataset* was used to identify all adults (aged 18+) who died in Scotland in 2016. From this dataset we extracted underlying cause of death and usual place of residence. From the General Acute Inpatient and Day Case - Scottish Morbidity Record (SMR01) we extracted all unscheduled hospital activity for the last 12 months life for the cohort. Thirdly, we extracted data from the *Scottish Unscheduled Care Datamart* (UCD) covering four unscheduled care services: the telephone advice line (NHS24), primary care out-of-hours (PCOOH), the Scottish ambulance service (SAS), and emergency department attendances (ED). [16] The UCD does not include in-hours, unscheduled primary care.

For peer review only - http://bmjopen.bmj.com/site/about/guidelines.xhtml

The ICD codes (v 2010) for underlying cause of death extracted from the NRS dataset were classified into five groups: cancer, organ failure, frailty/progressive neurological conditions, various other causes, and external causes.[17] People in the first three groups were considered potentially to have had palliative care needs during their last year of life. Details of this coding allocation had been agreed previously by an expert international panel. [18] (See online supplementary Table 1)

Postcodes of usual place of residence were extracted from the NRS dataset. We used the Scottish Index of Multiple Deprivation (SIMD 2016) to infer quintiles of deprivation. [19] Rurality was based on the Scottish Government Urban-Rural Classification as applied to the postcodes. [20]

In order to understand how multiple services were accessed during a single healthcare episode, we used Continuous Unscheduled Pathways (CUPs) as defined in the Scottish unscheduled care datamart. A CUP is a linked set of contacts with one or more unscheduled care services. [16] Each CUP represents a single patient journey. The frequencies of the different types of CUP were tabulated to identify key patterns. We categorised a CUP as 'out-of-hours' if it started at weekends, on public holidays or on weeknights from 6pm to 8am. There is no limit to the duration of each CUP so a CUP could start out-of-hours but end during the in-hours period and vice-versa. Therefore, only the start date and time of the CUP was used to categorise it.

In the Unscheduled Care Dataset, each service component of a CUP is assigned a code letter:

- N=NHS 24
- O= Primary Care Out of Hours
- S=Scottish Ambulance Service
- E=Emergency Department
- A= Acute hospital admission

For peer review only - http://bmjopen.bmj.com/site/about/guidelines.xhtml

Linking these codes in chronological order gives the pathway its name. For example, 'NSE' represents a call to NHS 24 (N), followed by an ambulance service contact (S), then an emergency department attendance (E).

Statistical analysis: Descriptive analyses were undertaken using means and frequency tables. Service use of people with or without unscheduled care contacts were compared using chi-squared tests for categorical variables. Multiple logistic regression models analysed multivariate associations between predictor variables (gender, age, marital status, deprivation quintile, cause of death, urban/rural classification and place of residence) and the odds of using a service. All analyses were conducted within the Scottish National Safe Haven by a senior analyst (JK) after approval by a Scottish Public Benefit and Privacy Panel (PBPP).

Cost estimations: Standard UK price weighting methodology was applied to estimate the costs of each unscheduled service: see online supplementary Table 2 to explain how this was calculated. We did not attribute exact pricing to different forms of inpatient admission or account for differences due to patient demographics hence mean population values were applied. These costs are included as broad indicators of differences in scale and should not be interpreted as exact data.

Patient and Public Involvement: Representatives from Marie Curie Expert Voices Scotland and a Royal College of General Practitioners Scottish patient group joined the steering group and contributed public-patient perspectives from their own groups throughout the project. Key stakeholders from the unscheduled services and patient group members advised the research team on parameters for analysis, choice of analyses, and data interpretation. To understand decision-making and experiences of service users, we conducted focus groups and interviews with patients with advanced illnesses and carers who had used unscheduled services, and with bereaved carers. A final key stakeholder meeting of professionals, policy makers and lay representatives discussed the findings and implications for service development. These data will be reported elsewhere.

For peer review only - http://bmjopen.bmj.com/site/about/guidelines.xhtml

For peer review only

Results

1 Underlying causes of death in the cohort

We extracted records for 56,407 adults who died in Scotland in 2016 and linked records covering the last 12 months of life for each individual in this cohort. The number of people in each disease group was as follows: cancer 28.2%, organ failure 37.7%, frailty/progressive neurological conditions 24.9%, other diseases / various causes 4.0%, and external causes 5.3% (see: online supplementary Table 3).

2 Use of NHS unscheduled care services

The cohort had 472,360 unscheduled care service contacts; 56,407 people (94.9% of the cohort) had at least one contact. Table 1 shows the distribution of unscheduled service use: 50.4% had 6 or more contacts, and the 5.4% who had 20 or more contacts accounted for 21.5% of all contacts. All unscheduled care services were used increasingly as death approached, with 34.2% occurring in the last month of life (see online supplementary Table 4). During that final month, there was a disproportionate rise in primary care out-of-hours workload.

Table 2 displays the number and percentages of people who contacted the five unscheduled care services during the last 12 months of life by cause of death and demographic factors. Place of residence at death had two categories: those living in a private residence or people living in any institution. The latter were primarily care homes, but also included prisons and hostels. Due to the large sample size, all differences in Table 2 were statistically significant (except contacts with NHS 24 by deprivation).

24-hour Telephone Advice Service (NHS24)

More people dying with frailty (66.0%) or cancer (63.4%) contacted this service than those dying with organ failure (56.0%) (X^2(4,N=56,407)=1004,*p*<.001). People living in institutions (or commonly the staff caring for them) were more likely to contact NHS24 than those living at home: 68.8% v 58.0%

For peer review only - http://bmjopen.bmj.com/site/about/guidelines.xhtml

For peer review only

(X^2(1,N=56,371)=352,p<.001). People from all deprivation quintiles were about as likely to access NHS24 (X^2(4,N=56,251)=1.88,p=.758). People who lived in urban areas contacted NHS24 more than those from remote areas: 58.7 v 49.0% (X^2(5,N=56,251)=154,p<.001).

Primary Care Out-of-Hours (PCOOH)

Service use was similar to NHS24, as more people dying with cancer (61.5%) or frailty (61.7%) had contact with this service compared to those with organ failure (45.6%) (X^2(4,N=56,407)=1924,p<.001). People living in an institution were substantially more likely to have used this service than those in a private residence: 69.3% v 50.7% (X^2(1,N=56,371)=1011,p<.001). People living in the most deprived quintile were less likely to access PCOOH (46.6%) compared to those from the least deprived quintile (56.9%) (X^2(4,N=56,251)=442,p<.001).

Scottish Ambulance Service

People who died from organ failure used this service (78.9%) more than those with cancer (72.8%) or frailty (67.8%) (X^2(4,N=56,407)=584,p<.001). People living at home were much more likely to phone the ambulance service than those in an institution (77.2% v 55.7%) (X^2(1,N=56,371)=1725,p<.001). People in the most deprived quintile accessed help from the ambulance service more often (77.2%) than people in the least deprived. (69.5%) (X^2(4,N=56,251)=266,p<.001).

Emergency Department

People who died from organ failure used this service (65.2%) more than those with cancer or frailty (X^2(4,N=56,407)=190,p<.001), just as they did with the ambulance service. People living in institutions were less likely to visit an emergency department than those living at home (46.7% v 65.6%) (X^2(1,N=56,371)=1250,p<.001). Those from the most deprived quintile were more likely to attend an emergency department (67.3%) compared to people in the least deprived quintile (61.3%) (X^2(4,N=56,251)=386,p<.001).

For peer review only - http://bmjopen.bmj.com/site/about/guidelines.xhtml

Acute Hospital Admission

More people who died from cancer had at least one acute hospital admission (88.3%) compared to those with organ failure (73.2%) or frailty (66.4%) (X^2(4,N=56,407)=2644,*p*<.001). People living at home were much more likely to be admitted to hospital (79.3%) than those living in an institution (50.6%) (X^2(1,N=56,371)=3043,*p*<.001). People in the most deprived quintile were admitted more often (75.5%) than people in the least deprived. (70.8%) (X^2(4,N=56,251)=134,*p*<.001).

Patterns of use across unscheduled care services

Logistic regression modelling included the clinical, socioeconomic and location variables from Table 2 plus age, gender, and marital status. (Table 3) The odds ratio (OR) for contacts with NHS24 was higher for women than men (adjusted OR 1.17, 95% CI = 1.12-1.21). People aged 65-84 (adjusted OR 1.68, 95% CI = 1.58-1.78) and those aged 85 or over (adjusted OR 1.67, 95% CI = 1.56-1.79) had higher odds ratios for acute hospital admission compared to people aged 18-64. Comparing the three main groups of causes of death, the odds of people with cancer having a PCOOH contact in their last year of life was much greater than for people dying with organ failure (adjusted OR 2.08, 95% CI = 1.99-2.17). After controlling for demographic and location variables, individuals with cancer had a higher risk of an acute admission than people with organ failure (adjusted OR 2.56, 95% CI = 2.28-2.86). Overall, people living at home used more unscheduled care services than those in institutions. This was particularly the case for acute hospital admissions (adjusted OR 3.39, 95% CI = 3.21-3.57).

People in the most deprived quintile tended to use the three unscheduled care services that are not community based more than those in the least deprived quintile. This was especially so for ambulance services (adjusted OR 1.42, 95% CI = 1.33-1.51). Those living in urban areas used more of all the services except PCOOH than people from rural areas overall.

3. Use of Continuous Unscheduled Pathways (CUPs)

For peer review only

For peer review only - http://bmjopen.bmj.com/site/about/guidelines.xhtml

Linking the serial use of the unscheduled service contacts into CUPs enabled us to delineate common sequences and patterns of service use. Table 4 shows the 20 most common CUPs according to when they started; in-hours or out-of-hours. NHS24 and the ambulance service were the most common initial access points. The most common end points were PCOOH and hospital admission. (Figure 1) Over half the ambulance calls led to an acute hospital admission (60.0%). Conversely, 50.6% of initial calls to NHS24 were dealt with in the community by PCOOH.

Differences in frequencies and types of CUP by start time

We differentiated between CUPs that started out-of-hours or in-hours to look at implications for improving out-of-hours care as well as unscheduled care in general. We identified 206,841 continuous unscheduled pathways, of which 133,980 (64.8%) started out-of-hours, 28.1% in-hours, and 7.1% unknown (mostly due to lack of a time-stamp on acute hospital admissions). Contacts with NHS24 and PCOOH were much more frequent for out-of-hours CUPs. Data on the proportion of contacts with each service that occurred during out-of-hours CUPs were: NHS24 93.1%, PCOOH 94.7%, ambulance service 37.7%, and emergency department 44.4%.

Table 4 data allows a detailed understanding of how patients typically move through the services night and day. Only 16.7% of out-of-hours CUPs started with an ambulance call while 73.2% started with an NHS24 or PCOOH contact. Similarly, most out-of-hours CUPs ended in primary care: 9.6% with telephone advice from NHS24 and 46.7% with PCOOH. Much fewer out-of-hours CUPs resulted in an acute hospital admission (27.5%) or emergency department attendance (8.2%). In contrast, the six most common CUPs which started in-hours comprised episodes consisting of ambulance calls, emergency department visits and acute hospital admissions, and these accounted for 74.2% of all CUPs which started in-hours. GP in-hours care is not included in the UCD so was not available.

4. Costs

For peer review only - http://bmjopen.bmj.com/site/about/guidelines.xhtml

The mean number of contacts, per patient costs and total NHS costs for the five unscheduled care services in the last year of life are listed by underlying causes of death and deprivation status in Table 5. People with organ failure formed the largest group and had the highest total NHS costs as a group due to use of ambulance and hospital services. Those with frailty incurred the least unscheduled NHS care costs, being managed more in the community. The total cost of unscheduled NHS care in Scotland for people in their last year of life was nearly £190 million, of which only 3.9% was for provision of primary care services.

The total mean per patient costs of unscheduled care in the last year of life were greatest for those with cancer (£4,083), followed by organ failure (£3,429), and frailty (£2,654). Unscheduled per patient costs for people in the most deprived quintile were 18.8% higher than those from the least deprived but their PCOOH costs per capita were 30.6% lower.

Discussion

Principal findings

We found that 94.9% of people had unscheduled care contacts during their last year of life. They had a median of five contacts, with 5.1% making 20 or more, and 34.2% of all contacts occurring during the final month of life. We identified three groups of patients by underlying cause of death with different patterns of unscheduled care service use that were clinically and statistically significant. People with cancer had more unscheduled admissions than people with non-cancer diagnoses and the highest per patient costs. People who died with frailty were most likely to have unscheduled care that was managed fully in the community. People with organ failure used most ambulance services, and as a group accounted for the greatest number of acute hospital admissions overall. People from the most deprived quintile used significantly less PCOOH than those from the least deprived but they accessed the other four unscheduled services more. The total cost of service use by people in the most deprived quintile was almost double that of those in the least deprived, due to

For peer review only - http://bmjopen.bmj.com/site/about/guidelines.xhtml

the greater numbers of people dying in this group and their higher use of secondary care services. NHS24 and PCOOH services together accounted for less than 4% of total NHS unscheduled care costs.

Strengths and limitations of the study

Strengths This innovative approach to studying population use of interconnected, unscheduled care services provided a broad understanding of how different illnesses, and demographic factors affected use of unscheduled care. Linking all five unscheduled NHS services together into patient pathways (CUPs) allowed common patterns of unscheduled care to be identified and quantified. Studying the unscheduled care service pathways of people in their last year of life throughout Scotland enabled us to analyse the unscheduled care provided for a whole population which has rising numbers of people with unidentified palliative care needs.[3] Understanding the perspectives and choices made by people and their families seeking unscheduled care is equally important and was the qualitative data component of our overall study (to be reported elsewhere).

Limitations The structure and scope of unscheduled care services in Scotland influenced how those services were used so comparisons with different countries and healthcare systems will need care. Lack of national data for in-hours, unscheduled primary care was limiting. Some CUPs recorded as out-of-hours may have started with an undocumented, urgent primary care contact in-hours. Timing of acute hospital admissions is not recorded so could not be separated by starting point. Costs were calculated using weighted averages and were therefore approximate. We acknowledge limitations in relying on ICD-10 recorded diagnoses. We had intended to look for evidence that patients had been identified for palliative care. Unfortunately, only the primary care out-of-hours dataset had a palliative care code or recorded access to the Scottish electronic care plan (Key Information Summary) used by primary care teams to coordinate palliative and anticipatory care planning. [21] This meant it was impossible to estimate the full extent of proactive care planning or palliative care provision by NHS unscheduled care services.

For peer review only - http://bmjopen.bmj.com/site/about/guidelines.xhtml

Comparison with other studies

Most studies of unscheduled care have focussed on individual services and specific diseases, notably emergency departments and patients with cancer, and have suggested that only 30-35% of people with cancer use unscheduled care in the last year of life. [9, 22] However, a recent study in one Scottish region reported that 78% of people dying from cancer had unscheduled care provided by emergency departments and/or PCOOH in their last year of life. [23] Our findings, integrating all five NHS unscheduled care services, found that 94.9% of people who died in Scotland received unscheduled care in their last year of life. By using population data along with specific service use data, we have highlighted the extent and diversity of unscheduled care pathways. Our logistic regression models identified differences in unscheduled care by deprivation quintile for each of the three main illness groups, not just for the population with cancer, and correlates with other evidence around the relationship between socioeconomic status and use of emergency services. [9, 11, 13]

Meaning of the study and implications for clinicians and policymakers

Many more people seek unscheduled and out-of-hours care in their last year of life than was recognised previously. There were common patterns associated with different underlying illnesses, deprivation status and place of residence. Knowledge of how these groups of patients respond to urgent care needs may help community and hospital services find ways to respond more effectively and potentially could reduce demand for costly services. Primary care teams, social care managers and hospital teams can identify frequent or unusual patterns of contacts with unscheduled care and use these to trigger new or updated care planning. Such care planning communicated to unscheduled care services routinely via primary care managed electronic care co-ordination systems has been linked with fewer hospital admissions and deaths in Scotland. [21, 24] In London, the "Coordinate my Care" system uploads and shares urgent care plans entered by primary care, ambulance and hospital services thereby reducing hospital admissions, and electronic care planning

For peer review only

For peer review only - http://bmjopen.bmj.com/site/about/guidelines.xhtml

systems are evolving in other parts of England. [10, 25] The UK ReSPECT process partners with patients and families to make emergency treatment and care plans that can help guide unscheduled care and reduce unwarranted admissions. [26] Key aspects are early identification of people at risk of deteriorating health, proactive care planning, and a readily accessible electronic care coordination system that can be read and updated by any professional responsible for a person's care.

Improving the ability of unscheduled primary care services to manage people in the community is likely to be highly cost-effective as well as supporting people's choice to remain at home towards the end of life. Emergency departments are already employing more primary care clinicians to enhance pre-hospital triage and ambulance crews are providing more care at home, where appropriate, instead of transferring patients to hospital. [27] Interventions in the community by NHS 24, PCOOH and ambulance services have potential to provide high- value low-cost care.

Further research

Unscheduled care of the whole population merits ongoing research using population-level data, encompassing all community and hospital settings both in-hours and out-of-hours. Improving the scope and quality of data collected routinely can facilitate research into the needs of people who are high service users and stand to benefit from better coordinated care. [5, 6, 28] Specifically, research to understand specific differences in care pathways and service use is important, such as why people with cancer have more urgent hospital admissions than others and why people with organ failure call the ambulance service relatively frequently. Our data also provides a baseline that can be used in studies to evaluate changes in the use of unscheduled services during the coronavirus pandemic, when the demand for hospital-based unscheduled care dipped sharply.

Interventions to encourage a palliative care approach in each of the five out-of-hours services, as well as care coordination throughout the unscheduled pathways are recommended. [29] An evaluation of telephone advice services including emergency social care, community nurse

For peer review only - http://bmjopen.bmj.com/site/about/guidelines.xhtml

telephone support such as the Gold Line, specialist palliative care support lines, and from charities offering help for people with specific illnesses is indicated to scope provision of unscheduled care in the community further. [29] Research into the contribution of specialist palliative care out-of-hours, which has not been included in this study, and interventions to co-ordinate care between settings at the end of life are also needed. [29]

Conclusions

The extent of unscheduled care delivered to people in their last year of life is significantly greater and more varied than reported previously. People with diverse urgent care needs are accessing these services at high levels, particularly in their final month of life. More should be done to take account of underlying illness trajectories and social determinants of health, including better public understanding of how to access the right care in timely and effective ways. Systematic approaches to care planning combined with effective recording and sharing of key information, including a palliative care code where appropriate, is vital and should be recorded in routine healthcare datasets.

Figure legend

Figure 1 – Start and end points of all unscheduled care pathways

Acknowledgments:

The authors acknowledge the support of National Services Scotland for their involvement of obtaining approvals, provisioning and data linkage. We also acknowledge advice from the wider project steering group: Marilyn Kendall, senior researcher; Sheonad Laidlaw, general practitioner; Emma Carduff, honorary research fellow and Marie Curie research lead; Erna Haraldsdottir, senior lecturer; Sir Lewis Ritchie, Scottish Government advisor; Sian Tucker, NHS24 national lead; Marie Fallon, professor of palliative medicine; Jeremy Keen, consultant in palliative medicine; Stella Macpherson, patient and public involvement representative; Nicole Brun, project administrator. We also thank Bruce Guthrie and Sarah Mills for commenting on a draft of this paper.

Contributor and guarantor information: BM, SAM, SM, AF, AS and KB designed the study (along with members of the steering group acknowledged above), JK and AS linked and analysed the data, SM allocated illness trajectories to all decedents, and AF conducted literature reviews. All contributors discussed the findings and contributed to writing the drafts and the final paper. The contributors met

For peer review only - http://bmjopen.bmj.com/site/about/guidelines.xhtml

For peer review only

with the steering group regularly to design and guide the overall study and to integrate patient and carer perspectives. SAM and KB are the guarantors. The corresponding author attests that all listed authors meet authorship criteria and that no others meeting the criteria have been omitted.

Copyright /license for publication: The Corresponding Author has the right to grant on behalf of all authors and does grant on behalf of all authors, a worldwide licence to the Publishers and its licensees in perpetuity, in all forms, formats and media (whether known now or created in the future), to i) publish, reproduce, distribute, display and store the Contribution, ii) translate the Contribution into other languages, create adaptations, reprints, include within collections and create summaries, extracts and/or, abstracts of the Contribution, iii) create any other derivative work(s) based on the Contribution, iv) to exploit all subsidiary rights in the Contribution, v) the inclusion of electronic links from the Contribution to third party material where-ever it may be located; and, vi) licence any third party to do any or all of the above."

Funding: Combined grant MCRGS-07-16-37 from Marie Curie and the Chief Scientist Office Scotland

Competing interests: All authors have completed the ICMJE uniform disclosure form at www.icmje.org/coi_disclosure.pdf and declare: no support from any additional organisations for the submitted work; no financial relationships with any organisations that might have an interest in the submitted work in the previous three years; no other relationships or activities that could appear to have influenced the submitted work.

Approvals: Permission to access and link data from the Scottish Public Benefit and Privacy Panel for health and social care, ref 1516-0483 and approval from SE Scotland Research Ethics committee 2 reference 17/SS/0127 were gained.

Data sharing: More information may be available from the corresponding author.

Dissemination declaration: We plan to disseminate the results to patient organisations

References

1. NHS Digital. Hospital admitted patient care and adult critical care activity 2018-19. 19 Sep 2019. https://files.digital.nhs.uk/F2/E70669/hosp-epis-stat-admi-summ-rep-2018-19-rep.pdf.
2. Bone AE, Gomes B, Etkind SN, et al. What is the impact of population ageing on the future provision of end-of-life care? Population-based projections of place of death. *Palliat Med.* 2018; 32: 329-336.
3. Murtagh, F.E., et al., How many people need palliative care? A study developing and comparing methods for population-based estimates. *Palliat Med*, 2014. **28**(1): p. 49-58.
4. Institute of Medicine of the National Academies. *Dying in America: Improving Quality and Honoring Individual Preferences Near the End of Life*. The National Academies Press; Washington, DC: 2015.
5. World Health Assembly. Strengthening of palliative care as a component of comprehensive care throughout the life course. Resolution WHA67.19 adopted by the sixty-seventh World Health Assembly. Geneva: WHO, 2014.
6. World Health Organization and United Nations International Children's Fund. Declaration of Astana. Global Conference on Primary Health Care. Astana, Kazakhstan, October 25–26, 2018. Geneva and New York: WHO and UNICEF, 2018.

For peer review only - http://bmjopen.bmj.com/site/about/guidelines.xhtml

7. Murray SA, Kendall M, Boyd K, Sheikh A. Illness trajectories and palliative care. *BMJ*. 2005;330(7498):1007-11.
8. Murray SA, Kendall M, Mitchell G, Moine S, Amblàs-Novellas J, Boyd K. Palliative care from diagnosis to death. *BMJ*. 2017;356.
9. Henson, L. A., Higginson, I. J. and Gao, W. 'What factors influence emergency department visits by patients with cancer at the end of life? Analysis of a 124,030 patient cohort', *Palliat Med*, 2018. 32(2), pp. 426–438.
10. Leniz, J., Weil, A., Higginson, I. J., & Sleeman, K. E. Electronic palliative care coordination systems (EPaCCS): a systematic review. *BMJ Support & Palliat Care*, 2020. 10(1), 68 LP – 78. https://doi.org/10.1136/bmjspcare-2018-001689
11. Asaria M, Doran T, Cookson R. The costs of inequality: whole-population modelling study of lifetime inpatient hospital costs in the English National Health Service by level of neighbourhood deprivation *J Epidemiol Community Health* 2016;**70:**990-996.
12. Davies, J.M., et al., Using routine data to improve palliative and end of life care. BMJ Support *Palliat Care*, 2016. 6(3): p. 257-62.
13. Davies JM, Sleeman KE, Leniz J, Wilson R, Higginson IJ, et al. (2019) Socioeconomic position and use of healthcare in the last year of life: A systematic review and meta-analysis. PLOS Medicine 16(4): e1002782. https://doi.org/10.1371/journal.pmed.1002782
14. Ní Chróinín, D., Goldsbury, D.E., Beveridge, A. *et al.* Health-services utilisation amongst older persons during the last year of life: a population-based study. *BMC Geriatr* **18,** 317 (2018). https://doi.org/10.1186/s12877-018-1006-x
15. Walsh B, Laudicella M. Disparities in Cancer Care and Costs At The End Of Life: Evidence From England's National Health Service. *Health Aff (Millwood)*. 2017;36(7):1218-1226. doi:10.1377/hlthaff.2017.0167
16. Public Health Scotland, Patient Pathways: Emergency Care. Accessed: 24/4/2020 https://www.isdscotland.org/Health-Topics/Emergency-Care/Patient-Pathways/
17. World Health Organisation. International Statistical Classification of diseases and related health problems version 10 (ICD 10). https://www.who.int/classifications/icd/ICD10Volume2_en_2010.pdf
18. Moine, S. How to Better Visualise the Distribution of Expected Annual Deaths in a "Standard" French GP Surgery? Rebuilding the Three Main Illness Trajectories through a Modified Delphi Study. Abstract FC51. *Palliat Med* 2016. 30: NP1–NP401.
19. Scottish Government. The Scottish Index of Multiple Deprivation. Accessed: 24/4/2020 https://www2.gov.scot/Topics/Statistics/SIMD
20. Scottish Government. Scottish Government Urban Rural Classification 2016. (29 Mar 2018). Accessed 24/4/2020. https://www.gov.scot/publications/scottish-government-urban-rural-classification-2016/pages/2/
21. Tapsfield J, Hall C, Lunan C, et al. Many people in Scotland now benefit from anticipatory care before they die: an after death analysis and interviews with general practitioners. *BMJ Support Palliat Care*. 2019; 9(4):e28. doi:10.1136/bmjspcare-2015-001014.
22. Alsirafy SA, Raheem AA, Al-Zahrani AS, et al. Emergency department visits at the end of life of patients with terminal cancer: pattern, causes, and avoidability. *Am J Hosp Palliat Care* 2016. 33(7):658–662.

For peer review only - http://bmjopen.bmj.com/site/about/guidelines.xhtml

23. Mills S, Buchanan D, Guthrie B, et al. Factors affecting use of unscheduled care for people with advanced cancer: a retrospective cohort study in Scotland *BJGP* 2019; 69 (689): e860-e868. DOI: https://doi.org/10.3399/bjgp19X706637
24. Finucane AM, Davydaitis D, Horseman Z, Carduff E, et al. Electronic care coordination systems for people with advanced progressive illness: a mixed-methods evaluation in Scottish primary care. Br J Gen Pract. 2020. doi: 10.3399/bjgp19X707117
25. Smith, C., et al., Coordinate My Care: a clinical service that coordinates care, giving patients choice and improving quality of life. BMJ Support & Palliat Care, 2012. 2(4): p. 301-307.
26. Hawkes CA, Fritz Z, Deas G, et al. Development of the Recommended Summary Plan for Emergency Care and Treatment (ReSPECT), (2020). *Resuscitation*, 148: 98-107, https://doi.org/10.1016/j.resuscitation.2020.01.003.
27. Cooper, A., Carson-Stevens, A., Hughes, T., & Edwards, A. Is streaming patients in emergency departments to primary care services effective and safe? *BMJ*, 2020;368, m462. https://doi.org/10.1136/bmj.m462
28. Knaul, Felicia M et al. The Lancet Commission on Palliative Care and Pain Relief—findings, recommendations, and future directions, Volume 6, Special Issue, S5-S6, 1st March, 2018. https://doi.org/10.1016/S2214-109X(18)30082-2
29. Best S, Tate T, Noble B, et al. Research Priority Setting in Palliative and End of Life Care: The James Lind Approach Consulting Patients. *BMJ Support Palliat Care*. 2015;5(1):102 LP - 102. doi:10.1136/bmjspcare-2014-000838.

For peer review only

For peer review only - http://bmjopen.bmj.com/site/about/guidelines.xhtml

Table 1. Number (% and cumulative %) of patients (18+) in the last year of life in Scotland (2016) by number of contacts with unscheduled care services (N=56,407)

Number of service contacts	Number of patients	% Patients	Cumulative%
0	2,898	5.1	5.1
1	3,983	7.1	12.2
2	5,459	9.7	21.9
3	5,795	10.3	32.2
4	5,205	9.2	41.4
5	4,650	8.2	49.6
6	4,185	7.4	57.0
7	3,589	6.4	63.4
8	3,090	5.5	68.9
9	2,715	4.8	73.7
10	2,300	4.1	77.8
11	1,901	3.4	81.1
12	1,646	2.9	84.1
13	1,318	2.3	86.4
14	1,110	2.0	88.4
15	967	1.7	90.1
16	809	1.4	91.5
17	679	1.2	92.7
18	570	1.0	93.7
19	520	0.9	94.6
20+	3,018	5.4	100
Total	56,407	100	

For peer review only

For peer review only - http://bmjopen.bmj.com/site/about/guidelines.xhtml

Table 2. Number (and percentage) of people who had contacts with NHS telephone advice (NHS24), Primary Care Out-of-Hours (PCOOH), Scottish Ambulance Service (SAS), Emergency Department (ED), and Hospital admission for patients (18+) in the last year of life in Scotland (2016) by underlying cause of death, deprivation, place of residence, urban/rural classification (N=56,407)

		NHS24		PCOOH		SAS		ED attendance		Hospital admission	
	All deceased persons	% with contact	Number with contact	% with contact	Number with contact	% with contact	Number with contact	% with attendance	Number with attendance	% with admission	Number with admission
**Total***	56,407	59.7	33,656	53.5	30,161	73.9	41,678	62.7	35,383	74.9	42,253
**Cause of death**											
Cancer	15,902	63.4	10,074	61.5	9,783	72.8	11,569	62.0	9,857	88.3	14,039
Organ failure	21,244	56.0	11,888	45.6	9,678	78.9	16,770	65.2	13,851	73.2	15,559
Frailty	14,023	66.0	9,258	61.7	8,654	67.8	9,509	58.9	8,262	66.4	9,314
Various	2,271	56.9	1,292	48.9	1,111	76.4	1,735	64.9	1,474	74.1	1,683
External	2,967	38.5	1,142	31.5	935	70.6	2,095	65.3	1,937	55.9	1,659
p-value			<.001		<.001		<.001		<.001		<.001
**Deprivation**											
Most	13,537	59.8	8,093	46.6	6,307	77.2	10,449	67.3	9,111	76.4	10,347
2	12,812	59.8	7,665	52.5	6,720	76.9	9,850	66.5	8,517	77.2	9,892
3	11,747	59.2	6,959	55.8	6,558	72.3	8,498	60.0	7,050	73.9	8,680
4	9,840	59.8	5,883	58.8	5,790	71.5	7,032	58.7	5,775	72.9	7,177
Least	8,315	60.2	5,002	56.9	4,731	69.4	5,774	58.4	4,853	72.7	6,044
p-value			<.758		<.001		<.001		<.001		<.001
**Place of residence**											
Home/Private	47,813	58.0	27,751	50.7	24,221	77.2	36,891	65.6	31,366	79.3	37,907
Institution	8,558	68.8	5,891	69.3	5,928	55.7	4,770	46.7	4,000	50.6	4,328
p-value			<.001		<.001		<.001		<.001		<.001
**Urban/rural**											
Large urban areas	18,750	60.6	11,359	49.0	9,183	74.9	14,053	63.3	11,868	75.4	14,141
Other urban areas	20,372	61.4	12,505	53.9	10,986	75.0	15,270	66.8	13,612	75.6	15,407
Accessible small towns	5,244	57.7	3,026	55.9	2,934	74.1	3,884	62.9	3,297	74.9	3,926
Remote small towns	2,367	56.3	1,332	59.3	1,404	71.6	1,694	54.8	1,296	72.7	1,721
Accessible Rural	5,989	59.6	3,570	58.9	3,526	71.9	4,304	59.1	3,542	74.3	4,447

For peer review only

Remote Rural	3,529	51.3	1,810	58.7	2,073	68.0	2,398	47.9	1,691	70.8	2,498
p-value			<.001		<.001		<.001		<.001		<.001

* The deprivation, place of residence and urban/rural categories do not add up to 56,407 due to incomplete data. Each of these categories has less than 0.3% missing data.

For peer review only

For peer review only - http://bmjopen.bmj.com/site/about/guidelines.xhtml

Table 3. Adjusted odds ratios (OR) and 95% confidence intervals for the probability of contacts with : NHS telephone advice (NHS24), Primary Care Out-of-Hours (PCOOH), Scottish Ambulance Service (SAS), Emergency Department (ED) and Hospital Admission (HA) for patients (18+) in their last year of their life (N=56,112)*

	NHS24	PCOOH	SAS	ED	HA
	OR (95%CI)	OR (95%CI)	OR (95%CI)	OR (95%CI)	OR (95%CI)
**Sex**					
Male	1	1	1	1	1
Female	1.17 (1.12-1.21)	1.14 (1.10-1.19)	0.93 (0.88-0.96)	0.93 (0.90-0.97)	1.06 (1.02-1.11)
**Age**					
Age 18-64	1	1	1	1	1
Age 65-84	1.22 (1.16-1.28)	1.18 (1.12-1.24)	1.42 (1.34-1.50)	1.17 (1.11-1.23)	1.68 (1.58-1.78)
Age 85+	1.61 (1.51-1.71)	1.61 (1.52-1.71)	1.43 (1.34-1.53)	1.12 (1.05-1.19)	1.67 (1.56-1.79)
**Marital status**					
Single	1	1	1	1	1
Married	1.20 (1.14-1.27)	1.24 (1.17-1.31)	1.13 (1.06-1.20)	1.22 (1.15-1.30)	1.38 (1.29-1.47)
Widowed	1.27 (1.20-1.35)	1.28 (1.21-1.36)	1.13 (1.06-1.21)	1.24 (1.16-1.31)	1.36 (1.27-1.46)
Divorced	1.11 (1.04-1.19)	1.12 (1.04-1.20)	1.11 (1.03-1.20)	1.14 (1.06-1.22)	1.28 (1.19-1.39)
**Deprivation**					
Most Deprived	1.16 (1.09-1.23)	0.82 (0.78-0.87)	1.42 (1.33-1.51)	1.39 (1.31-1.48)	1.35 (1.26-1.44)
Q2	1.13 (1.06-1.19)	0.94 (0.94-0.95)	1.39 (1.31-1.49)	1.35 (1.27-1.43)	1.32 (1.23-1.41)
Q3	1.10 (1.04-1.17)	0.96 (0.96-0.97)	1.20 (1.13-1.28)	1.16 (1.09-1.23)	1.17 (1.09-1.25)
Q4	1.08 (1.01-1.15)	1.05 (1.05-1.05)	1.17 (1.10-1.25)	1.11 (1.04-1.18)	1.11 (1.04-1.19)
Least deprived	1	1	1	1	1
**Cause of death**					
Organ failure	1	1	1	1	1
Cancer	1.45 (1.39-1.52)	2.08 (1.99-2.17)	0.67 (0.64-0.70)	0.81 (0.78-0.85)	2.56 (2.28-2.86)
Frailty	1.27 (1.21-1.33)	1.44 (1.38-1.51)	0.71 (0.64-0.79)	0.77 (0.69-0.85)	0.93 (0.88-0.97)
**Urban/rural**					
Large urban areas	1.53 (1.42-1.65)	0.72 (0.67-0.78)	1.47 (1.35-1.60)	1.92 (1.77-2.07)	1.34 (1.23-1.46)
Other urban areas	1.55 (1.44-1.67)	0.88 (0.84-0.91)	1.43 (1.32-1.55)	2.24 (2.08-2.42)	1.35 (1.24-1.47)
Accessible small towns	1.33 (1.22-1.45)	0.93 (0.91-0.94)	1.38 (1.25-1.52)	1.90 (1.74-2.08)	1.26 (1.14-1.40)
Remote small towns	1.22 (1.10-1.36)	1.03 (1.04-1.02)	1.23 (1.09-1.38)	1.36 (1.22-1.51)	1.16 (1.03-1.31)
Accessible rural	1.44 (1.32-1.57)	1.01 (0.99-1.02)	1.22 (1.11-1.34)	1.60 (1.47-1.75)	1.15 (1.04-1.26)
Remote rural	1	1	1	1	1
**Place of residence**					
Institution	1	1	1	1	1
Home	1.31 (1.24-1.39)	1.89 (1.79-1.99)	2.72 (2.58-2.87)	2.33 (2.21-2.45)	3.39 (3.21-3.57)

Reference categories are male, age18-64, single, least deprived, organ failure, remote rural and institution (place of residence).

*295 patients in "Other/Unknown" categories excluded. "Various" and "External" causes of death not shown in table.

For peer review only - http://bmjopen.bmj.com/site/about/guidelines.xhtml

Table 4. Twenty most frequent Continuous Unscheduled Pathways (CUPs) by in-hours or out-of-hours start time for patients (18+) in the last year of life in Scotland in 2016 (N=56,407)

	In-hours CUPs (n=58,157)				Out-of-hours CUPs (n=133,980)			
Rank	Name	Number	%	CUM %	Name	Number	%	CUM %
1	SEA	13,702	23.6	23.6	NO	30,434	22.7	22.7
2	SA	8,639	14.9	38.5	O	18,609	13.9	36.6
3	EA	6,408	11.0	49.5	SEA	9,457	7.1	43.7
4	S	6,014	10.3	59.8	N	8,868	6.6	50.3
5	E	5,085	8.7	68.5	EA	4,923	3.7	54.0
6	SE	3,291	5.7	74.2	NSEA	4,866	3.6	57.6
7	NO	1,450	2.5	76.7	S	4,028	3.0	60.6
8	N	1,230	2.1	78.8	E	3,890	2.9	63.5
9	O	1,005	1.7	80.5	SE	2,816	2.1	65.6
10	NSEA	561	1.0	81.5	NOSEA	2,743	2.0	67.6
11	SEAS	484	.80	82.3	OO	2,697	2.0	69.6
12	SAO	366	.60	82.9	NOSA	2,306	1.7	71.3
13	SAS	305	.50	83.4	NONO	1,677	1.3	72.6
14	SEAO	305	.50	83.9	NON	1,517	1.1	73.7
15	SSEA	285	.50	84.4	NSE	1,305	1.0	74.7
16	SSA	239	.40	84.8	NOO	1,162	.90	75.6
17	SS	229	.40	85.2	NOA	1,112	.80	76.4
18	EAS	221	.40	85.6	SA	1,042	.80	77.2
19	SES	220	.40	86.0	NS	948	.70	77.9
20	NSE	164	.30	86.3	NOEA	924	.70	78.6

* 14,704 missing time stamps

Abbreviations: N=NHS 24, O= Primary Care Out-of-Hours, S=Scottish Ambulance Service, E=Emergency Department, A= Acute hospital admission

For example, the CUP “SEA” represents a contact with the Scottish Ambulance Service (S), followed by an attendance at an emergency department (E), then an unscheduled hospital admission (A).

For peer review only - http://bmjopen.bmj.com/site/about/guidelines.xhtml

Table 5. Mean number of contacts and costs of NHS telephone advice (NHS24), Primary Care Out-of-Hours (PCOOH), Scottish Ambulance Service (SAS), Emergency Department (ED), and Hospital admissions for patients (18+) in the last year of life in Scotland (2016) by cause of death and deprivation status (N=56,407)

	NHS24			PCOOH			SAS			Emergency department			Hospital Admissions			**All contacts**	
	Mean number of contacts	Total costs (x1000)	Costs per patient	Mean number of contacts	Total costs (x1000)	Costs per patient	Mean number of calls	Total costs (x1000)	Costs per patient	Mean number of attendances	Total costs (x1000)	Costs per patient	Mean number of admissions	Total costs (x1000)	Costs per patient	**Total costs to NHS (x1000)**	**Total cost to NHS per patient**
**Total**	1.4	£2,944	£52	1.3	£4,471	£79	1.7	£22,337	£396	1.3	£10,205	£181	1.7	£149,967	£2,659	**£189,923**	**£3,367**
**Cause of death**																	
Cancer	1.4	£836	£53	1.6	£1,502	£94	1.6	£5,807	£365	1.3	£2,765	£174	2.1	£54,022	£3,397	**£64,933**	**£4,083**
Organ failure	1.3	£1,047	£49	1.1	£1,370	£64	2.0	£9,525	£448	1.4	£4,173	£196	1.7	£56,730	£2,670	**£72,845**	**£3,429**
Frailty	1.7	£842	£60	1.6	£1,315	£94	1.5	£4,892	£349	1.1	£2,165	£154	1.3	£28,003	£1,997	**£37,218**	**£2,654**
**Deprivation**																	
Most Deprived	1.5	£748	£55	1.1	£794	£59	1.9	£5,960	£440	1.5	£2,867	£212	1.8	£38,809	£2,867	**£49,177**	**£3,633**
2	1.5	£678	£53	1.3	£984	£77	1.9	£5,543	£433	1.4	£2,559	£200	1.8	£35,805	£2,795	**£45,569**	**£3,557**
3	1.4	£607	£52	1.5	£1,048	£89	1.6	£4,390	£374	1.2	£1,936	£165	1.6	£29,625	£2,522	**£37,606**	**£3,201**
4	1.4	£501	£51	1.6	£929	£94	1.6	£3,644	£370	1.2	£1,569	£159	1.6	£25,050	£2,546	**£31,693**	**£3,221**
Least deprived	1.4	£405	£49	1.4	£710	£85	1.5	£2,766	£333	1.1	£1,256	£151	1.5	£20,349	£2,447	**£25,487**	**£3,065**

For peer review only

For peer review only - http://bmjopen.bmj.com/site/about/guidelines.xhtml

Figure 1 – Start and end points of all unscheduled care pathways

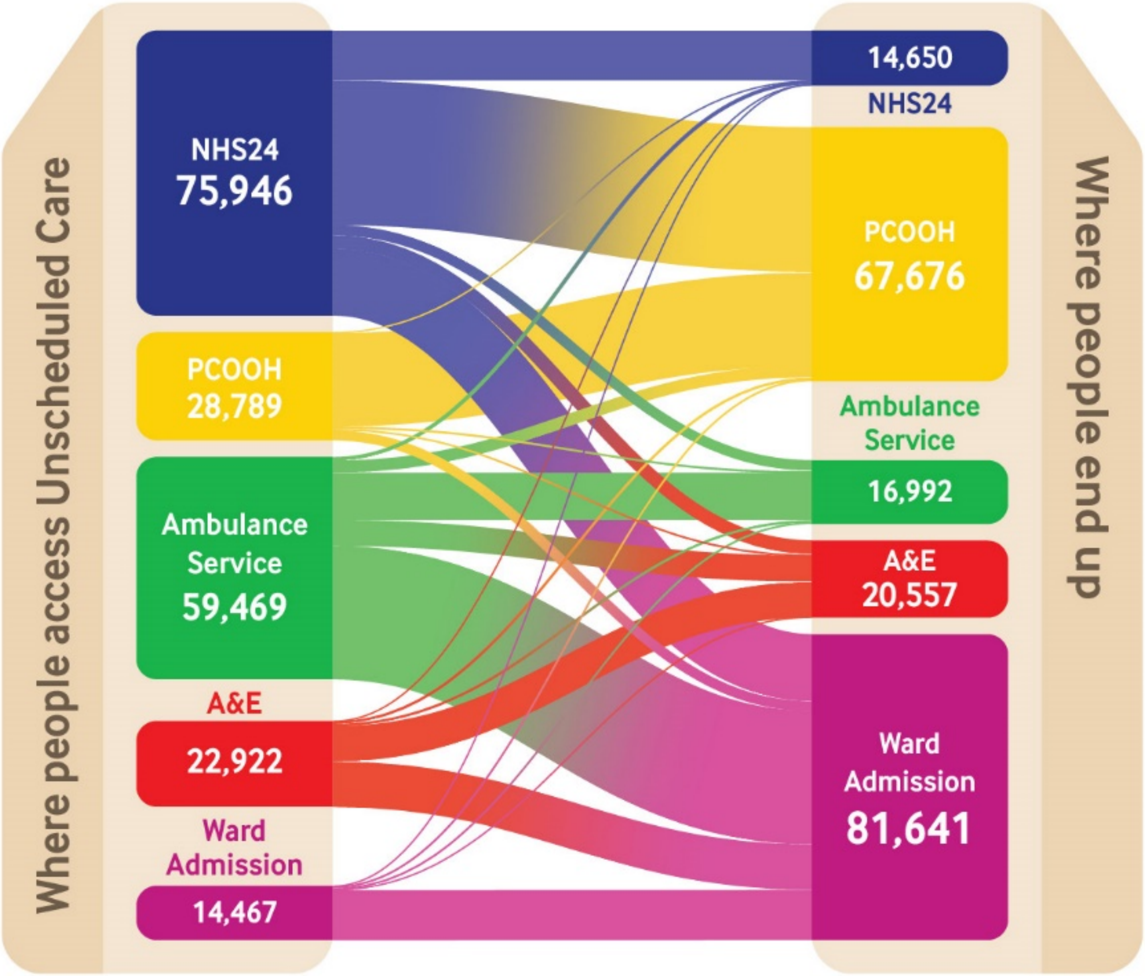

This figure shows the first and final service contacted in each Continuous Unscheduled Care Pathway (CUP). A first contact of telephone advice (NHS24) or Primary Care out-of-hours (PCOOH) was likely to be resolved in primary care. Calling an ambulance or attendance at emergency department (A&E) usually resulted in admission.

- NHS24 = telephone advice
- PCOOH= Primary Care Out of Hours
- A&E=Emergency Department

Note The figure shows 14,467 direct admissions to hospital which were likely to have been via primary care in-hours or outpatient services. Some may also be due to other direct admission routes such as the Scottish Cancer helpline for people receiving cancer treatments.

For peer review only - http://bmjopen.bmj.com/site/about/guidelines.xhtml

Supplementary tables 1-4

Supplementary table 1. List of ICD10 codes for underlying causes of death grouped into three main categories of conditions with potential palliative care needs (referred to as "illness trajectories")

Illness trajectories [ITs]	Sub-ITs	ICD10 codes (v.2010)
**IT1. Cancer *("Clear terminal phase")***		
	1.1 Malignant Neoplasms	C00-C97
**IT2. Organ failure *("Intermittent")***		
	2.1 Endocrine and metabolic diseases	E00-E90<br>*Except: E46*
	2.2 Diseases of the circulatory system	I00-I99<br>*Except: I46*<br>*(I60-I67)$_{[65+]}$* ****(1/3)*** [1]<br>*I69*
	2.3 Diseases of the respiratory system	J00-J99.8<br>*Except: (J06, J09-J16, J18)$_{[65+]}$* ****(1/3)*** [2]<br>*(J20-J22)$_{[65+]}$* ****(1/3)*** [3]<br>*J69.0, J96*
	2.4 Diseases of liver	K70-K77, B18
	2.5 Kidney disease	N17, N18, N28
	2.6 Diseases of the digestive system	K00-K93.8<br>*Except: K70-K77*
	2.7 Other diseases of the genitourinary system	N00-N99.9<br>*Except: N17, N18, N28*
**IT3a. Frailty, Progressive neurological condition [80+]**		

For peer review only

("Gradually dwindling")		
	3.1 Dementia and mental disorders [80+]	A81.0, F01, F03-F07, F09, G30, G31
	3.2 Progressive neurological diseases [80+]	G10, G12.2, G20, G35, G23.1, G90.3
	3.3 Cerebrovascular diseases [80+]	(I60-I67)$_{[80+]}$***(1/3)**, I69
	3.4 Some ill-defined causes [80+]	R26, R29.6, R53, R54, R55, R63, R64
		I46, J96, R96, R98, R99, W00, W03-08, W10, W18
	3.5 Malnutrition [80+]	E46
	3.6 Infectious causes / Influenza / Pneumonia [80+]	A15-A19.9, B90-B90.9, B20-B24
		(J06, J09-J16, J18)$_{[80+]}$***(1/3)**
		(J20-J22)$_{[80+]}$***(1/3)**, J69.0
	3.7 Skin and musculo-skeletal system [80+]	L00-L99.8, M00-M99.9
**IT3b. Frailty, Progressive neurological condition [65-79]** ***("Gradually dwindling")***		
	3.8 Dementia and mental disorders [65-79]	A81.0, F01, F03-F07, F09, G30, G31
	3.9 Progressive neurological diseases [65-79]	G10, G12.2, G20, G35, G23.1, G90.3
	3.10 Cerebrovascular diseases [65-79]	(I60-I67)$_{[65-79]}$***(1/3)**, I69
	3.11 Some ill-defined causes [65-79]	R26, R29.6, R53, R54, R55, R63, R64, I46, J96
	3.12 Malnutrition [65-79]	E46
	3.13 Infectious causes / Influenza / Pneumonia [65-79]	A15-A19.9, B90-B90.9, B20-B24
		(J06, J09-J16, J18)$_{[65-79]}$***(1/3)**
		(J20-J22)$_{[65-79]}$***(1/3)**
		J69.0
	3.14 Skin and musculo-skeletal system [65-79]	L00-L99.8, M00-M99.9

For peer review only

IT3c. Frailty, Progressive neurological condition [18-64]

("Gradually dwindling")

	3.15 Dementia and mental disorders [18-64]	A81.0, F01, F03-F07, F09, G30, G31
	3.16 Progressive neurological diseases [18-64]	G10, G12.2, G20, G35, G23.1, G90.3
	3.17 Cerebrovascular diseases [18-64]	I69
	3.18 Some ill-defined causes [18-64]	R26, R29.6, R53, R54, R55, R63, R64, I46, J96
	3.19 Malnutrition [18-64]	E46
	3.20 Infectious causes / Influenza / Pneumonia [18-64]	A15-A19.9, B90-B90.9, B20-B24, J69.0
	3.21 Skin and musculo-skeletal system [18-64]	L00-L99.8, M00-M99.9

4. Various causes

	4.1 Infectious diseases	A00-B99.9 *Except: A81.0, A15-A19.9, B18, B20-B24, B90-B90.9*
	4.2 Neoplasms + Diseases of the blood	D01-D89.9
	4.3 Diseases of the nervous system - *except progressive neurological diseases*	G00-H95.9 *Except: G10, G12.2, G20, G23.1, G30, G31, G35, G90.3*
	4.4 Symptoms, signs... not elsewhere classified / Ill-defined causes - *except R26, R29.6, R53, R54, R55, R63, R64, and (R96, R98, R99)[80+]*	R00-R99 *Except: R26, R29.6, R53, R54, R55, R63, R64, (R96, R98, R99)[80+]*
	4.5 Substance abuse and other mental and behavioral disorders	F10-F98
	4.7 Pregnancy, childbirth, puerperium / Congenital malformation	O00-O998, P00-P96.9, Q00-Q99.9

For peer review only

5. External causes

	5. External causes of morbidity and mortality	V01-Y89.9<br>*Except:(W00, W03-08, W10, W18)[80+]*

---

[1] One third of the annual deaths with ICD-10 codes (I60-I67) as underlying cause of death (people over 65)

[2] One third of the annual deaths with ICD-10 codes (J06, J09-J16, J18) as underlying cause of death (people over 65)

[3] One third of the annual deaths with ICD-10 codes (J20-J22) as underlying cause of death (people over 65)

The characteristics of the main disease responsible for the death of a patient may be associated with certain health services utilisation patterns in the last months of life. Rather than using every single disease used for underlying causes of death in the International Statistical Classification of Diseases and Related Health Problems ("ICD-10"), we used categories of life-limiting conditions that have been described as having similar courses until death.

The conditions were grouped into three "illness trajectories" ("cancer", "organ failure", "frailty/ progressive neurological conditions"). These trajectories have a shape and an average duration, and they are associated with potential palliative care needs in the people they affect (whether physically, psychologically, socially, existentially or spiritually).

Our goal for this study was to allocate ICD-10 codes used for underlying causes of death in mortality datasets, to these three main categories of conditions ("illness trajectories").

The underlying cause of death is defined as the disease or injury which initiated the chain of events leading directly to death. Every death has just one underlying cause, so is counted only once in figures which are produced on this basis. Deaths are coded in accordance with the ICD-10.

The allocation of ICD-10 codes for underlying causes of death to the three main illness trajectories was completed prior to this study by a panel of international experts in primary care, palliative care, public health and demography, as part of a modified e-Delphi study carried out by two authors of this article (SAM and SM). The panel did not consider contributory causes of death. The list of ICD-10 codes used in this current article has been obtained after two Delphi rounds, and it consists in seven categories (as displayed in Table 1):

1. Cancer
2. Organ failure
3. Frailty/ Progressive neurological conditions [80+]
4. Frailty/ Progressive neurological conditions [65-79]
5. Frailty/ Progressive neurological conditions [18-64]
6. Various causes
7. External causes

The allocation of the total number of deaths to each main category (Scotland, 2016) is featured in Supplementary table 3

For peer review only

Supplementary table 2. Price weight allocation for cost analysis.

We allocated a fixed value for each service used based on average values.

Unscheduled Service	Value	Unit	Notes/Sources
***N**: NHS24 CONTACTS*			
NHS 24 Contact	£30.78	per call	Private communication with NHS24.
***O**: OOH PRIMARY CARE CONSULTATIONS*			
GP OOH	£38.00	per consultation	[note 1]
Nurse OOH	£6.80	per consultation	£0.70 per min x 9.72 mins. [note 1]
OOH Average Unspecified "O"	£33.20	per consultation	Weighted average of GP/Nurse consultation applied where GP/Nurse consultations were pooled. Based on ratio observed in data.
***S**: SCOTTISH AMBULANCE SERVICE (SAS)*			
Conveyed	£238.00	per trip	Applied to a S with an E or A in the CUP [note 2]
Not Conveyed	£184.00	per trip	Applied to a S without an E or A in the CUP [note 2]
OOH Average Unspecified "S"	£228.89	per trip	Weighted average of conveyed/not conveyed based on observed ratio used. Applied where A or E presence is unspecified.
***E**: EMERGENCY DEPT*			
Accident and Emergency Visit	£138.00	per visit	[note 3]
***A**: INPATIENT ADMISSIONS*			
Emergency Inpatient Admissions	£1,590.00	Per Admission	Average non-elective inpatient admission (excluding excess bed days) [note 3].

Notes

Two factors made costing emergency inpatient admissions for this cohort difficult. Firstly, the Scottish data does not include detailed costs for patient procedures we took a general population average cost. Secondly, we did not cost according to length of stay. It is possible that people who died from different underlying causes may have required different average lengths of stay. Some of the cohort may have required longer stays than patients from the general population and possibly more expensive or multiple interventions than the general population. Although the precise figures are uncertain, it is undoubtedly true that any hospital admission is an order of magnitude more expensive than any other unscheduled care service.

1. Curtis L et al. Unit Costs of Health & Social Care 2017. Kent: Personal Social Services Research Unit 2017
2. Hobbs R, Bankhead C, Mukhtar T, Stevens S, Perera-Salazar R, Holt T, & Salisbury C. (2016) Clinical workload in UK primary care: a retrospective analysis of 100 million consultations in England, 2007-14, The Lancet, 387, 10035, 2323-2330. http://www.sciencedirect.com/science/article/pii/S0140673616006206.
3. Department of Health, The. Reference Costs 2016-17 Publication. London: The Department of Health 2017

Supplementary table 3

For peer review only

Supplementary table 3. Number (%) of patients (18+) in the last year of life in Scotland 2016 by underlying cause of death (N=56,407)

Underlying cause of death	N	%
**Cancer (all types)**	**15,902**	**28.2%**
**Organ failure**	**21,244**	**37.7%**
2.1 Endocrine and metabolic	1,137	
2.2 Circulatory	11,212	
2.3 Respiratory	4,744	
2.4 Liver	1,109	
2.5 Kidney	241	
2.6 Digestive	1,949	
2.7 Other genitourinary	852	
**Frailty 80+**	**10,497**	**18.6%**
3.1 Dementia 80+	4,857	
3.2 Progressive neurological 80+	360	
3.3 Cerebrovascular 80+	2,739	
3.4 Ill-defined 80+	258	
3.4 Malnutrition 80+	3	
3.6 Infections & Pneumonia 80+	1,971	
3.7 Skin & Musculo-skeletal 80+	309	
**Frailty 65-79**	**3,076**	**5.5%**
3.8 Dementia 65-79	932	
3.9 Progressive neurological 65-79	332	
3.10 Cerebrovascular 65-79	1,042	
3.11 Ill-defined 65-79	14	
3.12 Malnutrition 65-79	3	
3.13 Infections & Pneumonia 65-79	572	
3.14 Skin & Musculo-skeletal 65-79	181	
**Frailty 19-64**	**450**	**0.8%**
3.15 Dementia 19-64	55	
3.16 Progressive neurological 19-64	180	
3.17 Cerebrovascular 19-64	50	
3.18 Ill-defined 19-64	16	
3.19 Malnutrition 19-64	2	
3.20 Infections & Pneumonia 19-64	63	
3.21 Skin & Musculo-skeletal 19-64	84	
**Various**	**2,271**	**4.0%**
4.1 Infections	648	
4.2 Neoplasms & blood	456	
4.3 Nervous system	395	
4.4 Symptoms & Signs NEC	235	
4.5 Mental & behavioural	419	
4.7 Pregnancy & congenital	118	
**External**	**2,967**	**5.3%**

For peer review only

Total	56,407	100.0%

Supplementary Table 4. Number (%) of contacts with services in each of the 12 months before death by patients (18+) in Scotland 2016 (Month 12 represents the 12th month before death)

	Total		Contact NHS 24		Contact PCOOH		Call to SAS		ED attendance		Emergency admissions	
Months	Number	%	Number	%	Number	%	Number	%	Number	%	Number	%
12	18,385	3.9	4,109	4.3	3,584	3.3	3,602	3.8	3,632	4.5	3,458	3.8
11	19,327	4.1	4,200	4.4	3,687	3.4	3,811	4.0	3,919	4.8	3,710	4.0
10	19,779	4.2	4,374	4.6	3,889	3.6	3,866	4.1	3,942	4.9	3,708	4.0
9	21,136	4.5	4,679	4.9	3,993	3.7	4,056	4.3	4,236	5.2	4,172	4.5
8	22,374	4.7	4,818	5.1	4,306	3.9	4,375	4.6	4,353	5.4	4,522	4.9
7	24,006	5.1	5,164	5.4	4,681	4.3	4,667	4.9	4,625	5.7	4,869	5.3
6	25,542	5.4	5,391	5.7	5,022	4.6	5,102	5.4	4,832	6.0	5,195	5.7
5	28,759	6.1	5,902	6.2	5,652	5.2	5,728	6.0	5,463	6.7	6,014	6.6
4	33,334	7.1	6,579	6.9	6,624	6.1	6,725	7.1	6,250	7.7	7,156	7.8
3	41,634	8.8	7,955	8.3	8,319	7.6	8,616	9.1	7,550	9.3	9,194	10.0
2	56,473	12.0	10,428	10.9	11,567	10.6	11,888	12.5	9,781	12.1	12,809	14.0
1	161,611	34.2	31,751	33.3	47,833	43.8	32,583	34.3	22,488	27.7	26,956	29.4
Total	472,360	100	95,350	100	109,157	100	95,019	100	81,071	100	91,763	100

For peer review only

STROBE (Strengthening The Reporting of OBservational Studies in Epidemiology) Checklist

A checklist of items that should be included in reports of observational studies. You must report the page number in your manuscript where you consider each of the items listed in this checklist. If you have not included this information, either revise your manuscript accordingly before submitting or note N/A.

Note: An Explanation and Elaboration article discusses each checklist item and gives methodological background and published examples of transparent reporting. The STROBE checklist is best used in conjunction with this article (freely available on the Web sites of PLoS Medicine at http://www.plosmedicine.org/, Annals of Internal Medicine at http://www.annals.org/, and Epidemiology at http://www.epidem.com/). Information on the STROBE Initiative is available at www.strobe-statement.org.

Section and Item	Item No.	Recommendation	Reported on Page No.
**Title and Abstract**	1	(*a*) Indicate the study's design with a commonly used term in the title or the abstract	
		(*b*) Provide in the abstract an informative and balanced summary of what was done and what was found	
**Introduction**			
Background/Rationale	2	Explain the scientific background and rationale for the investigation being reported	
Objectives	3	State specific objectives, including any prespecified hypotheses	
**Methods**			
Study Design	4	Present key elements of study design early in the paper	
Setting	5	Describe the setting, locations, and relevant dates, including periods of recruitment, exposure, follow-up, and data collection	
Participants	6	(*a*) *Cohort study*—Give the eligibility criteria, and the sources and methods of selection of participants. Describe methods of follow-up<br>*Case-control study*—Give the eligibility criteria, and the sources and methods of case ascertainment and control selection. Give the rationale for the choice of cases and controls<br>*Cross-sectional study*—Give the eligibility criteria, and the sources and methods of selection of participants	
		(*b*) *Cohort study*—For matched studies, give matching criteria and number of exposed and unexposed<br>*Case-control study*—For matched studies, give matching criteria and the number of controls per case	
Variables	7	Clearly define all outcomes, exposures, predictors, potential confounders, and effect modifiers. Give diagnostic criteria, if applicable	

For peer review only

Section and Item	Item No.	Recommendation	Reported on Page No.
Data Sources/ Measurement	8*	For each variable of interest, give sources of data and details of methods of assessment (measurement). Describe comparability of assessment methods if there is more than one group	
Bias	9	Describe any efforts to address potential sources of bias	
Study Size	10	Explain how the study size was arrived at	
Quantitative Variables	11	Explain how quantitative variables were handled in the analyses. If applicable, describe which groupings were chosen and why	
Statistical Methods	12	(*a*) Describe all statistical methods, including those used to control for confounding	
		(*b*) Describe any methods used to examine subgroups and interactions	
		(*c*) Explain how missing data were addressed	
		(*d*) *Cohort study*—If applicable, explain how loss to follow-up was addressed<br>*Case-control study*—If applicable, explain how matching of cases and controls was addressed<br>*Cross-sectional study*—If applicable, describe analytical methods taking account of sampling strategy	
		(*e*) Describe any sensitivity analyses	
**Results**			
Participants	13*	(a) Report numbers of individuals at each stage of study—eg numbers potentially eligible, examined for eligibility, confirmed eligible, included in the study, completing follow-up, and analysed	
		(b) Give reasons for non-participation at each stage	
		(c) Consider use of a flow diagram	
Descriptive Data	14*	(a) Give characteristics of study participants (eg demographic, clinical, social) and information on exposures and potential confounders	
		(b) Indicate number of participants with missing data for each variable of interest	
		(c) *Cohort study*—Summarise follow-up time (eg, average and total amount)	
Outcome Data	15*	*Cohort study*—Report numbers of outcome events or summary measures over time	
		Case-control study—Report numbers in each exposure category, or summary measures of exposure	
		Cross-sectional study—Report numbers of outcome events or summary measures	

For peer review only

Section and Item	Item No.	Recommendation	Reported on Page No.
Main Results	16	(*a*) Give unadjusted estimates and, if applicable, confounder-adjusted estimates and their precision (eg, 95% confidence interval). Make clear which confounders were adjusted for and why they were included	
		(*b*) Report category boundaries when continuous variables were categorized	
		(*c*) If relevant, consider translating estimates of relative risk into absolute risk for a meaningful time period	
Other Analyses	17	Report other analyses done—eg analyses of subgroups and interactions, and sensitivity analyses	
**Discussion**			
Key Results	18	Summarise key results with reference to study objectives	
Limitations	19	Discuss limitations of the study, taking into account sources of potential bias or imprecision. Discuss both direction and magnitude of any potential bias	
Interpretation	20	Give a cautious overall interpretation of results considering objectives, limitations, multiplicity of analyses, results from similar studies, and other relevant evidence	
Generalisability	21	Discuss the generalisability (external validity) of the study results	
**Other Information**			
Funding	22	Give the source of funding and the role of the funders for the present study and, if applicable, for the original study on which the present article is based	

*Give information separately for cases and controls in case-control studies and, if applicable, for exposed and unexposed groups in cohort and cross-sectional studies.

Once you have completed this checklist, please save a copy and upload it as part of your submission. DO NOT include this checklist as part of the main manuscript document. It must be uploaded as a separate file.

For peer review only
